# Supplementary material for: Genome-wide sequencing of longan (Dimocarpus longan Lour.) provides insights into molecular basis of its polyphenol-rich characteristics
Source: Gigascience. 2017 Mar 28;6(5):1–14. doi: 10.1093/gigascience/gix023 (PMC5467034; doi:10.1093/gigascience/gix023)
Supplement: GIGA-D-16-00042_Original_Submission.pdf [file gix023_GIGA-D-16-00042_Original_Submission.pdf]

Manuscript
Click here to download Manuscript 2016-6-25 longan MS submission.doc
Click here to view linked References

Sequencing of longan (*Dimocarpus longan* Lour.) provides insights into molecular basis of its polyphenol-rich characteristics

YuLing Lin[1†], JiuMeng Min[2 †], RuiLian Lai[1], ZhangYan Wu[2], YuKun Chen[1], LiLi Yu[2], ChunZhen Cheng[1], YuanChun Jin[2], QiLin Tian[1], QingFeng Liu[2], WeiHua Liu[1], ChengGuang Zhang[2], LiXia Lin[1], YanHu[2], DongMin Zhang[1], MinKyaw Thu[1], ZiHao Zhang[1], ShengCai Liu[1], ChunShui Zhong[1], XiaoDong Fang[2], Jian Wang[2, 3], Huanming Yang[2, 3], RajeevK Varshney[4,5*], YeYin[2*], ZhongXiong Lai[1*]

[1]Institute of Horticultural Biotechnology, Fujian Agriculture and Forestry University, Fuzhou, Fujian 350002, China.

[2]BGI-Shenzhen, Shenzhen 518083, China.

[3] James D. Watson Institute of Genome Sciences, Hangzhou 310058, China

[4]International Crops Research Institute for the Semi-Arid Tropics (ICRISAT), Hyderabad, India.

[4]School of Plant Biology, the University of Western Australia, Crawley, Perth, Asutralia.

[†]These authors contributed equally to this work.

*Corresponding author

Email: R.K.Varshney@CGIAR.ORG, yinye@genomics.cn, laizx01@163.com.

Abstract

Background: Longan (*Dimocarpus longan* Lour.), an important subtropical fruit, is grown in more than 10 countries of the world, especially in China. China's longan acreage, and production accounts for 70 % and more than 50 % of the world's, ranking first. Its fruit, because of the sweet flavor and the polyphenol-rich, is preferably eaten fresh and popular used as bioactive ingredients in traditional Chinese medicines. Here, the draft genome sequence of longan and the extent of genetic diversity based on whole genome re-sequencing of 13 cultivated *D. longan* accessions have been presented for the first time.

Results: We present the draft genome (~471.88 Mb) of a China longan variety. We estimated 39,282 genes and characterized 261.88Mb repetitive sequences. Interesting, recent whole-genome wide duplication events were not observed in longan genome. Furthermore, whole-genome resequencing and analysis of 13 cultivated *D. longan* accessions identified the extent of genetic diversity and breeding-associated balancing selection. Comparative transcriptome studies combined with the genome-wide analysis revealed the polyphenol-rich and pathogen resistance characteristics of longan. The genes involvement of secondary metabolism, especially *F3'H*, *ANR*, and *LAR*, with higher copy numbers and tissue-specific expression, seem to be an important contributor to the high levels of polyphenolic compounds accumulation in longan fruit. A higher number of NBS-LRR, and LRR-RLK encoding genes, and the recent expansion and contractions of the NBS-LRR family in the longan genome may suggest a genomic basis for resistance to insects, fungus and bacteria in this fruit tree.

Conclusions: These data provide insights into evolution and diversity of the longan genome; comparative genomic and transcriptome analyses, provided insights into longan specific traits,

particularly those involved in polyphenol-rich and pathogen resistance characteristics.

Keywords: de novo genome sequence - genetic diversity - polyphenols biosynthesis - pathogen resistance

Background

Dimocarpus longan Lour. (*D. longan*), with a diploid genome (2*n*=2*x*=30), originating from South China or Southeastern Asia, commonly called longan or 'dragon eye' in the orient, is an important tropical/subtropical evergreen fruit tree belonging to the family Sapindaceae, widely cultivated southeastern Asia, South Asia, Australia and Hawaii of USA, etc, especially in China [1]. China's longan acreage, and production accounts for 70 % and more than 50 % of the world's, respectively, ranking first [2]. As an edible drupe fruit and source of traditional medicine, longan is grown in most areas of Southern China, such as Guangdong, Guangxi, Fujian, Sichuan, Yunnan, and Hainan [3]. Traditionally, longan leaves, flowers, fruit, and seeds, have been widely used as a traditional Chinese medicine for several diseases including leucorrhea, kidney disorders, allergies, cancer, diabetes and cardiovascular, due to they contain bioactive compounds, such as phenolic acids, flavonoids and polysaccharides [4], which exhibit antimicrobial, antioxidant, anticancer, antityrosinase, and inflammatory properties [5, 6]. However, tree size, alternate bearing, and the witches' broom disease are still serious problem in longan production [1]. And conventional breeding of longan is often hindered by its long juvenility, genetic heterozygosity, and lack of knowledge of genetic background, resulting in a major bottleneck in longan breeding improvement [1].

Recently, more and more draft genome sequences have become available for fruit trees, such as grape (*Vitis vinifera*) [7], papaya (*Carica papaya*) [8], apple (*Malus domestica*) [9], plum

(*Prunus mume*) [10], orange (*Citrus sinensis*) [11], peach (*Prunus persica*) [12], pear (*Pyrus bretschneideri*) [13], and kiwifruit (*Actinidia chinensis*) [14], etc. However, the subtropical and tropical fruit of the Sapindaceae family, includes longan, lychee (*Litchi chinensis*), and rambutan (*Nephelium lappaceum*), are still lacking draft genome sequences. To accelerate the applications of genomics for improving breeding and utilizing the secondary metabolic products of longan, a fundamental understanding of complete genome sequence is crucial. Here, we report the draft genome sequence of longan cultivar 'Honghezi' ($2n=2x=30$) and the extent of genetic diversity based on whole genome re-sequencing of 13 cultivated *D. longan* accessions. Comparative transcriptome studies combined with genome-wide analysis provide insights into the structure and evolution of the longan genome, the molecular mechanisms of the biosynthesis of polyphenol, and pathogen resistance of longan. Together, these results provide insights into evolution and diversity of the longan genome, and improve the efficiency of longan conventional breeding by integrating biotechnological tools.

Results and Discussion

Genome sequencing and assembly

We selected the *D. longan* 'honghezi' cultivar for genome sequencing. In brief, a total of 316.84 Gb of raw data was generated by Illumina sequencing of 12 genome shotgun libraries with different fragment lengths ranging from 170 bp to 40 kb (Table S2). After stringent filtering and correction steps, a total of 121.68 Gb of high-quality sequence data representing 273.44-fold coverage of the entire genome was obtained (Table S3). Based on K-mer frequency methods [15], the *D. longan* genome was estimated to be 445 Mb with 0.88 % heterozygosity rate (Fig S1, Table S4), supporting that fruit trees have high heterozygosity

due to artificial grafting and asexual reproduction [10]. Using the SOAPdenovo program, all high quality reads were assembled into 51,392 contigs and 17,367 scaffolds (≥200bp) totaling 471.88 Mb without N sequence. This assembly accounted for approximately 106.04 % of the estimated longan genome may be due to the high heterozygosity. Compared with other sequenced fruit trees genomes, the genome size of *D. longan* is bigger than that in papaya [8], orange [11], peach [12], and plum [10], but smaller that in grape[7], apple[9], pear[13], and kiwifruit [14]. In the genome assembly, N50 of contigs and scaffolds has 26.04 kb (longest, 173.29 kb) and 566.63 kb (longest, 6942.32 kb), respectively (Table 1). The GC content of the *D. longan* genome was 33.7 %, which was comparable to those for the genome of pineapple (*Ananas comosus*) (33 %) [16], jujube (*Ziziphus jujuba*) (33.41%) [17] and orange (34.06 %) [11], but lower than those for the genome of kiwifruit (35.2 %) [14], papaya (35.3%) [8], and grapevine (36.2%) [7] (Table 2, Fig S2). Analysis of the GC-depth graph and distribution indicated no contamination of any bacterial contig in the genome assembly, and 99.2% of the assembly was sequenced more than 20× coverage (Fig S3). The quality of the assembly was assessed by aligning scaffolds to a longan transcriptome assembly (SRA050205). Of the 96,251 scaffolds (≥100) reported in longan [18], 97.55 % were identified in the assembly (Table S5), indicating the high quality of assembly. The statistics and comparison of the *D. longan* assembly to other twelve fruits genomes were showed in details in Table 2.

Repetitive elements and gene annotation

Repetitive elements are major components of eukaryotic genomes. A total of 52.87% (261.88Mb) of the assembly was found to be composed of repetitive sequences (Table S5), which was higher than those observed in orange (16.8 %) [11], peach (29.6 %) [12], kiwifruit

(36 %) [14], pineapple (38.3 %) [16], grape (41.4 %) [7], apple (42.4%) [9], and papaya (51.9%) [8], suggesting that substantial repetitive element proliferation in longan should be partially accountable for the expansion of the longan genome; but is lower than that observed in pear (53.1 %) [13], and apple (67.4 %) [9] (Table 2). Among the repetitive sequences, tandem repeats occupied about 7.59 % of the genome, interspersed repeats and transposable elements (TEs) accounted for 39.01%, whereas unknown repeats made up 7.71 % (Table S6, TableS7).

By using a combination of *de novo* prediction, homology-based search, and transcriptome assembly, a total of 39,282 protein-coding genes yielding a set of 31,007 high-quality proteins, with an average gene size of 2,890.50 bp, a mean coding sequence size of 1,110.98 bp, and 4.24 exons per gene, were predicted in longan genome (Table S8). The gene content in longan was close to the number of genes predicted in kiwifruit (39,040) [14], lower than those identified in pear (42,812) [13], and apple (57,386) [9], but higher than those in papaya (24,746) [8], pineapple (27,024) [16], peach (27,852) [12], orange (29,445) [11], and grape (30,434) [7]. Of 39,282 protein-coding genes, 31,704 (80.71 %) coding-genes had TrEMBL homologs, 25,708 (65.44 %) had Swiss-Prot homologs and 25,786 (65.64 %) had InterPro homologs (Table S9). It was worth noting that 87 % (34,174 of 39,282) of predicted genes were supported by transcriptome data, suggesting the accuracy of gene annotation in longan genome. In addition, a total of 454 putative transcription factors which were distributed in 55 families were identified in the genome (Supplemental EXCEL File 1). For non-coding genes, there were 359 micorRNAs (miRNAs), 212 rRNA, 506 tRNAs, 399 small nuclear RNAs (snRNAs) in the genome assembly (Table S10).

Gene family evolution and comparison

Orthologous clustering analysis of the longan genome with 8 other selected plant genomes, Arabidopsis, orange, papaya, grapevine, banana (*Musa acuminata*), peach, kiwifruit, and apple was carried out. Of the 39,282 protein-coding genes in the genome, 33,488 were grouped into 15,327 gene families with 1,110 being longan-unique families and 2.18 genes per family (Table S11), the remaining 5,834 were classed into un-clustered genes. Of 15,327 gene families, 8,409 were longan unique orthologs, 5,764 were multiple-copy orthologs, 4,568 were single-copy orthologs, 14,707 were other orthologs (Fig 1b). Comparative analysis of longan with 8 other selected plant genomes indicated that the numbers of gene families of longan was similar to orange (15,000), and peach (15,326), higher than that in banana (12,519), Arabidopsis (13,406), grape (13,570), kiwifruit (13,702), and papaya (13,763), but lower than that in apple (17,740) (Fig 1b, Table S11). Comparative analysis of longan with papaya, citrus, grape, and peach showed that these five plant species contained a core set of 9,475 gene families in common (Fig 1d).

Expansion or contraction of gene families may provide clues to the evolutionary forces that have shaped plant genomes. Here, CAFÉ [19] was utilized to identify gene families that had potentially undergone expansion or contraction in the longan genome. In total, 386 families (7,839 genes) have expanded, while only 12 (53 genes) families have contracted, accounting for 19.96 % and 0.13 % of the total coding-genes (39,282), respectively (Supplemental EXCEL file 2 and 3). Gene Ontology annotations of these expansion or contraction of gene families were assigned to three main GO categories, including biological process, cellular component, and molecular function categories. Furthermore, almost all of the

expansion or contraction of gene families belonged to the biological process; few contracted genes were classified into cellular component, and molecular function categories (Fig S4a, b). These results thus provided clues to the evolutionary forces that have shaped longan genomes.

Genome evolution

Whole genome duplication (WGD) in most plant species is common and represents an important molecular mechanism that shaped modern plant karyotypes [20]. Characterization and annotation of longan genome provided comprehensive information for us to further investigate the evolutionary history of longan. Single copy nuclear genes from orange, Arabidopsis, cacao (*Theobroma cacao*), poplar (*Populus trichocarpa*), grape, apple, papaya, soybean, peach, kiwifruit, and banana [21] were used for genome-scale phylogenic analysis using the maximum likelihood method. Molecular phylogenetic analysis showed that longan was phylogenetically closest to orange, closer to papaya, *Arabidopsis*, and cacao, far more distant from monocotyledon fruits (banana), and was estimated to have diverged from 69.3 million years ago (Fig 1a, and Figure S5). To access the nature of evolutionary events leading to modern longan genome structures, we analyzed the syntenic relationships between longan and popular. In total, there were 2,106 and 883 syntenic blocks containing 17,901 and 17,447 colinear genes of the longan and popular, respectively (Table S12), which supported conserved colinearity and a close evolutionary relationship in these two plant species. To further analyze the evolutionary divergence and the relative age of duplication of longan and other species, we calculated the distance- transversion rate at fourfold degenerate sites (4DTv) rates (Fig 1c). The 4DTv value peaked at 0.5 for paralog pairs in grape, highlighting the recent WGD in this species. Two peaks 4DTv value at 0.72 and 0.6 for the orthologs between

longan and banana or longan and Arabidopsis supported species divergence, respectively. This result was consistent with the more ancient divergence between monocotyledons and dicotyledons. The orthologs between longan and grape, longan and peach or longan and orange showed 4DTv distances peaks at 0.36, 0.36 and 0.26, respectively, which was consistent with that of Vitaceae or Rosaceae, and more ancient than that of Rutaceae or Sapindaceae. In longan, the analysis showed ancient duplication events (peak at ~0.55) but did not reveal recent WGD. These results provided an important complement to the longan genome to study ancestral forms and arrangements of plant genes [22].

Assessment of genetic diversity in longan germplasm

One of the representative characteristics of longan cultivars is highly heterozygous. However, information on extent of heterozygosity in the whole genome is not well understood [23]. The availability of the longan draft genome provides us comprehensive assessment of the heterozygosity in longan genome. Here, we selected 13 representative cultivated accessions with early-maturing, middle-maturing, late-maturing, multiple-flowering, aborted-seeded, disease-resistant characteristics for whole-genome resequencing (Table S1). A total of 45.77 Gb of raw data was generated by Illumina sequencing. After alignment of the clean reads corresponding to 5.02-to 7.31-fold depth and >78 % coverage to the reference (Table S13), we identified 357,737 single nucleotide polymorphisms (SNPs) (Table S14), and 23,225 small insertions/deletions (indels) (Table S15). The overall polymorphism density was 0.05~0.12 SNPs and 0.004~0.007 indels per 10kb in the genome, which was much lower diversity than that of orange [11]. In addition, it was worth noting that the major variations existed among ‘FY’, ‘MQ’, and ‘SJM’ accessions, whereas the variation within the cultivated

longan, especially ‘LDB’ accessions, was relatively low (Table S14 and S15).

To further investigate the population structure and relationships among the longan accessions, we constructed a neighbor-joining tree (Fig 2a) and carried out a principal component analysis (PCA) (Fig 2b). The neighbor-joining tree, constructed based on all SNPs, indicated that 13 longan accessions were distributed into two subfamilies. The first subfamily only consisted of ‘FY’, which showed the highest variations and the clear separation with other cultivars, may due to its special traits, such as witches' broom disease-resistant, middle-maturity, and canned processing products, further supports its diversity at an overall genomic level. The second subfamily consisted of three clades. The first clade includes ‘JHLY’, ‘WLL’, ‘JYW’, and ‘SN1H’; the second one contains ‘MQ’, ‘SX’, ‘SJM’, ‘SEY’; and the third one consisted of ‘DB’, ‘HHZ’, ‘LDB’ and ‘YTB’. Moreover, PCA showed that a tendency for the samples from China, such as ‘HHZ’, ‘DB’, ‘JYW’, ‘LDB’, ‘WLL’, ‘SN1H’, ‘YTB’, ‘SEY’, ‘JHLY’, and ‘SX’, to be clustered together, while showed a clear separation of ‘FY’ (Quanzhou, China), ‘SJM’ (South-East Asia), and ‘MQ’ (Thailand). These results suggested a relationship among 13 selected longan accessions as per their geographical distribution. Additional analysis of population structure was used the FRAPPE program with K (the number of populations) set from 2 to 7 (Fig 2c). When K=7, new subgroup among 13 longan accessions showed some characteristics, such as various maturity, high yielding, aborted-seeding, disease-resistant variety, and multiple flowering. ‘SJM’ (South-East Asia), and ‘MQ’ (Thailand), contain high variations may due to they have a different origins. The cultivar ‘SX’, and ‘YTB’, are susceptible to disease, contain more variations in resistant genes, such as NBS_LRR, and LRR_RLK, than those disease resistant cultivars (‘FY, ‘SN1H,

‘MQ’, ‘LDB’, and ‘JYW’) (Supplemental EXCEL File 4 and 5). These results provided a measure of the change in genetic diversity and a theoretical estimate of the genetic relationship among the selected longan cultivars.

Transcriptome analysis

To improve the gene annotation and address a series of biological questions, we generated 490,502,822 clean reads of RNA-Seq data from nine tissue types, including root, stem, leaf, flower_bud, flower, young fruit, pericarp, pulp, and seed, and used them for mapping, and annotation of the longan genome sequence. About 53.55 ~79.40 % of the unique sRNA sequences from nine RNA-seq data could be mapped to the genome. RNA-Seq data confirmed a majority of annotated introns, identified thousands of novel alternatively spliced mRNA isoforms, extend gene, SNP and indel, indicative of more functional variation than represented by the gene set alone, and a collection of potentially new and longan-specific gene (Table S16). A comparative transcriptome analysis of differential expression in the gene family at nine different developmental stages (root, stem, leaf, flower_bud, flower, young fruit, pericarp, pulp, and seed) showed that most of significant differentially expressed genes were mainly involved in the biosynthesis of secondary metabolites, and plant- pathogen interaction (Table S16, Fig S6), which was fully consistent with the standpoint of *D. longan* species containing high levels of polyphenolic compounds, and a lot of plant pahtogen resistant genes [24, 25].

The biosynthesis of polyphenols and MYB transcription factor in longan

Polyphenols, the potential antioxidative compounds, are the major category of secondary metabolites in longan leaves, flowers, fruit, and seeds [4]. The phenolic compounds are

primarily derived from the shikimic acid pathway, phenylpropanoid pathway, and flavonoid pathway. To assess changes in polyphenols metabolism during longan vegetative and reproductive growth between primary and secondary metabolism, the copy number of key genes within the shikimate acid pathway, phenylpropanoid pathway, and flavonoid, compared with that from Arabidopsis, orange, peach, and grape, was analyzed (Fig 3a), and their expression was measured (Fig 3b, Supplemental EXCFL File 6 and 7). PCA showed that all the genes related to biosynthesis of polyphenols were expressed at varying levels among 9 different vegetative growth and reproductive growth samples, with some preferentially expressed in specific tissues, such as flower and flower bud (Fig 3b).

The copy numbers of *DHS*, *DHQS*, *SK*, *EPSP*, *CS*, *CM*, and *ADT* genes, involved in shikimic acid pathway, with few variations among longan, *Arabidopsis*, orange, peach, and grape, indicated their evolutionary conservation in different plant species. However, SDH, catalyzes the NADPH-dependent reduction of 3- dehydroshikimate to shikimate in the fourth step of the shikimate pathway, is a metabolic route required for the biosynthesis of the aromatic amino acids, had six copy numbers in longan, much higher than those in *Arabidopsis* (1 copy), orange (3 copy), peach (2 copy), and grape (2 copy). The FPKM analysis among 9 tissues showed that two members of *SDH* family, Cs9g05070.1-D1, and Cs9g05070.1-D5, showed highly levels during vegetable and reproductive stages, especially in pulp and pericarp, while the other members were barely detectable, suggesting that Cs9g05070.1-D1, and Cs9g05070.1-D5 may play major roles in the skikimate acid pathway.

Few variations of the copy numbers of *PAL*, *C4H*, *4CL*, *CHS*, *CHI*, *F3H*, *F3'5'H*, *DFR*, *ANS*, and *UFGT* genes, involved in phenylpropanoid, and flavonoid pathway, were also observed in

the selected plants, but *F3'H*, *ANR*, and *LAR*, had higher copy numbers in longan than that of Arabidopsis, orange, and grape. Especially, F3'H, which is involved in flavonoid biosynthesis, are important for flower color and fruit skin. Thirty five members for *F3'H* gene were found in longan genome (Fig.3c), exhibiting different temporal and spatial expression levels during vegetable and reproductive tissues (Fig.3d). Among these members, the highest expression levels were observed for one in root, two in stem, five in leaf, eleven in flower bud, three in flower, six in young fruit, three in pericarp, and three in seeds; while eleven members were barely detectable in pericarp, pulp, and seeds, further implying that *F3'H* played major roles in determining longan flower colors.

Proanthocyanidins (PA, also called condensed tannins), are crucial polyphenolic compounds for *D. longan*. PA synthesis involves both leucoanthocyanidin reductase (LAR) and anthocyanidin reductase (ANR) (Fig.3c). In longan, the ANR enzyme was encoded by six genes whereas LAR was encoded by four genes. The six *ANR* genes and two of four *LAR* genes were barely detectable in the pulp, but all of the *ANR* or *LAR* genes were verified and strongly expressed in pericarp, and less expressed in seeds (Fig.3d). Previous studies showed the most abundances of the total polyphenols, tannins and proanthocyanidin of 12 varieties of Chinese longan fruits in pericarp followed by seed, and pulp [26]. High expression levels of *ANR* and *LAR* in pericarp and seed, and the lowest expression levels of them in purp indicated they were responsible for determining tannin composition of the longan fruit, further giving an interpretation that why the whole longan fruit eaten dried for use in sweet dessert soups for human health [27].

The MYB family of proteins are verified involving in the regulation of flavonoid biosynthesis

[28]. To further understanding the biosynthesis of polyphenols in longan, the numbers of MYB transcription factor compared with that of *Arabidopsis*, orange, peach, and grape; and their expression was investigated in longan by using genome and transcriptome data. In total, 94 *R2R3-MYB* genes were detected, the numbers were more than those of orange (74), and peach (88), but less than that of grape (116), and Arabidopsis (141) (Fig 4a, b). PCA showed that all the MYB family members were expressed at varying levels among 9 different vegetative growth and reproductive growth samples, with some preferentially expressed in specific tissues (Fig 4c, Supplemental EXCEL File 8). Specific *R2R3-MYB* family members, such as *AtMYB*3~-5, -7, -11, -12, -32, -75, -90, -111, -113, -114, and -123, are verified to be involved in the regulation of the flavonoid pathway [28]. In longan, only 4 *R2R3-MYB* genes homogenous of *AtMYB*4, -12, and -123 were found. In *Arabidopsis*, *AtMYB4* was down-regulated *C4H*, *-12* was up-regulated *CHS*, *CHI*, *F3H*, and *F3'H*, and *-123* was up-regulated *DNS* [28]. All of the 4 *R2R3-MYB* genes homogenous reached peaks in root, but undetected in pulps (Fig 4d), their tissue specific expression indicating that of these genes might be required for the flavonoid biosynthesis.

Identification and classification of NBS-LRR and LRR-RLK encoding genes

Transcriptome data showed that longan contained a lot of plant pahtogen resistant genes. To further investigate the molecular basis for longan pathogen susceptibility, we searched for two classes of resistance genes consisting of nucleotide binding site-leucine rich repeat (NBS- LRR) proteins and leucine rich repeat- receptor-like kinases (LRR-RLK) in the longan genome. A total of 594 NBS-LRR and 338 LRR-RLK encoding genes were identified, and accounted for approximately 1.51 %

and 0.86 % of longan protein-coding genes, respectively. Furthermore, the numbers of NBS-LRR and LRR-RLK genes in longan genome were more than those in orange (509, 325), grape (341, 234), kiwifruit (110, 259), peach (425, 268), and papaya (60, 134), but nearly half that of apple (1035, 477) (Table S17). The NBS-LRR- and LRR-RLK- encoding genes contracted tremendously in the longan, orange, and papaya genomes compared with apple, suggesting that contraction in the numbers of NBS-LRR- and LRR-RLK- encoding genes in different species may have altered their resistance to disease after their divergence from apple.

Of these identified disease resistance genes, 594 NBS-LRR genes were classified into six subgroups based on protein domains and sequence conservation (Table S17). Among them, NBS-LRR (NL) (258, 43.43 %) were the most highly represented, followed by coiled-coil (CC) -NBS-LRR- (CNL) (150, 25.25 %), NBS(N) (122, 20.54 %), and CC-NBS (CN)(37, 6.23 %). Only a few were assigned into Toll interleukin receptor (TIR) -NBS-LRR (TNL) (23, 3.87 %), TIR-NBS (TN) (4, 0.67%). The most highly represented NL subgroup was also found in orange, cacao, grape, kiwifruit, and peach genomes, while the N subgroup genes were the most highly represented in papaya and apple genome. The numbers of CC-motif- encoding genes (187 in total) were much more than that of TIR-motif encoding genes (27) in longan genome, which were similar to those observed in orange, cacao, grape, and kiwifruit, but in contrast to those found in apple and peach. The identified resistance genes would assist in the further study of their functions in longan.

Conclusion

We have presented a draft genome of *D. longan* for the first time. The draft longan genome sequence was assembled into a 471.88 Mb genome representing 273.44-fold coverage by paired-end sequencing. Whole-genome resequencing and analysis of 13 representative cultivated *D. longan* accessions identified the extent of genetic diversity, and trait discovery. Characterization of the longan genome for protein-coding genes, comparative genomic analysis, and transcriptome analyses, provided insights into longan specific traits, particularly those involved in the biosynthesis of secondary metabolites and pathogen resistance.

Methods

Germplasm genetic resources

An 80-year old of *D. longan* 'Honghezi' from Fujian Agriculture and Forestry University, China, was used for genomic DNA isolation and sequencing. RNA samples from the roots, leaves, floral buds, flowers, young fruits, mature fruits, pericarp, pulp and seeds of another cultivar *D.longan* 'Sijimi' from the experimental fields of Fujian Academy of Agricultural Science in Putian, Fujian Province, were collected for transcriptome sequencing. Fourteen *D. longan* cultivars, 'Honghezi'(HHZ), 'Sijimi'(SJM), 'Shuinanyihao'(SN1H), 'Jiuyuewu' (JYW), 'Shixia'(SX), 'Wulongling'(WLL), 'Miaoqiao'(MQ), 'Youtanben'(YTB), 'Shieryue' (SEY), 'Lidongben'(LDB), 'Jiaohelongyan'(JHLY), 'Fuyan'(FY), 'Dongbi'(DB), and 'Songfengben'(SFB), originated or were popularized in different Asian and international regions, were collected for resequencing.

DNA extraction, library construction, whole-genome shotgun sequencing and assembly

WGS sequencing was performed with the Illumina HiSeq 2000 System. Genomic DNA was

extracted from fresh mature leaves of *D. longan* cultivar using the modified SDS method. DNA sequencing libraries were constructed using standard Illumina libraries prep protocols. A total of 12 paired-end sequencing libraries, spanning sizes of 170, 250, 500, 800, 2,000, 5,000, 10,000, 20,000, and 40,000 bp, were constructed and sequenced with an Illumina HiSeq 2000 system. After stringent filtering and correction steps by K-mer frequency-based methods [15], a total of 121.68 Gb data were obtained, and then assembled using SOAP *de novo* and SSPACE software [29]. To check the completeness of the assembly, we mapped a longan transcriptome assembly comprising 68,925 unigenes (SRA050205), to the genome assembly using BLAT32 at various sequence homology and coverage parameters.

Repetitive elements identification

Tandem repeats and interspersed repeats are two main types of repeats in the genome. Tandem repeats were first identified using LTR_FINDER with default parameters. Interpersed repeats were identified by Repeat Masker (http://www.repeatmasker.org/) and RepeatProteinMask using a Repbase library [30] and the de novo transposable element library. Identified repeats were then classified into different known classes as previously described [22].

Gene prediction and annotation

For gene predictions, three main approaches of *de novo* prediction, homology-based method, and RNA-sequenced unigenes- based method were used. For *de novo* prediction, Augustus[31], GENSCAN [32] and GlimmerHMM [33] were used with parameters trained on *A.thaliana* and *C.papaya*, then these *de novo* predictions were merged into a unigene set. For the homology search, protein sequences from three sequenced plants (*Glycine max*,

Populus trichocarpa, and *Vitis vinifera*) were mapped to the longan genome assembly using TBLASTN at an E value cutoff of 1×10^{-5}, respectively. To further extract accurate exon–intron information, the homologous genome sequences were then aligned against the matching proteins using GeneWise [34]. Subsequently, Illumina RNA-seq unigenes [18] were aligned to the genome assembly using BLAT[35] to derive spliced alignments.

To finalize the consensus gene set, results of these three methods described above were integrated using GLEAN program [36], and we got the final gene set that contains 39,282 genes. Non-coding RNA were predicted and classified as previously described [37]. Functions of the predicted protein genes were searched with BLAST (E value cutoff of 1×10^{-5}) against as InterproScan [38], GO [39], KEGG [40], Swissprot [41], and TrEMBL databases.

Gene families and phylogenetic analysis

Proteins sequences from *T. cacao*, *C. sinensis*, *A. thaliana*, *C. papaya*, *O. sativa*, *P. trichocarpa*, *G. max*, *C. sativus*, *V. vinifera*, *M. acuminata*, *G. raimondii* were used to identify gene families using BLASTP (E-value: 1e-5), and gene family clusters among different plant species were identified by OrthoMCL [42]. Single-copy families with representation in all species were selected to perform alignment by MUSCLE [43]. Fourfold degenerate sites (4D) were used to construct a phylogenetic tree using twelve species by MRBAYES [44], and the divergence time was estimated using the software MultiDivtime [43]. The colinearity analysis between *D. longan* and *P. trichocarpa* were computed by SyMAP v3.4[45]. Subsequently, transcription factor families were identified using the IPR2genomes tool in GreenphylDB v2.0 [46] based on InterPro domains, and gene family expansion and contraction within

phylogenetically related organisms were detected by the computational analysis of gene family evolution computer program (CAFÉ) [19].

Resequencing, SNPs, Indels and SVs analysis

Paired-end Illumina libraries for thirteen *D. longan* cultivars were prepared following the manufacturer's instructions and sequenced by an Illumina HiSeq 2000 System. After stringent filtering and correction steps, the resulting sequence data was uniquely aligned to the reference genome. SNPs, InDels, and SVs were then identified on the basis of SOAPsnp (http://soap. genomics.org.cn/ soapsnp.html), SOAPindel [47], and SOAPsv [48].

We used all and high quality SNPs to infer phylogeography and population structure for *D. longan*. For phylogeny, a tree was subsequently generated using the neighbour-joining method implemented in TreeBeST, bootstrap was seted 1000 replicates.

Population structure was examined primarily via PCA by our own program and model-based clustering algorithms implemented in FRAPPE v1.1 (http:// smstaging.stanford.edu/tanglab/ software/frappe.html), We increased the pre-defined genetic clusters from K2 to K7 and ran analysis with 10,000 maximum iterations.

Transcriptome sequencing

Transcriptome sequencing was performed with the Illumina HiSeq 2000 System. Total RNAs from the samples descried above were isolated using the TRIzol Reagent kit (Invitrogen, Carlsbad, CA). cDNA libraries were constructed and sequenced using Illumina protocols. All raw reads were first processed to remove the adaptor sequences, law quality and possible contaminations from chloroplast, mitochondrion and ribosomal DNA. Clean reads were then aligned to the longan genome sequences using TopHat [49] to identify exons and splice

junctions ab initio. For matched genes, their expression levels in each cDNA library was derived and normalized to FPKM. The software Cluster 3.0 [50] was performed to analyze hierarchical clustering on genes. Differentially expressed genes among different samples were identified using the EBSeq packages[51]. Subsequently, GATK (http://www.broadinstitute.org/gatk/) with default parameters was used to call SNPs based on transcript sequence data.

Identification of genes in secondary metabolites

We downloaded all the proteins of pathway from from Arabidopsis, orange, peach, and grape; and these genes for each species were identified using the following methods. At first, we collected previously published related genome sequences as query sequences. Then, TBLASTN (Legacy Blast v2.2.23) [35] aligned against each genome sequence with a threshold of e-value <1e-10. As obtained so many TBLASTN results that hit the same genomic region. We extracted high quality alignments (Query_align_ratio ≥ 70% and Identity ≥ 40%). Functional intact genes were confirmed via the following approach. Firstly, we collected the blast-hits using the above method. Then, each of the blast-hits sequences were extended in both 3' and 5' directions along the genome sequences in order to predict gene structure by Genewise (v2.2.0)[34]. Finally, we got all the pathway genes in longan and other fruit.

Identification of *MYB* genes

We download the *MYB* genes from Arabidopsis, orange, peach, and grape, and the identification methods is similar in the part of ‘Identification of genes in secondary metabolites’. The *MYB* genes in longan and other fruits were shown in Figure 4, and

their expression in 9 longan samples were shown in Supplementary EXCEL file 8.

Disease resistance genes analysis

Identification of longan resistance-related genes was based on the most conserved motif structures of plant resistance proteins. The detail methods were described as in [9]. The distribution of longan genes encoded domains similar to plant R proteins and comparison with other sequenced genomes was showed in Table S17.

Availability of data and material

The whole-genome sequences of the *D. longan* project have been deposited at NCBI database under BioProject PRJNA305337. The NCBI SRA database with accession numbers SRA315202, and the sample Accession were SRS1272137, SRS1272138, SRS1272139, and SRS1272140. Sequencing data, annotations and analyses results have all been uploaded to the FTP site ftp://ftp.genomics.org.cn/from _ BGISZ/ 20130120/for evaluation. *D.longan* ‘SIJIMI’ transcriptome data deposited at NCBI database under BioProject PRJNA326792.

Abbreviations

Mb: million base **TEs**: transposable elements **miRNAs:** micorRNAs **sn**RNAs: small nuclear RNAs **WGD:** Whole genome duplication **SNPs:** single nucleotide polymorphisms **indels:** insertions/deletions **PCA:** principal component analysis **DHS:** 3-deoxy-D-arabino-heptulosonate 7-phosphate synthase **DHQS:** 3-dehydroquinate synthase **SDH:** Bifunctional 3- dehydroquinate dehydratase/ shikimate dehydrogenase; **SK:** Shikimate kinase **EPSPS:** 3- phosphoshikimate 1- carboxyvinyltransferase/5-

enolpyruvylshikimate-3-phosphate/EPSP synthase **CS:** chorismate synthase **CM:** chorismate mutase **ADT:** arogenate dehydratase/ prephenate dehydratase **PAL:** phenylalanine ammonia lyase **C4H:** cinnamate 4-hydroxylase **4CL:** 4-coumaroyl-coenzyme A ligase **CHS:** chalcone synthase **CHI:** chalcone-flavanone isomerase **F3H:** flavanone 3-hydroxylase **F3'H:** flavonoid 3'-hydroxylase **F3'5'H:** flavonoid 3',5'-hydroxylase **ANS:** anthocyanidin synthase **LDOX:** leucoanthocyanidin dioxygenase **DFR:** dihydroflavonol 4-reductase **LAR:** leucoanthocyanidin reductase.

Declarations

Additional files

Supplementary EXCEL file 1-*D.longan* tanscription factor identification

Supplementary EXCEL file 2 *D. longan* gene family.decrease

Supplementary EXCEL file 3 *D. longan* gene family-expansion

Supplementary EXCEL file 4 FY, SN1H, MQ, LDB, and JYW SNP analysis-r1.filter.anno

Supplementary EXCEL file 5 SX, and YTB SNP analysi -r2.filter.anno

Supplementary EXCEL file 6 Statistics of genes copy numbers of biosynthesis of polyphenols in different plants

Supplementary EXCEL file 7 Genes expression levels of biosynthesis of polyphenols in longan

Supplementary EXCEL file 8 longan MYB genes expressed in 9 different samples

The authors declare no competing financial interests.

Consent for publication

Not applicable

COMPETING FINANCIAL INTERESTS

The authors declare no competing financial interests.

Funding

This work was funded by Research Funds for the National Natural Science Foundation of China (31572088, 31272149, 31201614, and 31078717), Science and Technology Plan Major Projects of Fujian Province (2015NZ0002), the Natural Science Funds for Distinguished Young Scholar in Fujian Province (2015J06004), program for New Century Excellent Talents in Fujian Province University (20151104), the Doctoral Program of Higher Education of the Chinese Ministry of Education (20093515110005 and 20123515120008), the Education Department of Fujian Province Science and Technology Project (JA14099), Program for High- level university construction of FAFU (612014028), and the Natural Science Funds for Distinguished Young Scholar of FAFU (xjq201405).

Authors’ contributions

ZXL, YLL, YY, and RKV designed research; YLL, ZXL, RLL, YKC, CZC, QLT, WHL, L XL, DMZ, MKT, ZHZ, CSZ, and SCL collected samples and prepared DNA and RNA. LLY, ZYW, QFL, and YH sequenced, processed the raw data, sequence assembly. XDF, ZYW, CGZ, JW, and MHY coordinated the project; JMM, LLY, ZYW, QFL, YH, and YLL analyzed data; YLL, ZXL, YY, JMM, and RKV wrote and revised the paper.

Acknowledgments

We thank the following colleagues from the experimental fields of the Fujian Academy of Agricultural Science in Putian for samples.

References

1. Lai Z, Chen C, Zeng L, Chen Z: Somatic embryogenesis in longan [*Dimocarpus longan* Lour.]. In: *Somatic Embryogenesis in Woody Plants.* Edited by Jain SM, Gupta P, Newton R, vol. 67: Springer Netherlands; 2000: 415-431.
2. Luo J, Zhou C-f, Wan Z: Analysis on the Development Status of Lychee Industry in Guangdong Province in 2010. *Guangdong Agricultural Sciences* 2011, 4:16-18.
3. Mei ZQ, Fu SY, Yu HQ, Yang LQ, Duan CG, Liu XY, Gong S, Fu JJ: Genetic characterization and authentication of *Dimocarpus longan* Lour. using an improved RAPD technique. *Genet Mol Res* 2014, 13(1):1447-1455.
4. Jiang G, Jiang Y, Yang B, Yu C, Tsao R, Zhang H, Chen F: Structural characteristics and antioxidant activities of oligosaccharides from longan fruit pericarp. *Journal of agricultural and food chemistry* 2009, 57(19):9293-9298.
5. Chung YC, Lin CC, Chou CC, Hsu CP: The effect of Longan seed polyphenols on colorectal carcinoma cells. *European journal of clinical investigation* 2010, 40(8):713-721.
6. Prasad KN, Yang B, Shi J, Yu C, Zhao M, Xue S, Jiang Y: Enhanced antioxidant and antityrosinase activities of longan fruit pericarp by ultra-high-pressure-assisted extraction. *Journal of pharmaceutical and biomedical analysis* 2010, 51(2):471-477.
7. Jaillon O, Aury JM, Noel B, Policriti A, Clepet C, Casagrande A, Choisne N, Aubourg S, Vitulo N, Jubin C *et al*: The grapevine genome sequence suggests ancestral hexaploidization in major angiosperm phyla. *Nature* 2007, 449(7161):463-467.
8. Ming R, Hou S, Feng Y, Yu Q, Dionne-Laporte A, Saw JH, Senin P, Wang W, Ly BV, Lewis KL *et al*: The draft genome of the transgenic tropical fruit tree papaya (*Carica papaya* Linnaeus). *Nature* 2008, 452(7190):991-996.
9. Velasco R, Zharkikh A, Affourtit J, Dhingra A, Cestaro A, Kalyanaraman A, Fontana P, Bhatnagar SK, Troggio M, Pruss D *et al*: The genome of the domesticated apple (*Malus* x *domestica* Borkh.). *Nature genetics* 2010, 42(10):833-839.
10. Zhang Q, Chen W, Sun L, Zhao F, Huang B, Yang W, Tao Y, Wang J, Yuan Z, Fan G *et al*: The genome of Prunus mume. *Nature communications* 2012, 3:1318.
11. Xu Q, Chen LL, Ruan X, Chen D, Zhu A, Chen C, Bertrand D, Jiao WB, Hao

BH, Lyon MP *et al*: The draft genome of sweet orange (*Citrus sinensis*). *Nature genetics* 2013, 45(1):59-66.

12. Verde I, Abbott AG, Scalabrin S, Jung S, Shu S, Marroni F, Zhebentyayeva T, Dettori MT, Grimwood J, Cattonaro F *et al*: The high-quality draft genome of peach (*Prunus persica*) identifies unique patterns of genetic diversity, domestication and genome evolution. *Nature genetics* 2013, 45(5):487-494.
13. Wu J, Wang Z, Shi Z, Zhang S, Ming R, Zhu S, Khan MA, Tao S, Korban SS, Wang H *et al*: The genome of the pear (Pyrus bretschneideri Rehd.). *Genome Res* 2013, 23(2):396-408.
14. Huang S, Ding J, Deng D, Tang W, Sun H, Liu D, Zhang L, Niu X, Zhang X, Meng M *et al*: Draft genome of the kiwifruit *Actinidia chinensis*. *Nature communications* 2013, 4:2640.
15. Li R, Fan W, Tian G, Zhu H, He L, Cai J, Huang Q, Cai Q, Li B, Bai Y *et al*: The sequence and de novo assembly of the giant panda genome. *Nature* 2010, 463(7279):311-317.
16. Ming R, VanBuren R, Wai CM, Tang H, Schatz MC, Bowers JE, Lyons E, Wang M-L, Chen J, Biggers E *et al*: The pineapple genome and the evolution of CAM photosynthesis. *Nature genetics* 2015, advance online publication.
17. Ma Q, Feng K, Yang W, Chen Y, Yu F, Yin T: Identification and characterization of nucleotide variations in the genome of Ziziphus jujuba (Rhamnaceae) by next generation sequencing. *Mol Biol Rep* 2014, 41(5):3219-3223.
18. Lai Z, Lin Y: Analysis of the global transcriptome of longan (*Dimocarpus longan* Lour.) embryogenic callus using Illumina paired-end sequencing. *BMC Genomics* 2013, 14:561.
19. De Bie T, Cristianini N, Demuth JP, Hahn MW: CAFE: a computational tool for the study of gene family evolution. *Bioinformatics* 2006, 22(10):1269-1271.
20. Guo S, Zhang J, Sun H, Salse J, Lucas WJ, Zhang H, Zheng Y, Mao L, Ren Y, Wang Z *et al*: The draft genome of watermelon (*Citrullus lanatus*) and resequencing of 20 diverse accessions. *Nature genetics* 2013, 45(1):51-58.
21. D'Hont A, Denoeud F, Aury JM, Baurens FC, Carreel F, Garsmeur O, Noel B, Bocs S, Droc G, Rouard M *et al*: The banana (*Musa acuminata*) genome and the evolution of monocotyledonous plants. *Nature* 2012, 488(7410):213-217.
22. Huang S, Li R, Zhang Z, Li L, Gu X, Fan W, Lucas WJ, Wang X, Xie B, Ni P *et al*: The genome of the cucumber, *Cucumis sativus* L. *Nature genetics* 2009, 41(12):1275-1281.
23. Lin T, Lin Y, Ishiki K: Genetic diversity of Dimocarpus longan in China revealed by AFLP markers and partial rbcL gene sequences. *Scientia Horticulturae* 2005, 103(4):489-498.
24. Lin Y, Lai Z: Comparative analysis reveals dynamic changes in miRNAs and their targets and expression during somatic embryogenesis in longan (*Dimocarpus longan* Lour.). *PLoS One* 2013, 8(4):e60337.
25. Lin CC, Chung YC, Hsu CP: Potential roles of longan flower and seed extracts

for anti-cancer. *World journal of experimental medicine* 2012, 2(4):78-85.

26. He N, Wang Z, Yang C, Lu Y, Sun D, Wang Y, Shao W, Li Q: Isolation and identification of polyphenolic compounds in longan pericarp. *Separation and Purification Technology* 2009, 70(2):219-224.
27. Tseng HC, Wu WT, Huang HS, Wu MC: Antimicrobial activities of various fractions of longan (*Dimocarpus longan* Lour. Fen Ke) seed extract. *International journal of food sciences and nutrition* 2014.
28. Dubos C, Stracke R, Grotewold E, Weisshaar B, Martin C, Lepiniec L: MYB transcription factors in Arabidopsis. *Trends in plant science* 2010, 15(10):573-581.
29. Boetzer M, Henkel CV, Jansen HJ, Butler D, Pirovano W: Scaffolding pre-assembled contigs using SSPACE. *Bioinformatics* 2011, 27(4):578-579.
30. Jurka J, Kapitonov VV, Pavlicek A, Klonowski P, Kohany O, Walichiewicz J: Repbase Update, a database of eukaryotic repetitive elements. *Cytogenetic and genome research* 2005, 110(1-4):462-467.
31. Stanke M, Keller O, Gunduz I, Hayes A, Waack S, Morgenstern B: AUGUSTUS: ab initio prediction of alternative transcripts. *Nucleic acids research* 2006, 34(Web Server issue):W435-439.
32. Salamov AA, Solovyev VV: Ab initio gene finding in *Drosophila* genomic DNA. *Genome Res* 2000, 10(4):516-522.
33. Majoros WH, Pertea M, Salzberg SL: TigrScan and GlimmerHMM: two open source ab initio eukaryotic gene-finders. *Bioinformatics* 2004, 20(16):2878-2879.
34. Birney E, Clamp M, Durbin R: GeneWise and Genomewise. *Genome Res* 2004, 14(5):988-995.
35. Kent WJ: BLAT--the BLAST-like alignment tool. *Genome Res* 2002, 12(4):656-664.
36. Elsik CG, Mackey AJ, Reese JT, Milshina NV, Roos DS, Weinstock GM: Creating a honey bee consensus gene set. *Genome Biol* 2007, 8(1):R13.
37. Varshney RK, Song C, Saxena RK, Azam S, Yu S, Sharpe AG, Cannon S, Baek J, Rosen BD, Tar'an B *et al*: Draft genome sequence of chickpea (Cicer arietinum) provides a resource for trait improvement. *Nature biotechnology* 2013, 31(3):240-246.
38. Zdobnov EM, Apweiler R: InterProScan--an integration platform for the signature-recognition methods in InterPro. *Bioinformatics* 2001, 17(9):847-848.
39. Ashburner M, Ball CA, Blake JA, Botstein D, Butler H, Cherry JM, Davis AP, Dolinski K, Dwight SS, Eppig JT *et al*: Gene ontology: tool for the unification of biology. The Gene Ontology Consortium. *Nature genetics* 2000, 25(1):25-29.
40. Kanehisa M, Goto S: KEGG: kyoto encyclopedia of genes and genomes. *Nucleic acids research* 2000, 28(1):27-30.
41. Bairoch A, Apweiler R: The SWISS-PROT protein sequence database and its supplement TrEMBL in 2000. *Nucleic acids research* 2000, 28(1):45-48.

42. Li L, Stoeckert CJ, Jr., Roos DS: OrthoMCL: identification of ortholog groups for eukaryotic genomes. *Genome Res* 2003, 13(9):2178-2189.

43. Edgar RC: MUSCLE: multiple sequence alignment with high accuracy and high throughput. *Nucleic acids research* 2004, 32(5):1792-1797.

44. Huelsenbeck JP, Ronquist F: MRBAYES: Bayesian inference of phylogenetic trees. *Bioinformatics* 2001, 17(8):754-755.

45. Soderlund C, Bomhoff M, Nelson WM: SyMAP v3.4: a turnkey synteny system with application to plant genomes. *Nucleic acids research* 2011, 39(10):e68.

46. Rouard M, Guignon V, Aluome C, Laporte MA, Droc G, Walde C, Zmasek CM, Perin C, Conte MG: GreenPhylDB v2.0: comparative and functional genomics in plants. *Nucleic acids research* 2011, 39(Database issue):D1095-1102.

47. Li S, Li R, Li H, Lu J, Li Y, Bolund L, Schierup MH, Wang J: SOAPindel: efficient identification of indels from short paired reads. *Genome Res* 2013, 23(1):195-200.

48. Li Y, Zheng H, Luo R, Wu H, Zhu H, Li R, Cao H, Wu B, Huang S, Shao H *et al*: Structural variation in two human genomes mapped at single-nucleotide resolution by whole genome de novo assembly. *Nature biotechnology* 2011, 29(8):723-730.

49. Trapnell C, Pachter L, Salzberg SL: TopHat: discovering splice junctions with RNA-Seq. *Bioinformatics* 2009, 25(9):1105-1111.

50. de Hoon MJ, Imoto S, Nolan J, Miyano S: Open source clustering software. *Bioinformatics* 2004, 20(9):1453-1454.

51. Leng N, Dawson JA, Thomson JA, Ruotti V, Rissman AI, Smits BM, Haag JD, Gould MN, Stewart RM, Kendziorski C: EBSeq: an empirical Bayes hierarchical model for inference in RNA-seq experiments. *Bioinformatics* 2013, 29(8):1035-1043.

Table Click here to download Table Tables.doc

Tables

Table 1 *D. longan* genome assembly

	Contig		Scaffold	
	Size(bp)	Number	Size(bp)	Number
N90	6,457	18,861	122,626	983
N80	11,286	13,434	197,247	668
N70	15,938	9,933	283,489	459
N60	20,685	7,339	396,999	309
N50	26,035	5,306	566,629	204
Longest	173,288		6,942,318	
Total size	471,874,380		495,332,425	
Total number(>=200bp)		51,392		17,367
Total number(>=2Kb)		27,296		2,282

Table 2 Statistics and comparison of the *D. longan* assembly to other twelve genomes. Dl, *Dimocarpus longan;* Cs, *Citrus sinensis;* Cc, *Citrus Clementina;* Cp, *Carica papaya;* Ac, *Actinidia chinensis;* Md, *Malus domestica;* Pp, *Prunus persica*; Pb, *Pyrus bretschneideri*; Vv, *Vitis vinifera;* Ac, *Ananas comosus (L.) Merr.*; Zj, *Ziziphus jujuba Mill.*; Mn, *Morus notabilis;* Tc, *Theobroma cacao*.

	Dl	Cs	Cc	Cp	Ac	Md	Pp	Pb	Vv	Ac	Zj	Mn	Tc
Chromosome number (2*n*)	30	18	18	18	58	34	16	34	38	50	24	14	20
Estimate of genome size (Mb)	445	367	370	372	758	742.3	265	527	475	526	444	357	430
Sequence Coverage	273.43	214	7	NA	140	16.9	8.47	194	8.4	400	390	236	16.7
Assembled (Mb)	471.88	320	301	271	616.1	603.9	226.6	512	487	382	437.65	330	326.9
Assembling represent percentage of genome (%)	106.4	87.30	81.4	75	81	81.3	85.50	97.10	102.5	73	98.60	92.4	76
N50 length of contig (Kb)	26.03	49.89	NA	NA	58.9	16.17	294	35.7	65.9	126.5	33.9	34.4	19.8
N50 length of scaffolds (Mb)	0.56662	1.69	NA	NA	0.646	NA	4	0.54	2	11.8	0.3	0.39	0.4738
GC content (%)	33.7	34.06	NA	35.3	35.20	NA	NA	NA	35	33	33.41	35	NA
Repeat content (%)	52.87	20	NA	51.90	36	67.4	29.60	53.10	41.40	38.30	49.49	38.8	25.70
Number of gene models	39,282	29,445	24,533	24,746	39,040	57,386	27,852	42,812	30,434	27,024	32,808	27,085	28,798

NA, no available.

Figure 1 Phylogenetic and evolutionary analysis of the longan genome
Click here to download Figure Figure 1 Phylogenetic and evolutionary analysis of the longan genome.doc

Figure 1 Phylogenetic and evolutionary analysis of the longan genome. (**a**) Molecular phylogenetic analysis based on single-copy genes shared among orange, papaya, Arabidopsis, cacao, poplar, banana, grape, soybean, apple, peach, kiwifruit, and banana from genome data. (**b**) Comparison of the number of gene families in eleven plant species, such as *T. cacao*, *A. thaliana*, *C. sinensis*, *C. papaya*, *P. trichocarpa* , *G. max*, *V. vinifera*, *M. acuminate*, *D. longan*, *P. persica*, *A. chinensis*, and *M. domestica.* (**c**) Distribution of 4DTv distance between syntenic gene pairs among banana, peach, orange, Arabidopsis and grape. (**d**) Distribution of gene families among *D. longan*, *C. sinensis*, *C. papaya*, *V. vinifera*, and *P. persica*. Homologous genes in longan, orange, papaya, grape, and peach were clustered to gene families. The numbers of gene families are indicated for each species and species intersection.

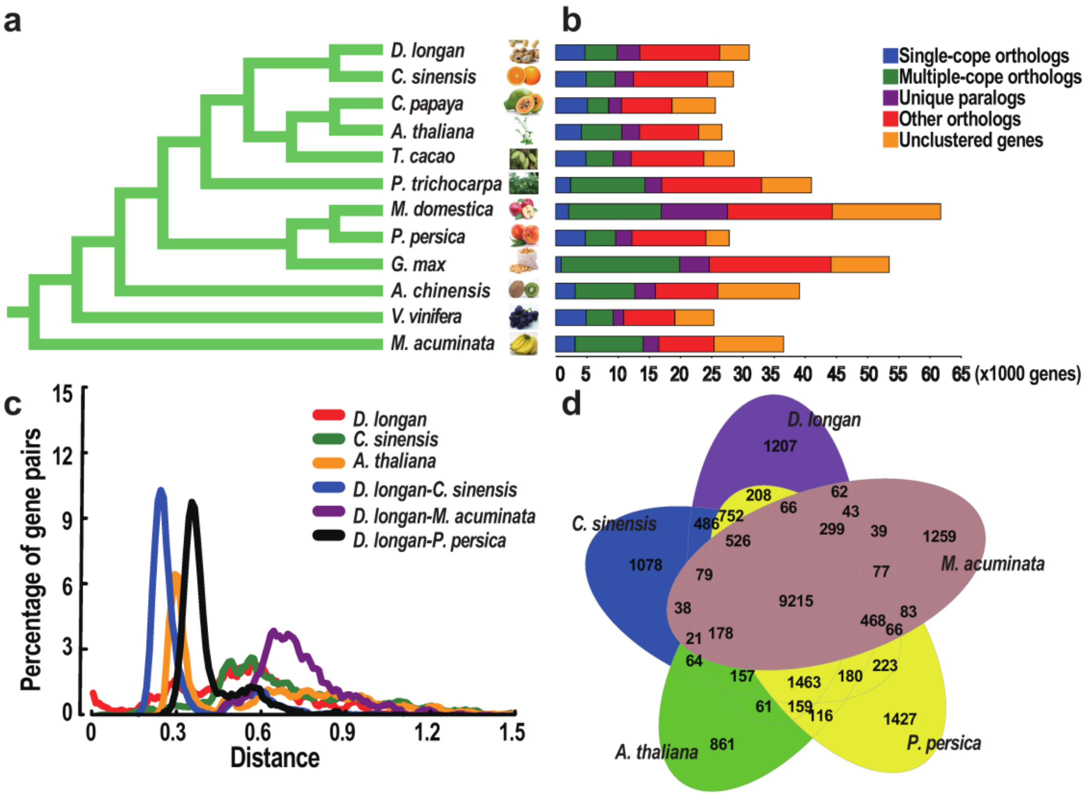

 Click here to download Figure Figure 2 Genetic diversity and population structure of longan accessions..doc

Figure 2 Genetic diversity and population structure of longan accessions. (**a**) Neighbor- joining tree of the 13 longan accessions on the basis of all SNPs. (**b**) PCA of the 13 longan accessions using SNPs as markers. Different colors represent for different longan accession. HHZ, DB, JYW, LDB, WLL, SN1H, YTB, SEY, JHLY, and SX, are clustered together, FY (Quanzhou, China), SJM (South-East Asia), and MQ (Thailand) showed a clear separation. (**c**) Population structure of longan accessions. The distribution of the accessions to different populations is indicated by different color. Each accession is represented by a vertical bar. Numbers on the x-axis show represents the K number, and the y-axis shows the different accession.

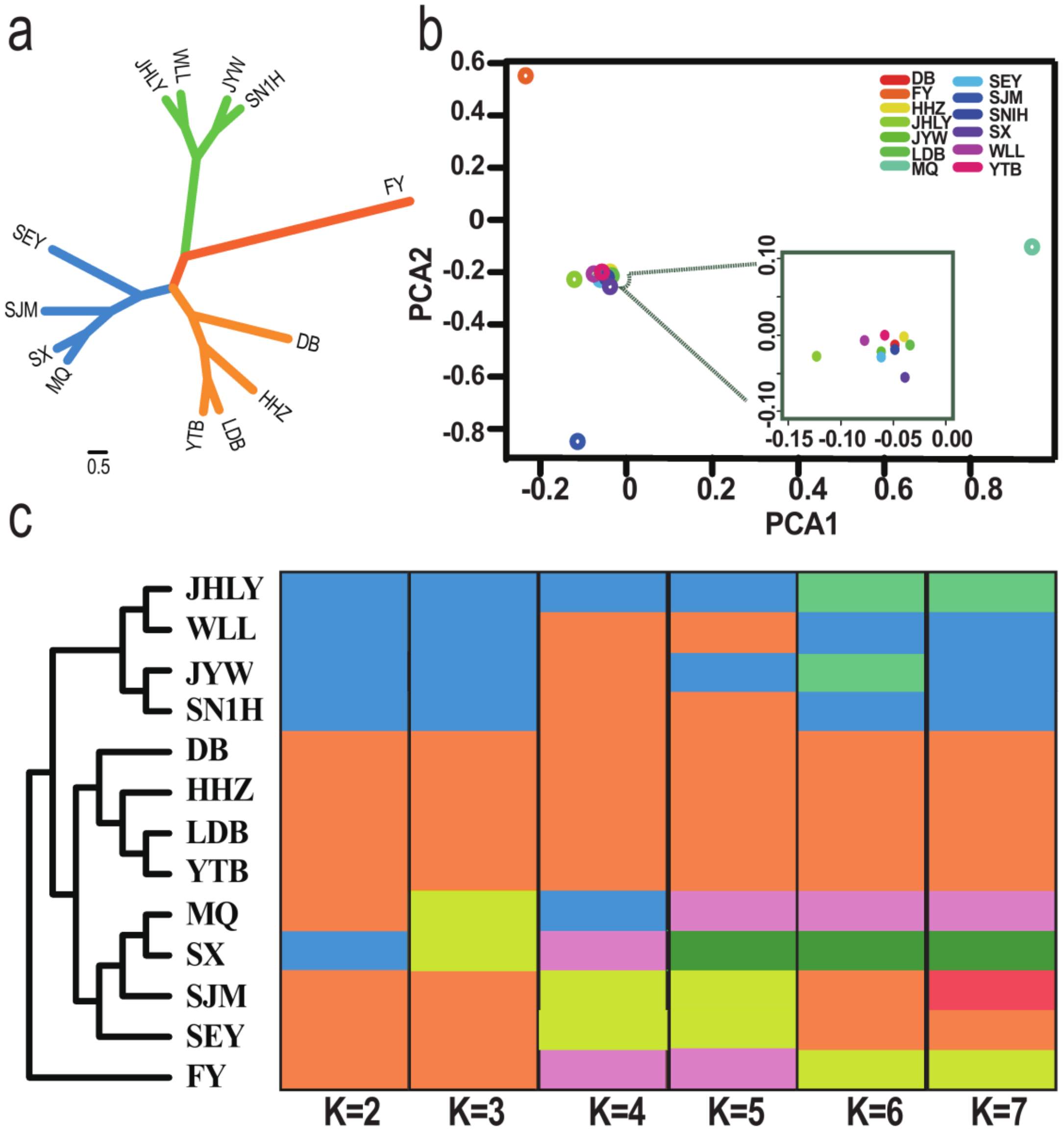

Figure 3 Simplified diagram of polyphenols biosynthetic pathway. (**a**) Simplified diagram of polyphenols biosynthetic pathway. Numbers in brackets represent genes' copy number. (**b**) PCA scatter plot of 9 samples using genes related to polyphenols biosynthetic pathway. (**c**) Neighbor- joining tree of the F3'H, ANR, and LAR from longan, peach, orange, Arabidopsis and grape. (**d**) Cluster analysis of expression profiles of *F3'H*, *ANR*, and *LAR*. The bar represents the scale of relative expression levels of genes, and colors indicate relative signal intensities of genes. Each column represents a sample, and each row represents a single gene.

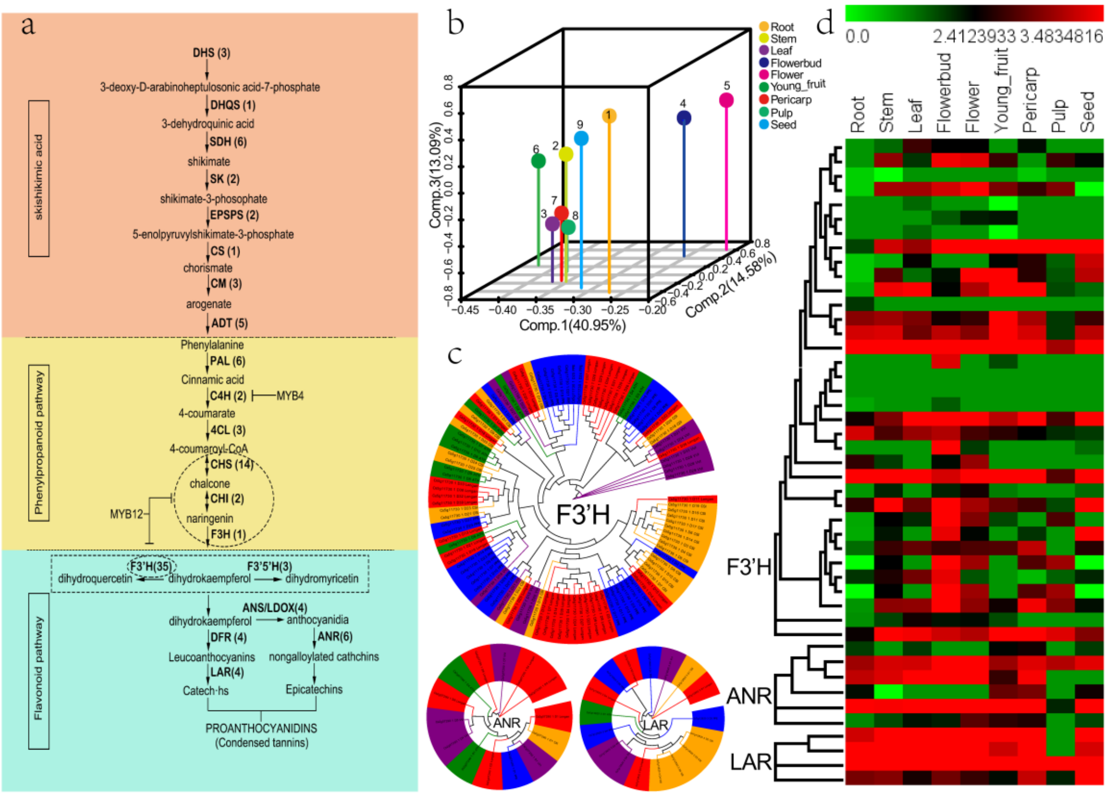

Click here to download Figure Figure 4 The MYB transcription factor in longan genome.doc

Figure 4 The MYB transcription factor in longan genome. (**a**) Numbers of the members in the three different MYB classes in Arabidopsis, orange, longan, peach, and grape. (**b**) Neighbor- joining tree of the *MYB* gene family. (**c**) PCA scatter plot of 9 samples using 94 R2R3-MYB genes. (**d**) Cluster analysis of expression profiles of MYB transcription factor. The bar represents the scale of relative expression levels of genes, and colors indicate relative signal intensities of genes. Each column represents a sample, and each row represents a single gene.

a

Type	*A. thaliana*	*C. sinensis*	*D. longan*	*P. persica*	*V. vinifera*
R2R3-MYB	141	71	98	88	116
3R-MYB	5	0	0	0	1
4R-MYB	1	1	0	0	1

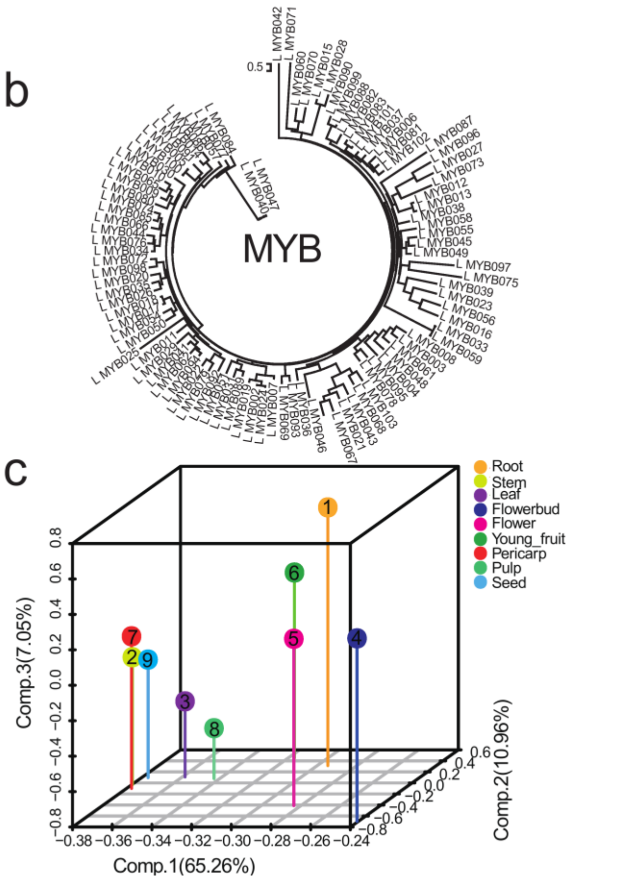

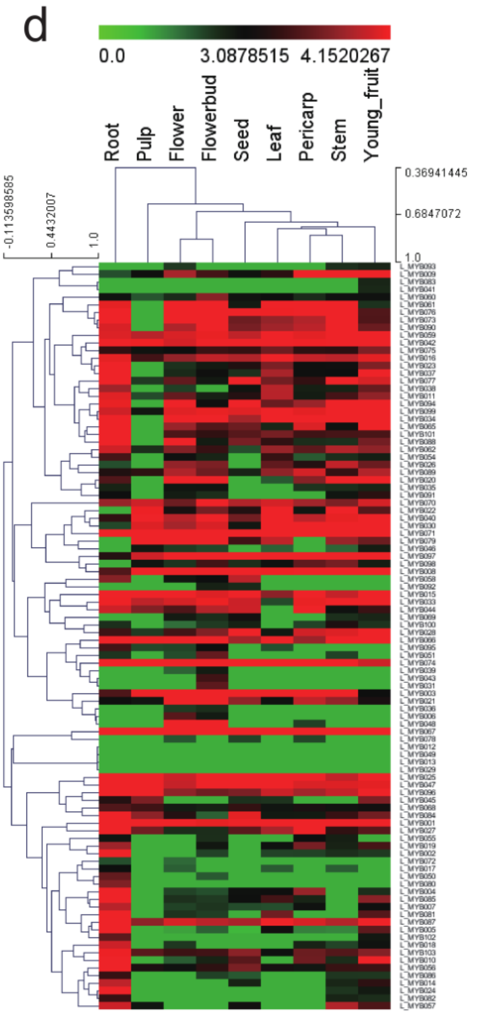

Supplemental Tables

Click here to access/download
Supplementary Material
Supplemental Tables.doc

Click here to access/download
Supplementary Material
Supplemental Figures.doc

Click here to access/download
Supplementary Material
Supplementary EXCEL file 1-D.longan tanscription
factor identification. sta.xlsx

Click here to access/download
Supplementary Material
Supplementary EXCEL file 2 D. longan genefamily.decrease.ipr.xls

Click here to access/download

Supplementary Material

Supplementary EXCEL file 3 D. longan genefamily-expansion.xls

Click here to access/download
Supplementary Material
Supplementary EXCEL file 4 FY, SN1H, MQ, LDB, and JYW SNP analysis-r1.filter.anno.xls

Click here to access/download
Supplementary Material
Supplementary EXCEL file 5 SX, and YTB SNP analysi
-r2.filter.anno.xls

Click here to access/download

Supplementary Material

Supplementary EXCEL file 6 Statistics of genes copy numbers of biosynthesis of polyphenols in different plants.xls

Click here to access/download

Supplementary Material

Supplementary EXCEL file 7 Genes expression levels of biosynthesis of polyphenols in longan.xls

Click here to access/download
Supplementary Material
Supplementary EXCEL file 8 longan MYB genes expressed in 9 different samples.xls
