# Supplementary material for: Genome-wide sequencing of longan (Dimocarpus longan Lour.) provides insights into molecular basis of its polyphenol-rich characteristics
Source: Gigascience. 2017 Mar 28;6(5):1–14. doi: 10.1093/gigascience/gix023 (PMC5467034; doi:10.1093/gigascience/gix023)
Supplement: GIGA-D-16-00042_Revision_3.pdf [file gix023_GIGA-D-16-00042_Revision_3.pdf]

Manuscript Click here to download Manuscript Giga_2017-2-5(submission).doc

Genome-wide sequencing of longan (*Dimocarpus longan* Lour.) provides insights into molecular basis of its polyphenol-rich characteristics

YuLing Lin[1*], JiuMeng Min[2*], RuiLian Lai[1], ZhangYan Wu[2], YuKun Chen[1], LiLi Yu[2], ChunZhen Cheng[1], YuanChun Jin[2], QiLin Tian[1], QingFeng Liu[2], WeiHua Liu[1], ChengGuang Zhang[2], LiXia Lin[1], YanHu[2], DongMin Zhang[1], MinKyaw Thu[1], ZiHao Zhang[1], ShengCai Liu[1], ChunShui Zhong[1], XiaoDong Fang[2], Jian Wang[2,3], Huanming Yang[2, 3], Rajeev K Varshney[4,5&], YeYin[2&], ZhongXiong Lai[1&]

[1]Institute of Horticultural Biotechnology, Fujian Agriculture and Forestry University, Fuzhou, Fujian 350002, China

[2]BGI-Shenzhen, Shenzhen 518083, China

[3]James D. Watson Institute of Genome Sciences, Hangzhou 310058, China

[4]International Crops Research Institute for the Semi-Arid Tropics (ICRISAT), Hyderabad, India

[5]School of Plant Biology, The University of Western Australia, Crawley, Perth, Australia

*Equal contributor

[&] Corresponding authors

Email addresses:

YLL: buliang84@163.com

JMM: minjm@genomics.cn

RLL: 1044612364@qq.com

ZYW: Joanna.wu@genomics.cn

YKC: cyk68@163.com

LLY: yulili@bgitechsolutions.com

CZC: 405553272@qq.com

YCJ: jinyuanchun@genomics.cn

QLT: 563430138@qq.com

QFL: liuqingfeng@bgitechsolutions

WHL: 695471647@qq.com,

CGZ: zhangchengguang@genomics.cn

LXL: 907466498@qq.com

YH: ewa.hu@bgitechsolutions.com

DMZ: 419418882@qq.com

MKT: 1175025328@qq.com

ZHZ: zhangzihao863@126.com

SCL: 1215698900@qq.com

CSZ: 291768260@qq.com

XDF: fangxd@genomics.cn

JW: wangjian@genomics.org.cn

HMY: hmyang@genetics.ac.cn

RKV: R.K.Varshney@CGIAR.ORG

YY: yinye@genomics.cn

ZXL: laizx01@163.com

Abstract

Background: Longan (*Dimocarpus longan* Lour.), an important subtropical fruit in the family *Sapindaceae*, is grown in more than ten countries. Longan is an edible drupe fruit and a source of traditional medicine with polyphenol-rich traits. Tree size, alternate bearing, and witches' broom disease still pose serious problems. To gain insights into the genomic basis of longan traits, a draft genome sequence was assembled.

Results: The draft genome (about 471.88 Mb) of a China longan cultivar, 'Honghezi', was estimated to contain 31,007 genes and 261.88 Mb of repetitive sequences. No recent whole-genome wide duplication event was detected in the genome. Whole-genome resequencing and analysis of 13 cultivated *D. longan* accessions revealed the extent of genetic diversity. Comparative transcriptome studies combined with genome-wide analysis revealed polyphenol-rich and pathogen-resistance characteristics. Genes involved in secondary metabolism, especially those from significantly expanded (*DHS*, *SDH*, *F3'H*, ANR, and *UFGT)* and contracted (*PAL*, *CHS*, and *F3'5'H*) gene families with tissue-specific expression, may be important contributors to the high accumulation levels of polyphenolic compounds observed in longan fruit. The high number of genes encoding nucleotide-binding site leucine-rich repeat (NBS-LRR) and leucine-rich repeat receptor-like kinase proteins, and the recent expansion and contraction of the NBS-LRR family suggested a genomic basis for resistance to insects, fungus, and bacteria in this fruit tree.

Conclusions: These data provide insights into the evolution and diversity of the

longan genome. The comparative genomic and transcriptome analyses provided information about longan-specific traits, particularly genes involved in its polyphenol-rich and pathogen- resistance characteristics.

Keywords: longan genome; genetic diversity; polyphenols biosynthesis; pathogen resistance

Background

Dimocarpus longan Lour. (*D. longan*) originated from South China or Southeast Asia and is commonly called longan or 'dragon eye' in Asia. It is an important tropical/subtropical evergreen fruit tree that has a diploid genome ($2n=2x=30$) and belongs to the family Sapindaceae. Longan is widely cultivated in Southeast Asia, South Asia, Australia, and Hawaii [1]. China's longan acreage and production rank first, accounting for 70% and more than 50% of the world's acreage and production, respectively [2]. As an edible drupe fruit and source of traditional medicine, longan is grown in most areas of Southern China, including Guangdong, Guangxi, Fujian, Sichuan, Yunnan, and Hainan [3]. Traditionally, longan leaves, flowers, fruit, and seeds all have been widely used as traditional Chinese medicines for several diseases, including leucorrhea, kidney disorders, allergies, cancer, diabetes, and cardiovascular disease, because they contain bioactive compounds such as phenolic acids, flavonoids, and polysaccharides [4-6]. However, tree size, alternate bearing, and witches' broom disease still pose serious problems in longan production [1]. Cultivar identification and characterization are the first steps for fruit introduction and breeding improvement [7]. In China, there are more than 300 longan varieties; most are

landraces and farm varieties, although a few wild populations exist in Hainan, Guangdong, Guangxi, and Yunnan provinces [7, 8]. However, only 30–40 varieties are grown commercially worldwide. Longan breeding improvement via conventional breeding strategies has been hindered by its long juvenility, genetic heterozygosity, and plant size [1]. To identify cultivars and improve longan breeding, knowledge of the longan genetic background is required.

Recently, many draft genome sequences for fruit trees have become available, including papaya (*Carica papaya*) [9], grape (*Vitis vinifera*) [10], apple (*Malus domestica*) [11], plum (*Prunus mume*) [12], orange (*Citrus sinensis*) [13], peach (*Prunus persica*) [14], pear (*Pyrus bretschneideri*) [15], kiwifruit (*Actinidia chinensis*) [16], pineapple (*Ananas comosus*) [17], banana (*Musa acuminata*) [18], jujube (*Ziziphus jujuba*) [19], and strawberry (*Fragaria vesca*) [20]. However, draft genome sequences are still lacking for the subtropical and tropical fruits of the Sapindaceae family. The Sapindaceae family, known as the Soapberry family, is part of the dominant plants in the tree layer of the tropical rain forests; it includes the subtropical and tropical fruits (longan, *Litchi chinensis*, and *Nephelium lappaceum*), the important bioenergy plant soapberry (*Sapindus mukorossi*), and the woody oil plant brook feather (*Xanthoceras sorbifolia*). To accelerate improved breeding and utilization of the Sapindaceae family, a fundamental understanding of its complete genome sequence is crucial. Longan, as one of famous fruit trees in Sapindaceae family, was selected for genome sequencing in this study. Here, we report the draft genome sequence of the longan cultivar 'Honghezi' (HHZ) ($2n=2x=30$) and the extent of

genetic diversity in this species based on whole genome re-sequencing of 13 cultivated *D. longan* accessions. Comparative transcriptome studies combined with genome-wide analysis provided insights into the structure and evolution of the longan genome, the molecular mechanisms of the biosynthesis of polyphenol, and the pathogen resistance of longan. Together, these results provided insights into the evolution and diversity of the longan genome, and will help to improve the efficiency of longan conventional breeding by integrating biotechnological tools.

Results

Genome sequencing and assembly

We selected the *D. longan* 'HHZ' cultivar for genome sequencing. In brief, a total of 316.84 Gb of raw data was generated by Illumina sequencing of 12 genome shotgun libraries with different fragment lengths ranging from 170 bp to 40 kb (Additional file 1: Table S1). After stringent filtering and correction steps, a total of 121.68 Gb of high-quality sequence data, representing 273.44-fold coverage of the entire genome, were obtained (Additional file 1: Table S2). Based on K-mer frequency methods [21], the *D. longan* genome was estimated to be 445 Mb with a 0.88% heterozygosity rate (Additional file 2: Fig. S1, Additional file 1: Table S3). Compared with other sequenced fruit trees genomes, the *D. longan* genome was bigger than papaya [9], orange [13], peach [14], and plum [12], and smaller than grape [10], apple [11], pear [15], pineapple [17], and kiwifruit [16]. Longan trees are generally thought to have highly heterozygous traits. The estimated 0.88% heterozygosity rate in the whole genome of the longan 'HHZ' cultivar is reported here for the first time. This

heterozygosity rate is higher than the rates reported for kiwifruit (0.536%) [16], plum (0.03%) [12, 22], and poplar (about 0.5%) [23], and lower than the rates for pear (1–2% sequence divergence) [15] and pineapple (1.89% in F153, 1.98% in MD2, 2.93% in CB5) [17]. These results imply that the idea that fruit trees always have high heterozygosity may be due to artificial grafting and/or asexual reproduction.

Using the SOAPdenovo program [24], all the high-quality reads were assembled into 51,392 contigs and 17,367 scaffolds (≥200 bp) totaling 471.88 Mb excluding gaps (Table 1). These assembled sequences accounted for approximately 106.04% of the estimated longan genome, which conflicts with previously reported genome assemblies where the sequences accounted for less than 100% of the estimated genome [13-15]. The higher percentage might be due to the high heterozygosity of the longan genome, suggesting that, in the future, a single- molecule sequencing technology should be used to correct the longan genome assembly. Here, the N50s of contigs and scaffolds were 26.04 kb (longest, 173.29 kb) and 566.63 kb (longest, 6942.32 kb), respectively (Table 1), suggesting the high quality of the assembly. The GC content of the *D. longan* genome was 33.7%, which is comparable with the GC content of the genomes of pineapple (33%) [17], jujube (33.41%) [19], and orange (34.06%) [13], but lower than the GC content of the genomes of kiwifruit (35.2%) [16], papaya (35.3%) [9], and grape (36.2%) [10] (Table 2, Additional file 2: Fig. S2). Analysis of the percent GC content among different fruit trees can provide important clues about gene density, gene expression, replication timing, recombination, and evolutionary relationships [25]. The GC-depth graph and distribution indicated no

contamination of any bacterial sequence in the longan genome assembly, and 99.2% of the assembly was sequenced with more than 20× coverage (Additional file 2: Fig. S3). The statistics and comparison of the *D. longan* assembly with 12 other twelve fruit tree genomes are shown in detail in Table 2. The quality of the assembly was assessed by aligning the scaffolds to a longan transcriptome assembly from the NCBI Sequence Read Archive (SRA) [SRA050205]. Of the 96,251 longan transcriptome sequences (≥100) reported previously [26], 97.55% were identified in the genome assembly (Additional file 1: Table S4), confirming the high quality of the assembly.

BUSCO analysis

We further evaluated the quality and completeness of the draft longan genome assembly using the BUSCO (Benchmarking Universal Single-Copy Orthologs) datasets [27]. Of the total of 956 BUSCO ortholog groups searched in the longan assembly, 900 (94%) BUSCO genes were “complete single-copy”, 288 (30%) were “complete duplicated”, 16 (1.6%) were “fragmented”, and 40 (4.1%) were “missing” (Additional file 1: Tables S5). The percentage of missing BUSCO genes was comparable to the percentages missing in the assemblies of banana (3%), *Brassica napus* (3%), and Arabidopsis (2%), which have served as well-assembled standards at the chromosomal level [28], further suggesting the high quality of our assembly.

Repetitive elements and gene annotation

Repetitive elements are major components of eukaryotic genomes, and they have been used extensively to analyze genome structure, karyotype, ploidy, and evolution. In the longan assembly, we found a total of 261.88 Mb (52.87%, 445 Mb) was repetitive

sequences (Additional file 1: Table S6), which is higher than the amount observed in orange (20%, 367 Mb) [13], peach (29.6%, 265 Mb) [14], kiwifruit (36%, 758 Mb) [16], pineapple (38.3%, 526 Mb) [17], grape (41.4%, 475 Mb) [10], jujuba (49.49%, 444 Mb) [10], and papaya (51.9%, 372 Mb) [9], and lower than the amount reported in pear (53.1%, 527 Mb) [15] and apple (67.4%, 742.3 Mb) [11] (Table 2), indicating that the size of fruit tree genomes differed as a result of the variable amounts of repetitive elements that they contained. Accordingly, the bigger plant genomes often possessed higher percentages of repetitive elements than the smaller plant genomes. Most plant genomes appear to contain abundant long-terminal repeat (LTR) retrotransposons and a small number of short interspersed elements (SINEs) and long interspersed elements (LINEs) [29]. We found that the repetitive fraction of the longan genome comprised LTR retrotransposons, which were the most abundant (36.54%), and SINEs (2.43%) and LINEs (0.04%), which were the least abundant; other repeats, including tandem repeats and unknown repeats, made up 7.59% and 7.71% of the repetitive fraction, respectively (Additional file 1: Table S7). A large number of the unknown repetitive sequences may be longan-specific. The characterization of repetitive sequences is of primary importance for understanding the structure and evolution of the longan genome.

Using a combination of *de novo* prediction, homology-based searches, and a transcriptome assembly, we predicted a total of 39,282 genes yielding a set of 31,007 high-quality proteins in the longan genome. The average gene size was 3,266.02 bp, the average length of the coding sequence was 1,232.18 bp, and the average number

of exons per gene was 4.68 (Additional file 1: Table S8). The number of genes predicted in the longan genome was close to the number of genes predicted in jujube (32,808) [10], higher than in papaya (24,746) [9], pineapple (27,024) [17], peach (27,852) [14], orange (29,445) [13], and grape (30,434) [10], and lower than in kiwifruit (39,040) [16], pear (42,812) [15], and apple (57,386) [11]. This analysis showed that the number of genes in the longan genome was similar to the numbers found in other sequenced fruit tree genomes of equivalent size, and also indicated that the bigger plant genomes usually contained higher numbers of genes. Of 31,007 protein-coding genes, 27,862 (89.86%) had TrEMBL homologs, 22,986 (74.13 %) had SwissProt homologs, and 23,398 (75.46%) had InterPro homologs (Additional file 1: Table S9). A total of 1,611 putative transcription factors (TFs) distributed in 64 families were identified, which represented 4.1% of the genes in the longan genome (39,282). The percentage of TFs in longan genome was close to the percentages reported in strawberry (4.6%) [20], and rice (4.8 %), but lower than the percentages in Arabidopsis (6%), kiwifruit (6.2%) [16], grape (6.7%) [30], poplar (6.7%), and banana (11.75%) [18]. In the longan genome, the largest numbers of genes encoded TFs in the following TF families: MYB (186 genes), ERF (115), MADS (109), NAC (107), bHLH (107), C2H2 (98), B3 superfamily (86), HB (71), WRKY(58), bZIP (55), GRAS (52), and C3H (49) (Supplemental EXCEL File 1). The identification of these TFs will help to lay a solid foundation for functional verification of longan traits in the future. Among the non-coding genes detected in the longan genome assembly, we identified 359 microRNAs, 212 rRNA, 506 tRNAs, and 399 small nuclear RNAs

(Additional file 1: Table S10).

Gene family evolution and comparison

Orthologous clustering analysis was conducted with the longan genome and eight other selected plant genomes, Arabidopsis, orange, papaya, grapevine, banana, peach, kiwifruit, and apple. Of the 31,007 protein-coding genes in the genome, 26,261 were grouped into 14,961 gene families (763 of which were longan-unique families) giving an average of 1.76 genes per family (Additional file 1: Table S11). The remaining 5,834 genes were classed as un-clustered genes. Among the 31,007 genes, 4,653 were longan-unique paralogs, 5,184 were multiple-copy orthologs, 3,606 were single-copy orthologs, and 12,818 were other orthologs (Fig. 1b). Comparative analysis of the longan genome with eight other selected plant genomes indicated that the number of gene families in the longan genome was similar to the numbers in the genomes of orange (15,000) [13] and peach (15,326) [14], higher than in banana (12,519) [18], Arabidopsis (13,406), grape (13,570) [10], kiwifruit (13,702) [16], and papaya (13,763) [9], and lower than in apple (17,740) [11] (Fig. 1b, Additional file 1: Table S11). These comparisons indicated that differences in gene families in plant genomes may be important sources of genetic traits and adaptation in different species. Comparative analysis of the longan genome with the genomes of citrus, banana, peach, and Arabidopsis showed that these five species contained a core set of 9,215 genes in common, whereas 1,207 genes were specific to longan, which is more than the numbers of genes specific to citrus and Arabidopsis, and lower than the numbers specific to *M. acuminate* and peach (Fig. 1d).

Expansion or contraction of gene families may provide clues to the evolutionary forces that have shaped plant genomes and have an important role in the diversification of plants. In this study, we used CAFÉ [31] to identify gene families that had potentially undergone expansion or contraction in the longan genome. We found a total of 2,849 expanded gene families and 2,842 contracted families; however, only 386 expanded families (7,839 genes) and 12 contracted families (53 genes), accounting for 19.96% and 0.13% of the total coding genes (39,282), respectively, were found to be statistical significant at $P < 0.05$ (Supplemental EXCEL Files 2 and 3). The genes in the significantly expanded and contracted families ($P < 0.05$) were annotated with gene ontology (GO) terms. Genes in a total of 32 (expanded) and 11 (contracted) families were assigned GO terms under the three GO categories, biological process, cellular component, and molecular function. Almost all the expanded or contracted families contained genes that were assigned terms under biological process, and a few genes in the contracted families were assigned terms under the cellular component and molecular function categories (Additional file 2: Fig. S4a, b). The dominant terms in the expanded or contracted gene families were 'cellular component organization', 'locomotion', 'auxiliary transport protein', and 'binding', revealing important clues to the evolutionary forces that may have shaped the longan genomes.

Genome evolution

Whole-genome duplication is common in most plant species and it represents an important molecular mechanism that has shaped modern plant karyotypes [32].

Characterization and annotation of the longan genome provided comprehensive information for us to further investigate the evolutionary history of longan. Single-copy nuclear genes from orange, Arabidopsis, cacao (*Theobroma cacao*), poplar (*Populus trichocarpa*), grape, apple, papaya, soybean, peach, kiwifruit, and banana [18] were used in a genome-scale phylogenic analysis using the maximum likelihood method. The phylogenetic analysis showed that longan was phylogenetically closest to orange, close to papaya, Arabidopsis, and cacao, and most distant from monocotyledon fruits (banana). From the phylogenetic tree, we estimated that longan diverged about 69.3 million years ago (Fig. 1a). To determine the nature of the evolutionary events that led to the modern longan genome structure, we analyzed the syntenic relationships between longan and poplar. We detected a total of 2,106 and 883 syntenic blocks containing 17,901 and 17,447 colinear genes for longan and poplar, respectively (Additional file 1: Table S12), which supported the reported conserved colinearity and close evolutionary relationship in these two plant species. To further analyze the evolutionary divergence and the relative age of duplication events in longan and other related species, we calculated the distance–transversion rates at fourfold degenerate sites (4DTv) (Fig. 1c). The 4DTv value peaked at 0.5 for paralog pairs in grape, highlighting the recent whole-genome duplication in this species. Two 4DTv values that peaked at 0.72 and 0.6 for orthologs between longan and banana, and between longan and Arabidopsis, respectively, supported species divergence. These results are consistent with the more ancient divergence between monocotyledons and dicotyledons. The orthologs between longan and grape, longan

and peach, and longan and orange showed 4DTv distances peaks at 0.36, 0.36, and 0.26, respectively, which is consistent with the 4DTv peaks reported previously for Vitaceae and Rosaceae species, and more ancient than the 4DTv values for Rutaceae or Sapindaceae. In longan, the analysis showed ancient duplication events (the 4DTv peak at about 0.55) but did not reveal a recent whole-genome duplication. These results complement the results for the longan genome and will contribute to studies into ancestral forms and arrangements of plant genes [33].

Assessment of genetic diversity in longan germplasm

A representative characteristic of longan cultivars is their high heterozygosity, which has resulted in the low efficiency of longan germplasm management and utilization. Traditionally, molecular markers (RAPD, AFLP, SCAR, SCTP, and SRAP) and single nucleotide polymorphisms (SNPs) based on transcriptome data [34] have been used for accurate identification of longan varieties. However, the extent of heterozygosity in the whole genome is not well understood [7]. The availability of the longan draft genome provided the foundation for a comprehensive assessment of heterozygosity in the longan genome.

We selected 13 representative commercially cultivated accessions with early-maturing, middle-maturing, late-maturing, multiple-flowering, aborted-seeded, and disease- resistant characteristics for whole-genome resequencing (Additional file 1: Table S13). A total of 45.77 Gb of raw data were generated by Illumina sequencing. After alignment of the clean reads corresponding to 5.02- to 7.31-fold depths and >78% coverage to the reference genome (Additional file 1: Table S14), we identified

357,737 SNPs (Additional file 1: Table S15), and 23,225 small insertions/deletions (indels) (Additional file 1: Table S16). The overall polymorphism density was 0.05–0.12 SNPs and 0.004–0.007 indels per 10 kb of the genome sequence, which is much lower than the diversity reported in orange [13]. Notably, the major variations existed among the ‘FY’, ‘MQ’, and ‘SJM’ accessions, whereas variations within the cultivated longan accessions, particularly the ‘LDB’ accessions, were relatively low (Additional file 1: Tables S15 and S16).

To further investigate the population structure and relationships among the longan accessions, we constructed a neighbor-joining tree (Fig. 2a) and carried out a principal component analysis (PCA) (Fig. 2b). The neighbor-joining tree, constructed based on all the identified SNPs, indicated that the 13 longan accessions clustered into two subfamilies. The first subfamily consisted only of ‘FY’, which showed the highest variations and clear separation from other cultivars. This result is quite different from results reported previously [35, 36]. In previous studies using molecular markers, ‘FY’, which originated from Quanzhou, China, was found to cluster together with other Chinese longan accessions. In our study, which was conducted at an overall genomic level, ‘FY’ was found to possess more genetic differences compared with the other longan accessions tested. This result might be due to the special traits of ‘FY’, such as witches' broom disease-resistant, middle-maturity, and canned processing products. This result also supports the observed diversity of ‘FY’ at the overall genomic level. The second subfamily neighbor-joining tree consisted of three clades (Fig. 2a). The first clade included ‘JHLY’, ‘WLL’, ‘JYW’, and ‘SN1H’; the second

contained ‘MQ’, ‘SX’, ‘SJM’, and ‘SEY’; and the third consisted of ‘DB’, ‘HHZ’, ‘LDB’ and ‘YTB’. Moreover, the PCA showed that the samples that originated from China tended to cluster together (‘HHZ’, ‘DB’, ‘JYW’, ‘LDB’, ‘WLL’, ‘SN1H’, ‘YTB’, ‘SEY’, ‘JHLY’, and ‘SX’). The PCA also showed the clear separation of ‘FY’, ‘SJM’, and ‘MQ’. The ‘SJM’ and ‘MQ’ accessions, which originated from Southeast Asia and Thailand, respectively, possessed apparent differences compared with the Chinese longan accessions tested in this study. Together these results indicated geographic patterns of genetic differentiation, which agree with findings reported previously [34]. The relatively low levels of genetic variation among the Chinese cultivars also suggested that they might have suffered a bottleneck during domestication [7, 34]. These results suggested the relationship among the 13 selected longan accessions was, at least partly, determined by their geographical distributions.

An additional analysis of the population structure was conducted using the FRAPPE program [37] with K (the number of populations) set from 2 to 7 (Fig. 2c). For K=7, a new subgroup was detected among the 13 longan accessions. This subgroup had characteristics, such as various maturity levels, high yielding, aborted-seeding, disease-resistant, and multiple flowering. The cultivars ‘SX’ and ‘YTB’, which are susceptible to disease, contained more variations in resistance genes, such as NBS-LRR and LRR-RLK, than the disease resistant cultivars (‘FY, ‘SN1H, ‘MQ’, ‘LDB’, and ‘JYW’) (Supplemental EXCEL Files 4 and 5). These results provided a measure of the changes in genetic diversity and a theoretical estimate of the genetic relationships among the selected longan cultivars.

RNA sequencing revealed SNPs, indels, differentially expressed genes, and alternative-splicing events in different tissues of 'SJM' longan

To improve the gene annotation of the longan genome sequence and get more information about longan traits, we constructed nine cDNA libraries corresponding to nine different organs (root, stem, mature leaf, flower bud, flower, young fruit, pericarp, pulp, and seed) from a representative 'SJM' cultivar. 'SJM', which originated in Southeast Asia, blossoms and bears fruit throughout the year, with no requirement of environmental control [38]. Here, a total of 490,502,822 clean reads from nine RNA sequencing (RNA-seq) data sets were obtained after removing low-quality reads and adaptor sequences, and about 53.55–79.40% of the clean reads mapped to the longan draft genome (Additional file 1: Table S17). This percentage of mapped reads is lower than the 90% previously reported in peach [39], suggesting that the 'SJM' cultivar contained high variations compared with the sequenced 'HHZ' genome, probably because of their different origins. Moreover, the BUSCO analysis [27] showed that 483 (87%) of BUSCO genes were "complete single-copy", 352 (36%) were "complete duplicated", 53 (5.5%) were "fragmented", and 68 (7.1%) were "missing" (Additional file 1: Table S18), indicating the high quality of our assembled transcriptome.

The transcribed regions/units were constructed independently for individual tissues. We found that transcripts/genes ranged from 19,322 (pulp) to 23,118 (flower bud), completely or partially (49.18–58.85%) overlapped with 39,282 annotated genes in the longan genome. The numbers of expressed transcripts in each longan tissue were

much lower than the numbers previously reported in *Brassica rapa* (32,335 genes expressed in at least one tissue, equivalent to 78.8% of the 41,020 annotated genes) [40]. The lower numbers of transcripts detected in each tissue, may be due to the high variations and genetic heterozygosity in the 'SJM' cultivar. The coverage of the longan gene set by our transcripts indicated the broad representation of our unigenes, and provided the opportunity to identify alternative splicing (AS) events. In addition to the predicted genes, novel transcripts, ranged from 1,621 (stem) to 1,999 (young fruit), were detected across all nine samples. Among the novel transcripts, 798 (flower) – 988 (young fruit) contained open reading frames, while 820 (stem) – 1,011 (young fruit) were identified as non-coding RNAs in the longan genome (Additional file 1: Table S17). Most of these non-coding RNAs were longer than 200 nt and had no ORFs encoding sequences longer than 300 amino acids, suggesting they may be long intergenic non-coding RNAs [41] or *cis*-natural antisense transcripts [42], which will need further analysis. The numbers of novel encoding and non-coding transcripts in young fruit were the highest among the nine samples, suggesting the development of young fruit required more complicate gene regulatory networks than the other stages. To further optimize of the structure of the transcripts, we compared the assembled transcripts and annotated genes from the reference longan genome and extended the 5′ or 3′ ends of the transcripts according to the annotated gene information. In total, the extending 5′ or 3′ end of annotated genes ranged from 8,126 (pulp) to 9,995 (flower bud) across nine tissues, and about almost half the number of total genes extended by 5′ end in each sample. We identified a total of 1,255,816

SNPs and 34,390 indels across the nine longan tissues, and found that the highest number of SNPs and indels were detected in young fruit (161,897) and leaf (4,673), respectively, suggesting the expressed transcripts may be more diverse in these two tissues. Notably, the lowest frequencies of SNPs and indels were detected in pulp (105,007 and 2,587 respectively). The SNPs and indels detected in the transcript sequences will be a valuable resource from which to identify candidate genes, analyze population structures and evolution, and accelerate plant breeding [39]. The identification of novel genes extended annotated genes, SNPs, and indels from different developmental stages, imply our gene set can serve as a valuable complementary resource for longan genomics.

To identify significantly differentially expressed genes (DEGs), we used 12 pair-wise comparisons among the nine samples as follows: root *VS* stem, root *VS* leaf, leaf *VS* stem, flower bud *VS* flower, flower bud *VS* young fruit, flower *VS* young fruit, young fruit *VS* pulp, young fruit *VS* seed, pericarp *VS* pulp, pericarp *VS* seed, and pulp *VS* seed. Among the detected DEGs (Additional file 2: Fig. S5), an average of 3,922±2,391 were up-regulated and an average of 4,859±2,666 were down-regulated in the 12 comparisons. The highest number of DEGs was detected in young fruit *VS* seed (9,737), followed by root *VS* leaf (9,702) and flower *VS* young fruit (9,101), and the lowest number of DEGs was detected in flower bud *VS* flower (3,722). The numbers of organ-specific genes ranged from 87 in young fruit to 530 in root, and the significantly differentially expressed transcription factors in each comparison ranged from 272 (flower bud *VS* flower) to 732 (young fruit *VS* pulp). To evaluate the

potential functions of the DEGs, we annotated them by assigning GO terms under the three main categories, biological process, cellular component, and molecular function. DEGs in each pair were categorized into 43 (flower bud *VS* flower) - 47 (young fruit *VS* pulp). Details of the GO annotations are provided in Additional file 2: Fig. S6. The dominant terms in all 12 comparisons were ‘Metabolic process’, ‘Cellular process’, ‘Cell’, ‘Cell part’, ‘Catalytic activity’, and ‘Binding’, which is similar to results previously reported in the ‘SJM’ and ‘LDB’ cultivars [43]. To further understand the biological functions of the DEGs, we carried out a KEGG (Kyoto Encyclopedia of Genes and Genomes) pathway-based analysis. In nine of the 12 comparisons, the highest numbers of DEGs were involved in ‘metabolic pathway’, followed by the ‘biosynthesis of secondary metabolites’ and ‘plant–pathogen interaction’ pathways. In pericarp *VS* seed, root *VS* leaf, and pericarp *VS* pulp, ‘biosynthesis of secondary metabolites’, ‘pyrimidine metabolism’, and ‘stilbenoid, diarylheptanoid and gingerol biosynthesis’ were the most represented pathways, respectively (Additional file 2: Fig. S7). These results are fully consistent with the view that *D. longan* contains high levels of polyphenolic compounds, and a large number of pathogen resistance genes [44, 45].

To determine the types of AS events represented in our assembled transcripts data set, we used the TopHat software [46]. First, the nine longan tissues were analyzed at the exon level, which can provide important information about the types of gene isoforms that are expressed and variable [47]. Expressed exons were detected in the range of 96,105 (pulp) to 111,476 (flower bud) across the nine tissues (Additional file 1: Table

S17). A total of 298,914 AS events were detected across all the tissues, representing the four known types of AS, namely intron retention, exon skipping, alternative 5′ splice site donor, and alternative 3′ splice site acceptor. Alternative transcripts have been shown to be tissue- or condition-specific [47, 48]. We also found that the largest numbers of AS events were detected in leaf (37,216), followed by young fruit (35,998), and pericarp (35,384), and the smallest numbers were found in pulp (28,058), corresponding to the least expressed exons. The predominant and rare types of AS events in all nine tissues were intron retention and exon skipping, respectively. This result is consistent with prior findings in rice [49], Arabidopsis [50], grape [48, 51], and *B. rapa* [40], but contradicts a previous finding that exon-skipping was predominant in peach [39] and metazoans [52], indicating the complexity of the AS landscape in plants and the important consequences this may have on plant/crop phenotypes.

Biosynthesis of polyphenols and MYB transcription factors in longan

Polyphenols, potential antioxidative compounds, are the major category of secondary metabolites in longan leaf, flower, fruit, and seed [4]. Phenolic compounds are derived primarily through the shikimic acid, phenylpropanoid, and flavonoid pathways. Our transcriptome data showed that the significant DEGs in the nine longan tissues were involved mainly in ‘biosynthesis of secondary metabolites’. To further assess changes between the primary and secondary metabolism of polyphenols during the longan vegetative and reproductive growth stages, the copy numbers of 26 selected structural genes within the shikimate acid, phenylpropanoid, and flavonoid biosynthesis

pathways were compared with those in corresponding pathways of Arabidopsis, orange, peach, grape, poplar, and eucalyptus (Fig. 3a, Supplemental EXCEL File 6). Comparison analysis showed that the 26 structural genes showed up and down variations in copy numbers among the seven plants tested (Supplemental EXCEL File 6). The significant expanded gene families in longan, orange, peach, poplar, and eucalyptus were *DHS*, *SDH*, *F3′H*, *ANR*, and *UFGT*, when compared with the corresponding families in grape, which is considered to be the oldest among the seven selected plants in evolutionary history [53]. SDH, catalyzes the NADPH-dependent reduction of 3-dehydroshikimate to shikimate in the fourth step of the shikimate pathway, which is the metabolic route required for the biosynthesis of the aromatic amino acids. *SDH* had six copy numbers in longan, which is the same as in Populus, but much higher than in Arabidopsis (1 copy), peach and grape (2 copies each), and orange and eucalyptus (3 copies each). F3′H is involved in flavonoid biosynthesis and is important for flower color and fruit skin. We found 65 copies of *F3′H* in the eucalyptus genome, 35 in longan, 28 in peach, 25 in orange, 26 in Populus, and only 12 in grape and 10 in Arabidopsis, suggesting that the *F3′H* family was significantly expanded in woody plants and a little contracted in herbs. These findings may provide important clues for the mechanism of flavonoid biosynthesis in plants. The gene encoding ANR, which is involved in the biosynthesis of proanthocyanidins (also called condensed tannins), had higher copy numbers (6) in longan than in Arabidopsis (2), orange (1), peach (1), grape (4), and Populus (5), implying that the expanded *ANR* numbers may play a role in proanthocyanidin biosynthesis. Significantly smaller

numbers of the structural genes *PAL*, *CHS*, and *F3′5′H* were detected in longan (6, 14, 3), Arabidopsis (4, 1, 1), orange (4, 15, 4), peach (3, 7, 4), eucalyptus (9, 16, 8), and Populus (5, 12, 2), compared with the higher numbers detected in grape (13, 34, 12). PAL and CHS are involved in the key regulatory step in the branch pathway of phenylpropanoid biosynthesis specific for synthesis of ubiquitous flavonoid pigments [54], and F3′5′H is important for determining flower color [55], which may suggesting that the PAL, CHS, and F3′5′H encoding genes that were discarded in the evolution history of longan, Arabidopsis, orange, peach, eucalyptus, and Populus compared with grape were functionally redundant. Besides the expanded and contracted numbers of structural genes, other structural genes, namely *DHS*, *DHQS*, *SK*, *EPSP*, *CS*, *CM*, *ADT*, *C4H*, *4CL*, *CHI*, *F3H*, *DFR*, and *ANS*, showed little variations in copy numbers among longan, Arabidopsis, orange, peach, grape, poplar, and eucalyptus, which indicated their evolutionary conservation in different plant species. Overall, the expended, contracted, and conserved copy numbers of the 26 selected structural genes among the seven selected plants defined the different characteristics of polyphenol biosynthesis in the different species.

To further understand the functions of the 26 structural genes, we measured their expression levels between primary and secondary metabolism during longan vegetative and reproductive growth (Fig. 3b, Supplemental EXCEL File 7). The PCA showed that all the genes related to the biosynthesis of polyphenols were similarly expressed in leaf, pulp, and pericarp, but their expression levels differed among root, stem, flower bud, flower, young fruit, and seed (Fig. 3b), suggesting these genes may

have tissue-specific roles in longan. Thirteen of the 26 structural genes were found to be expressed in specific tissues, such as root, flower, flower bud, and/or seed (Supplemental EXCEL File 7). For example, two members of the *SDH* family, Cs9g05070.1-D1 and Cs9g05070.1-D5, showed high expression levels during the vegetative and reproductive stages, especially in pulp and pericarp, while the other members of the family were barely detectable, suggesting that Cs9g05070.1-D1 and Cs9g05070.1-D5 may play major roles in the shikimate acid pathway. The six members of the *PAL* family all exhibited low or undetectable expression levels in pulp, two had the highest expression levels in stem, and the other four were strongly expressed in stem, root, leaf, flower, and pericarp. The tissue-specific expression pattern of *PAL* further confirmed that PAL was related to lignin, the structural component of the cell wall in longan [56]. Five of the 14 members of the *CHS* family were barely detectable among the nine samples; among the other members, the highest expression levels were observed for four in seed, three in flower bud, and two in root, suggesting that CHS played important roles in the synthesis of flavonoid pigments in flower bud and seed. The 35 members of the *F3'H* family (Fig. 3c), exhibited different temporal and spatial expression levels (Fig. 3d). Among them, the highest expression levels were observed for one of the members in root, two in stem, five in leaf, eleven in flower bud, three in flower, six in young fruit, three in pericarp, and three in seed; while 11 *F3'H* family members were barely detectable in pericarp, pulp, and seed. For the three members of the *F3'5'H* family, one was detected only in root and one only in flower bud, implying *F3'H* and *F3'5'H* both played major roles in

determining longan flower colors. Proanthocyanidin synthesis involves both LAR and ANR (Fig. 3c). The six *ANR* family members and two of the four *LAR* members were barely detectable in pulp, and all the *ANR* and *LAR* genes were highly expressed in pericarp, and relatively less expressed in seed (Fig. 3d). Previous studies of 12 varieties of Chinese longan fruit have shown that total polyphenols, tannins, and proanthocyanidins were most abundant in pericarp, followed by seed and pulp [57]. The high expression levels of *ANR* and *LAR* in pericarp and seed, and their lowest expression levels in pulp indicated they may determine the tannin composition of longan fruit, further indicating why whole longan fruit is dried for use in sweet desserts and soups for human health [58].

The MYB family of TFs is involved in the regulation of flavonoid biosynthesis [59]. To further investigate the biosynthesis of polyphenols in longan, we compared the numbers of MYB-encoding genes in longan with their numbers in Arabidopsis, orange, peach, and grape. We also investigated their expression levels in longan using the genome and transcriptome data. We detected 94 *R2R3-MYB* genes in longan, which was more than in orange (74) and peach (88), but less than in grape (116), and Arabidopsis (141) (Fig. 4a). A neighbor-joining tree of the *MYB* gene family was constructed (Fig. 4b). The expression profiles of the *MYB* gene family in each tissue were clustered by PCA. The plots showed that the expression profiles in three of the tissues (stem, pericarp, and seed) formed one cluster, while the expression profiles of the other tissues were independently separated, implying that each had a distinct *MYB* expression profile (Fig. 4c). All members of the *MYB* gene family were expressed at

varying levels among the nine vegetative growth and reproductive growth tissues, with some preferentially expressed in specific tissues (Fig. 4d, Supplemental EXCEL File 8). In Arabidopsis, specific *R2R3-MYB* family members, namely *MYB*3 -5, -7, -11, -12, -32, -75, -90, -111, -113, -114, and -123, are known to be involved in regulating the flavonoid pathway [59]. In longan, only four *R2R3-MYB* genes, which are homologs of *AtMYB*4, -12, and -123, were found. In Arabidopsis, *AtMYB4* down-regulated *C4H* and controlled sinapate ester biosynthesis in a UV-dependent manner； *AtMYB12* up-regulated *CHS*, *CHI*, *F3H*, and *F3'H*, and controlled flavonol biosynthesis in all the tissues tested; and *AtMYB123* up-regulated *DNS* and controlled the biosynthesis of proanthocyanidins in the seed coat [59]. In longan, three of the four homologous *R2R3-MYB* genes reached peaks in root, but were undetected or lowly expressed in pericarp, pulp, and seed (Fig. 4d). The tissue-specific expression of these genes indicated they may be required for flavonoid biosynthesis.

Identification and classification of genes encoding NBS-LRR and LRR-RLK

Transcriptome data analysis showed that longan contained a large number of significantly differentially expressed plant pathogen resistance genes. To further investigate the molecular basis for longan pathogen susceptibility, we searched for two classes of resistance genes in the longan genome, those encoding nucleotide binding site-leucine rich repeat (NBS-LRR) proteins and those encoding leucine rich repeat-receptor-like kinases (LRR-RLK). We identified 594 NBS-LRR and 338 LRR-RLK encoding genes, which accounted for approximately 1.51% and 0.86% of the annotated protein-coding genes in longan, respectively. These numbers of NBS-LRR

and LRR-RLK coding genes in the longan genome were more than those in orange (509, 325) [13], grape (341, 234) [10], kiwifruit (110, 259) [16], peach (425, 268) [14], mei (411, 253) [12], and papaya (60, 134) [9], but nearly half that in apple (1035, 477) [11] (Additional file 1: Table S19). *NBS* and *LRR* existed before the divergence of prokaryotes and eukaryotes, but their fusion has been detected only in land plant lineages [60], which are assumed to have originated from a common ancestor. A previous study showed that grape was the oldest among the fruits tested [53]. In this study, the numbers of *NBS-LRR* and *LRR-RLK* genes were either more or less in longan, orange, kiwifruit, peach, papaya, mei, and apple compared with grape. Detail analysis showed that the total number of genes encoding NBS and LRR was not associated with genome expansion or the total number of protein-coding genes in the selected genomes, which is similar to what was found in grass species [60]. Moreover, the NBS- and LRR-encoding genes were significantly more in apple than in the other selected fruits, possibly as a result of a whole-genome wide duplication event in apple [53]. The uneven distribution of NBS-, and LRR-encoding genes on chromosomes was reported previously in Arabidopsis, rice, grapevine, and poplar [61]. These results suggest that changes in the numbers of genes encoding NBS-LRR and LRR-RLK in different species may alter the resistance of these species to different diseases.

The 594 encoded NBS-LRRs in longan were classified into six subgroups based on their protein domains: NBS-LRR (258, 43.43%), coiled-coil-NBS-LRR (150, 25.25%), NBS (122, 20.54%), coiled-coil-NBS (37, 6.23 %), Toll interleukin receptor

(TIR)-NBS-LRR (23, 3.87%), and TIR-NBS (4, 0.67%) (Additional file 1: Table S19). Previous studies have shown that the deduced NBS-LRR proteins can be divided into two subfamilies, TIR and non-TIR proteins based on their N-terminal features [62]. The TIR family of *NBS-LRR* genes probably originated earlier than the non-TIR family [60]. Here, the number of genes encoding the TIR proteins (TIR-NBS-LRR and TIR-NBS) varied from one (kiwifruit) to 288 (apple), and the number of genes encoding the non-TIR proteins was 567 in longan, 415 in orange, 320 in grape, 109 in kiwifruit, 282 in peach, 53 in papaya, and 753 in apple. The ratio of TIR to non-TIR genes was found to differ markedly in different species [62], suggesting ancient origins and subsequent divergence between the two NBS gene types. The distribution of resistance genes in the longan genome and the encoded domains are similar to those of the resistance proteins in other sequenced genomes, as shown in Additional file 1: Table S19. In addition, we noted that allelic variations due to the presence of SNPs in NBS-encoding genes were associated with the phenotypic divergence between resistant ('FY, 'SN1H, 'MQ', 'LDB', and 'JYW') and susceptible ('SX', and 'YTB') longan accessions. Such detailed knowledge of the longan genome will help to accelerate the development of genetic strategies to counter fruit loss caused by diverse pathogens [30].

Conclusions

Here, a draft genome of *D. longan* is presented for the first time. The assembled genome sequence is 471.88 Mb with 273.44-fold coverage obtained by paired-end sequencing. Whole-genome resequencing and analysis of 13 representative cultivated

D. longan accessions revealed the extent of genetic diversity and contributed to trait discovery. Annotation of the protein-coding genes, comparative genomic analysis, and transcriptome analyses provided insights into longan-specific traits, particularly those involved in the biosynthesis of secondary metabolites and pathogen resistance.

Methods

Germplasm genetic resources

An 80-year old *D. longan* 'HHZ' cultivar from the Fujian Agriculture and Forestry University, China, was used for genomic DNA isolation and sequencing. RNA samples from root, leaf, floral bud, flower, young fruit, mature fruit, pericarp, pulp, and seed tissues of the *D. longan* 'SJM' cultivar from the experimental fields of Fujian Academy of Agricultural Science in Putian, Fujian Province, were collected for transcriptome sequencing. Fourteen *D. longan* cultivars, 'HHZ', 'SJM', 'SN1H', 'JYW', 'SX', 'WLL', 'MQ', 'YTB', 'SEY', 'LDB', 'JHLY', 'FY', 'DB', and 'SFB', that originated or are widely grown in Asia and other regions of the world, were collected for resequencing.

DNA extraction, library construction, whole-genome shotgun sequencing and assembly

Whole-genome shotgun sequencing was performed using the Illumina HiSeq 2000 system. Genomic DNA was extracted from fresh mature leaves of the *D. longan* 'HHZ' cultivar using the modified SDS method. DNA sequencing libraries were constructed according to the standard Illumina library preparation protocols. A total of 12 paired-end sequencing libraries, spanning 170, 250, 500, 800, 2,000, 5,000, 10,000, 20,000, and 40,000 bp, were constructed and sequenced on an Illumina HiSeq 2000 system. After stringent filtering and correction steps using K-mer frequency-based methods [21], a total of 121.68 Gb of data were obtained,

and then assembled using SOAPdenovo and SSPACE software [63]. To check the completeness of the assembly, a longan transcriptome assembly comprising 68,925 unigenes [SRA050205] was mapped to the genome assembly using BLAT32 with various sequence homology and coverage parameters. The BUSCO pipeline [27] was also used to check the genome completeness.

Repetitive elements identification

Tandem repeats and interspersed repeats are two main types of repeats found in genomes. Tandem repeats were identified using LTR_FINDER [64] with the default parameters. Interspersed repeats were identified by Repeat Masker (http://www.repeat masker.org/) and RepeatProteinMask using the Repbase library [65] and the *de novo* transposable element library. Identified repeats were then classified into different known classes, as previously described [33].

Gene prediction and annotation

For gene prediction, the scaffolds were first repeat-masked [65]. Then, three *de novo* homology-based and RNA-seq unigenes-based prediction methods, Augustus [66], GENSCAN [67], and GlimmerHMM [68], were used with parameters trained on *Arabidopsis thaliana* and *Carica papaya*. The *de novo* predictions were then merged into a unigene set. For the homology search, translated protein sequences from three sequenced plant genomes (*Glycine max*, *Populus trichocarpa*, and *Vitis vinifera*) were mapped to the longan genome assembly using TBLASTN (E-value cutoff 1×10^{-5}). To extract accurate exon–intron information, the homologous genome sequences were aligned against the matching proteins using GeneWise [69]. Subsequently, the Illumina RNA-seq unigenes sequences [26] were

aligned to the longan genome assembly using BLAT [70] to detect spliced alignments.

Finally, to generate the consensus gene set, the results obtained using the three methods described above were integrated using the GLEAN program [71]. The final gene set contained 39,282 genes. TFs were identified and classified using the TAK program [72]. Non-coding RNAs were predicted and classified, as previously described [73]. Functions of the predicted protein genes were obtained by BLAST searches (E-value cutoff 1×10^{-5}) against the InterproScan [74], GO [75], KEGG [76], SwissProt [77], and TrEMBL databases.

Gene families and phylogenetic analysis

To identify gene families, the translated proteins sequences from *T. cacao*, *C. sinensis*, *A. thaliana*, *C. papaya*, *Populus trichocarpa*, *Glycine max*, *V. vinifera*, *M. acuminata*, *P. persica*, *A.chinensis*, and *M.domestica* genomes were scanned using BLASTP (E-value cutoff 1e−5), and gene family clusters among the different plant species were identified by OrthoMCL [78]. Single-copy families that were represented in all the selected species were alignment using MUSCLE [79]. 4DTv in the 12 species, including longan, were used to construct a phylogenetic tree by MRBAYES [80]. The divergence time was estimated using the MultiDivtime software [79]. Colinearity between *D. longan* and *P. trichocarpa* was computed by SyMAP v3.4 [81]. Subsequently, TF families were identified using the IPR2genomes tool in GreenphylDB v2.0 [82] based on InterPro domains, and gene family expansion and contraction within phylogenetically-related organisms was detected by CAFÉ, a tool for computational analysis of gene family evolution [31].

Resequencing, SNPs, indels, and sequence variations analysis

Paired-end Illumina libraries for 13 *D. longan* cultivars were prepared following the

manufacturer's instructions and sequenced on an Illumina HiSeq 2000 system. After stringent filtering and correction steps, the resulting sequence data were uniquely aligned to the reference longan genome. SNPs, indels, and sequence variations were identified using SOAPsnp (http://soap. genomics.org.cn/ soapsnp.html), SOAPindel [83], and SOAPsv [84].

We used all and high quality SNPs to infer the phylogeography and population structure for *D. longan*. A phylogenetic tree was subsequently generated using the neighbor-joining method implemented in TreeBeST. The bootstrap was set as 1000 replicates.

Population structure was examined primarily via PCA using our own program and model-based clustering algorithms implemented in FRAPPE v1.1 (http://smstaging. stanford. edu/tanglab/software/frappe.html), We increased the pre-defined genetic clusters from K2 to K7 and ran the analysis with 10,000 maximum iterations.

Transcriptome sequencing

Transcriptome sequencing was performed on the Illumina HiSeq 2000 system. Total RNAs from the samples descried above were isolated using a TRIzol Reagent kit (Invitrogen, Carlsbad, CA). cDNA libraries were constructed and sequenced using the Illumina protocols. All the raw reads were first processed to remove the adaptor sequences, low quality reads, and possible contaminations from chloroplast, mitochondrion, and ribosomal DNA. The clean reads were then aligned to the longan genome sequence using TopHat [46] to identify exons and splice junctions *ab initio*. The expression levels of matched genes in each cDNA library were derived and normalized to fragments per kilobase of exon per million fragments mapped. Cluster 3.0 [85] was used to analyze hierarchical clustering of genes. DEGs among different samples were identified using the EBSeq packages [86]. Subsequently, GATK

(http://www.broadinstitute.org/gatk/) with default parameters was used to call SNPs based on the transcript sequence data.

Identification of genes associated with secondary metabolites

We downloaded all the proteins from Arabidopsis, orange, peach, and grape, and identified the genes encoding them using the following methods. First, we collected previously published related genome sequences as the query sequences. We then used TBLASTN (NCBI Blast v2.2.23) [70] to align the query sequences against each genome sequence (E-value cutoff <1e−10). Because many query sequences aligned to the same genomic region, we extracted only the high quality alignments (Query_align_ratio ≥70% and Identity ≥40%). Functional intact genes were confirmed as follows. First, we collected the blast-hits as described above. Then, we extended each of the blast-hits sequences in both the 3′ and 5′ directions along the genome sequences and predicted the gene structure by Genewise (v2.2.0) [69]. Using this approach, we obtained all the pathway genes in longan and the other fruit plants.

Identification of *MYB* genes

We download the annotated *MYB* genes from Arabidopsis, orange, peach, and grape, and applied identification methods that were similar to those described in the 'Identification of genes associated with secondary metabolites' section.

Disease resistance genes analysis

Identification of longan resistance-related genes was based on the most conserved motif structures of plant resistance proteins. Details of the methods used were as described in [30].

Availability of supporting data and materials

The draft genome sequencing project of *D. longan* is registered at NCBI under BioProject [PRJNA305337]. The NCBI SRA database with accession numbers [SRA315202], and the sample Accession were [SRS1272137], [SRS1272138], [SRS1272139], and [SRS1272140]. The *D. longan* 'SJM' transcriptome data is deposited at NCBI under BioProject [PRJNA326792]. Supporting genome assemblies, annotations, supplemental data and custom scripts are hosted in the *GigaScience* GigaDB repository [87].

Declarations

List of abbreviations

ADT: arogenate dehydratase/ prephenate dehydratase; **ANS:** anthocyanidin synthase; **CS:** chorismate synthase; **CM:** chorismate mutase; **C4H:** cinnamate 4-hydroxylase; **CHS:** chalcone synthase; **CHI:** chalcone-flavanone isomerase; **DHS:** 3-deoxy-D-arabino- heptulosonate 7-phosphate synthase; **DHQS:** 3-dehydroquinate synthase; **DFR:** dihydroflavonol 4-reductase; **EPSPS:** 3-phosphoshikimate 1-carboxyvinyltransferase/ 5-enolpyruvylshikimate- 3- phosphate/ EPSP synthase; **F3H:** flavanone 3-hydroxylase**;** **F3′H:** flavonoid 3′-hydroxylase; **F3′5′H:** flavonoid 3′,5′-hydroxylase; **indels:** insertions/ deletions; **LDOX:** leucoanthocyanidin dioxygenase; **LAR:** leucoanthocyanidin reductase; **Mb:** million base; **PCA:** principal component analysis; **PAL:** phenylalanine ammonia lyase; **SNPs:** single nucleotide polymorphisms; **SDH:** bifunctional 3- dehydroquinate dehydratase/ shikimate dehydrogenase; **SK:** shikimate kinase; **4CL:** 4-coumaroyl- coenzyme A ligase.

Consent for publication

Not applicable

Competing interests

The authors declare no competing financial interests.

Funding

This work was funded by the Research Funds for the National Natural Science Foundation of China (31672127, 31572088, 31272149, 31201614, and 31078717), the Science and Technology Plan Major Projects of Fujian Province (2015NZ0002-1), the Natural Science Funds for Distinguished Young Scholar in Fujian Province (2015J06004), the program for New Century Excellent Talents in Fujian Province University (20151104), the Doctoral Program of Higher Education of the Chinese Ministry of Education (20093515110005 and 20123515120008), the Education Department of Fujian Province Science and Technology Project (JA14099), the Program for High-level University Construction of the Fujian Agriculture and Forestry University (612014028), and the Natural Science Funds for Distinguished Young Scholar of the Fujian Agriculture and Forestry University (xjq201405).

Authors' contributions

ZXL, YLL, and YY designed the research; YLL, ZXL, RLL, YKC, CZC, QLT, WHL, LXL, DMZ, MKT, ZHZ, CSZ, and SCL collected the samples and prepared the DNA and RNA. LLY, ZYW, QFL, and YH did the sequencing, processed the raw data, and assembled the sequences. XDF, ZYW, CGZ, JW, and HMY coordinated the project. YLL, ZXL, JMM, LLY, ZYW, QFL, and YH analyzed the data. YLL, and JMM wrote the paper. ZXL, YY, and RKV revised the paper.

Acknowledgments

We thank the following colleagues from the experimental fields of the Fujian Academy of Agricultural Science in Putian for samples.

Additional files

Additional file 1: Tables S1 to S19

Additional file 2: Figures S1 to S7

Supplementary EXCEL File 1: Identification of transcription factors in the *Dimocarpus longan* genome

Supplementary EXCEL File 2: Significantly expanded gene families detected in the *Dimocarpus longan* genome (Viterbi p $\leq$0.05)

Supplementary EXCEL File 3: Significantly contracted gene families detected in the *Dimocarpus longan* genome (Viterbi p $\leq$0.05)

Supplementary EXCEL File 4: SNP analysis of FY, SN1H, MQ, LDB, and JYW cultivars

Supplementary EXCEL File 5: SNP analysis of SX and YTB cultivars

Supplementary EXCEL File 6: Statistics of copy numbers of genes involved in the biosynthesis of polyphenols in different plants

Supplementary EXCEL File 7: Expression levels of genes involved in the biosynthesis of polyphenols in *Dimocarpus longan*

Supplementary EXCEL File 8: MYB genes expressed in nine different tissues of *Dimocarpus longan*

References

1. Lai Z, Chen C, Zeng L, Chen Z. Somatic embryogenesis in longan [*Dimocarpus longan* Lour.]. In: *Somatic Embryogenesis in Woody Plants.* Edited by Jain SM, Gupta P, Newton R, vol. 67: Springer Netherlands; 2000. p.415-431.
2. Luo J, Zhou C-f, Wan Z. Analysis on the development status of lychee industry in Guangdong province in 2010. Guangdong Agricultural

Sciences. 2011; 4:16-8.
3. Mei ZQ, Fu SY, Yu HQ, Yang LQ, Duan CG, Liu XY, Gong S, Fu JJ. Genetic characterization and authentication of *Dimocarpus longan* Lour. using an improved RAPD technique. Genet Mol Res. 2014; 13(1):1447-55.
4. Jiang G, Jiang Y, Yang B, Yu C, Tsao R, Zhang H, Chen F. Structural characteristics and antioxidant activities of oligosaccharides from longan fruit pericarp. Journal of agricultural and food chemistry. 2009; 57(19):9293-98.
5. Chung YC, Lin CC, Chou CC, Hsu CP. The effect of longan seed polyphenols on colorectal carcinoma cells. European journal of clinical investigation. 2010; 40(8):713-21.
6. Prasad KN, Yang B, Shi J, Yu C, Zhao M, Xue S, Jiang Y. Enhanced antioxidant and antityrosinase activities of longan fruit pericarp by ultra-high-pressure-assisted extraction. Journal of pharmaceutical and biomedical analysis. 2010; 51(2):471-77.
7. Lin T, Lin Y, Ishiki K. Genetic diversity of *Dimocarpus longan* in China revealed by AFLP markers and partial rbcL gene sequences. Scientia Horticulturae. 2005; 103(4):489-98.
8. Yonemoto Y, Chowdhury AK, Kato H, Macha MM. Cultivars identification and their genetic relationships in *Dimocarpus longan* subspecies based on RAPD markers. Scientia Horticulturae. 2006; 109(2):147-52.
9. Ming R, Hou S, Feng Y, Yu Q, Dionne-Laporte A, Saw JH, Senin P, Wang W, Ly BV, Lewis KL *et al*.The draft genome of the transgenic tropical fruit tree papaya (*Carica papaya* Linnaeus). Nature. 2008; 452(7190):991-96.
10. Jaillon O, Aury JM, Noel B, Policriti A, Clepet C, Casagrande A, Choisne N, Aubourg S, Vitulo N, Jubin C *et al*. The grapevine genome sequence suggests ancestral hexaploidization in major angiosperm phyla. Nature. 2007; 449 (7161):463-67.
11. Velasco R, Zharkikh A, Affourtit J, Dhingra A, Cestaro A, Kalyanaraman A, Fontana P, Bhatnagar SK, Troggio M, Pruss D *et al*. The genome of the domesticated apple (*Malus x domestica* Borkh.). Nature genetics. 2010; 42(10):833-39.
12. Zhang Q, Chen W, Sun L, Zhao F, Huang B, Yang W, Tao Y, Wang J, Yuan Z, Fan G *et al*. The genome of *Prunus mume*. Nature communications. 2012; 3:1318.
13. Xu Q, Chen LL, Ruan X, Chen D, Zhu A, Chen C, Bertrand D, Jiao WB, Hao BH, Lyon MP *et al*. The draft genome of sweet orange (*Citrus sinensis*). Nature genetics. 2013; 45(1):59-66.
14. Verde I, Abbott AG, Scalabrin S, Jung S, Shu S, Marroni F, Zhebentyayeva T, Dettori MT, Grimwood J, Cattonaro F *et al*. The high-quality draft genome of peach (*Prunus persica*) identifies unique patterns of genetic diversity, domestication and genome evolution. Nature genetics. 2013; 45(5):487-94.
15. Wu J, Wang Z, Shi Z, Zhang S, Ming R, Zhu S, Khan MA, Tao S, Korban SS, Wang H *et al*. The genome of the pear (*Pyrus bretschneideri* Rehd.). Genome Res. 2013; 23(2):396-408.

16. Huang S, Ding J, Deng D, Tang W, Sun H, Liu D, Zhang L, Niu X, Zhang X, Meng M *et al*. Draft genome of the kiwifruit *Actinidia chinensis*. Nature communications. 2013; 4:2640.
17. Ming R, VanBuren R, Wai CM, Tang H, Schatz MC, Bowers JE, Lyons E, Wang M-L, Chen J, Biggers E *et al*. The pineapple genome and the evolution of CAM photosynthesis. Nature genetics. 2015; 47(12): 1435-42.
18. D'Hont A, Denoeud F, Aury JM, Baurens FC, Carreel F, Garsmeur O, Noel B, Bocs S, Droc G, Rouard M *et al*. The banana (*Musa acuminata*) genome and the evolution of monocotyledonous plants. Nature. 2012; 488(7410):213-7.
19. Ma Q, Feng K, Yang W, Chen Y, Yu F, Yin T. Identification and characterization of nucleotide variations in the genome of *Ziziphus jujuba* (Rhamnaceae) by next generation sequencing. Mol Biol Rep. 2014; 41(5): 3219-23.
20. Shulaev V, Sargent DJ, Crowhurst RN, Mockler TC, Folkerts O, Delcher AL, Jaiswal P, Mockaitis K, Liston A, Mane SP *et al*. The genome of woodland strawberry (*Fragaria vesca*). Nature genetics. 2011; 43(2):109-16.
21. Li R, Fan W, Tian G, Zhu H, He L, Cai J, Huang Q, Cai Q, Li B, Bai Y *et al*. The sequence and de novo assembly of the giant panda genome. Nature. 2010; 463(7279):311-7.
22. Sun L, Zhang Q, Xu Z, Yang W, Guo Y, Lu J, Pan H, Cheng T, Cai M. Genome-wide DNA polymorphisms in two cultivars of mei (*Prunus mume* sieb. et zucc.). BMC Genet. 2013; 14:98.
23. Brunner AM, Busov VB, Strauss SH. Poplar genome sequence: functional genomics in an ecologically dominant plant species. Trends in plant science. 2004; 9(1):49-56.
24. Li R, Zhu H, Ruan J, Qian W, Fang X, Shi Z, Li Y, Li S, Shan G, Kristiansen K *et al*. De novo assembly of human genomes with massively parallel short read sequencing. Genome Res. 2010; 20(2):265-72.
25. Du H, Hu H, Meng Y, Zheng W, Ling F, Wang J, Zhang X, Nie Q, Wang X. The correlation coefficient of GC content of the genome-wide genes is positively correlated with animal evolutionary relationships. FEBS Lett. 2010; 584(18):3990-4.
26. Lai Z, Lin Y. Analysis of the global transcriptome of longan (*Dimocarpus longan* Lour.) embryogenic callus using Illumina paired-end sequencing. BMC Genomics. 2013; 14:561.
27. Simao FA, Waterhouse RM, Ioannidis P, Kriventseva EV, Zdobnov EM. BUSCO: assessing genome assembly and annotation completeness with single-copy orthologs. Bioinformatics. 2015; 31(19):3210-2.
28. Lee H, Golicz AA, Bayer PE, Jiao Y, Tang H, Paterson AH, Sablok G, Krishnaraj RR, Chan CK, Batley J *et al*. The Genome of a Southern Hemisphere Seagrass Species (*Zostera muelleri*). Plant Physiol. 2016; 172(1): 272-83.
29. Meyers BC, Tingey SV, Morgante M. Abundance, distribution, and transcriptional activity of repetitive elements in the maize genome. Genome

Res. 2001; 11(10):1660-76.
30. Velasco R, Zharkikh A, Troggio M, Cartwright DA, Cestaro A, Pruss D, Pindo M, Fitzgerald LM, Vezzulli S, Reid J *et al*. A high quality draft consensus sequence of the genome of a heterozygous grapevine variety. PLoS One. 2007; 2(12):e1326.
31. De Bie T, Cristianini N, Demuth JP, Hahn MW. CAFE: a computational tool for the study of gene family evolution. Bioinformatics. 2006; 22(10):1269-71.
32. Guo S, Zhang J, Sun H, Salse J, Lucas WJ, Zhang H, Zheng Y, Mao L, Ren Y, Wang Z *et al*. The draft genome of watermelon (*Citrullus lanatus*) and resequencing of 20 diverse accessions. Nature genetics. 2013; 45(1):51-8.
33. Huang S, Li R, Zhang Z, Li L, Gu X, Fan W, Lucas WJ, Wang X, Xie B, Ni P *et al*. The genome of the cucumber, *Cucumis sativus* L. Nature genetics. 2009; 41(12):1275-81.
34. Wang B, Tan HW, Fang W, Meinhardt LW, Mischke S, Matsumoto T, Zhang D. Developing single nucleotide polymorphism (SNP) markers from transcriptome sequences for identification of longan (*Dimocarpus longan*) germplasm. Horticulture research. 2015; 2:14065.
35. Zhu J, Pan L, Qin X, Peng H, Wang Y, Hang Z. Analysis on genetic relations in different ecotypes of longan (*Dimocarpus longan* Lour.) germplasm resources by ISSR markers. Journal of Plant Genetic Resources. 2013; (01):65-9.
36. Zhong F, Pan D, Guo Z, Lin L, Li K. RAPD Analysis of Longan Germplasm Resources. Chinese agricultural science bulletin. 2007; (07):558-63.
37. Tang H, Peng J, Wang P, Risch NJ. Estimation of individual admixture: analytical and study design considerations. Genetic epidemiology. 2005; 28(4): 289-301.
38. Peng J, Xie L, Xu B, Dang J, Li Y, Lu Z, Zhang S, Yu Z, Bai X, Cai Z. Study on Biological Characters of 'Sijihua' Longan. In: III International Symposium on Longan, Lychee, and other Fruit Trees in Sapindaceae Family 863: 2008; p.249-258.
39. Wang L, Zhao S, Gu C, Zhou Y, Zhou H, Ma J, Cheng J, Han Y. Deep RNA-Seq uncovers the peach transcriptome landscape. Plant molecular biology. 2013; 83(4-5):365-77.
40. Tong C, Wang X, Yu J, Wu J, Li W, Huang J, Dong C, Hua W, Liu S. Comprehensive analysis of RNA-seq data reveals the complexity of the transcriptome in *Brassica rapa*. BMC Genomics. 2013; 14:689.
41. Liu J, Jung C, Xu J, Wang H, Deng S, Bernad L, Arenas-Huertero C, Chua NH. Genome-wide analysis uncovers regulation of long intergenic noncoding RNAs in Arabidopsis. Plant Cell. 2012; 24(11):4333-45.
42. Wang XJ, Gaasterland T, Chua NH. Genome-wide prediction and identification of *cis*-natural antisense transcripts in *Arabidopsis thaliana*. Genome Biol. 2005; 6(4):R30.
43. Jia T, Wei D, Meng S, Allan AC, Zeng L. Identification of regulatory genes implicated in continuous flowering of longan (*Dimocarpus longan* L.). PLoS

One. 2014; 9(12):e114568.
44. Lin Y, Lai Z. Comparative analysis reveals dynamic changes in miRNAs and their targets and expression during somatic embryogenesis in longan (*Dimocarpus longan* Lour.). PLoS One. 2013; 8(4):e60337.
45. Lin CC, Chung YC, Hsu CP. Potential roles of longan flower and seed extracts for anti-cancer. World Journal of Experimental Medicine. 2012; 2(4):78-85.
46. Trapnell C, Pachter L, Salzberg SL. TopHat: discovering splice junctions with RNA-Seq. Bioinformatics. 2009; 25(9):1105-11.
47. Clark TA, Schweitzer AC, Chen TX, Staples MK, Lu G, Wang H, Williams A, Blume JE. Discovery of tissue-specific exons using comprehensive human exon microarrays. Genome Biol. 2007; 8(4):R64.
48. Vitulo N, Forcato C, Carpinelli EC, Telatin A, Campagna D, D'Angelo M, Zimbello R, Corso M, Vannozzi A, Bonghi C *et al*. A deep survey of alternative splicing in grape reveals changes in the splicing machinery related to tissue, stress condition and genotype. BMC Plant Biol. 2014; 14:99.
49. Wang BB, Brendel V. Genomewide comparative analysis of alternative splicing in plants. Proceedings of the National Academy of Sciences of the United States of America. 2006; 103(18):7175-80.
50. Filichkin SA, Priest HD, Givan SA, Shen R, Bryant DW, Fox SE, Wong WK, Mockler TC. Genome-wide mapping of alternative splicing in Arabidopsis thaliana. Genome Res. 2010; 20(1):45-58.
51. Potenza E, Racchi ML, Sterck L, Coller E, Asquini E, Tosatto SC, Velasco R, Van de Peer Y, Cestaro A. Exploration of alternative splicing events in ten different grapevine cultivars. BMC Genomics. 2015; 16:706.
52. Reddy AS, Marquez Y, Kalyna M, Barta A. Complexity of the alternative splicing landscape in plants. Plant Cell. 2013; 25(10):3657-83.
53. Michael TP, VanBuren R. Progress, challenges and the future of crop genomes. Curr Opin Plant Biol. 2015; 24:71-81.
54. Assis JS, Maldonado R, Muñoz T, Escribano MaI, Merodio C. Effect of high carbon dioxide concentration on PAL activity and phenolic contents in ripening cherimoya fruit. Postharvest Biology and Technology. 2001; 23(1): 33-9.
55. Togami J, Tamura M, Ishiguro K, Hirose C, Okuhara H, Ueyama Y, Nakamura N, Yonekura-Sakakibara K, Fukuchi-Mizutani M, Suzuki K-i *et al*. Molecular characterization of the flavonoid biosynthesis of *Verbena hybrida* and the functional analysis of verbena and *Clitoria ternatea* F3′5′H genes in transgenic verbena. Plant Biotechnology. 2006; 23(1):5-11.
56. Zhang X, Gou M, Liu CJ. Arabidopsis Kelch repeat F-box proteins regulate phenylpropanoid biosynthesis via controlling the turnover of phenylalanine ammonia-lyase. Plant Cell. 2013; 25(12):4994-5010.
57. He N, Wang Z, Yang C, Lu Y, Sun D, Wang Y, Shao W, Li Q. Isolation and identification of polyphenolic compounds in longan pericarp. Separation and Purification Technology. 2009; 70(2):219-24.
58. Tseng HC, Wu WT, Huang HS, Wu MC. Antimicrobial activities of various

fractions of longan (*Dimocarpus longan* Lour. Fen Ke) seed extract. International journal of food sciences and nutrition. 2014; 65(5): 589-93.

59. Dubos C, Stracke R, Grotewold E, Weisshaar B, Martin C, Lepiniec L. MYB transcription factors in Arabidopsis. Trends in plant science. 2010; 15(10): 573-81.
60. Yue JX, Meyers BC, Chen JQ, Tian D, Yang S. Tracing the origin and evolutionary history of plant nucleotide-binding site-leucine-rich repeat (NBS-LRR) genes. New Phytol. 2012; 193(4):1049-63.
61. Li J, Ding J, Zhang W, Zhang Y, Tang P, Chen JQ, Tian D, Yang S. Unique evolutionary pattern of numbers of gramineous NBS-LRR genes. Mol Genet Genomics. 2010; 283(5):427-38.
62. Yang S, Zhang X, Yue JX, Tian D, Chen JQ. Recent duplications dominate NBS-encoding gene expansion in two woody species. Mol Genet Genomics. 2008; 280(3):187-98.
63. Boetzer M, Henkel CV, Jansen HJ, Butler D, Pirovano W. Scaffolding pre-assembled contigs using SSPACE. Bioinformatics. 2011; 27(4):578-9.
64. Xu Z, Wang H. LTR_FINDER: an efficient tool for the prediction of full-length LTR retrotransposons. Nucleic acids research. 2007; 35(Web Server issue):W265-8.
65. Jurka J, Kapitonov VV, Pavlicek A, Klonowski P, Kohany O, Walichiewicz J. Repbase Update, a database of eukaryotic repetitive elements. Cytogenetic and genome research. 2005; 110(1-4):462-7.
66. Stanke M, Keller O, Gunduz I, Hayes A, Waack S, Morgenstern B. AUGUSTUS: ab initio prediction of alternative transcripts. Nucleic acids research. 2006; 34(Web Server issue):W435-9.
67. Salamov AA, Solovyev VV. Ab initio gene finding in Drosophila genomic DNA. Genome Res. 2000; 10(4):516-22.
68. Majoros WH, Pertea M, Salzberg SL. TigrScan and GlimmerHMM: two open source ab initio eukaryotic gene-finders. Bioinformatics. 2004; 20(16): 2878-9.
69. Birney E, Clamp M, Durbin R. GeneWise and Genomewise. Genome Res. 2004;14(5):988-95.
70. Kent WJ. BLAT--the BLAST-like alignment tool. Genome Res. 2002; 12(4): 656-64.
71. Elsik CG, Mackey AJ, Reese JT, Milshina NV, Roos DS, Weinstock GM: Creating a honey bee consensus gene set. Genome Biol. 2007; 8(1):R13.
72. Zheng Y, Jiao C, Sun H, Rosli HG, Pombo MA, Zhang P, Banf M, Dai X, Martin GB, Giovannoni JJ *et al*. iTAK: A program for genome-wide prediction and classification of plant transcription factors, transcriptional regulators, and protein kinases. Mol Plant. 2016; 9(12):1667-70.
73. Varshney RK, Song C, Saxena RK, Azam S, Yu S, Sharpe AG, Cannon S, Baek J, Rosen BD, Tar'an B *et al*. Draft genome sequence of chickpea (*Cicer arietinum*) provides a resource for trait improvement. Nature biotechnology. 2013; 31(3):240-6.

74. Zdobnov EM, Apweiler R. InterProScan--an integration platform for the signature-recognition methods in InterPro. Bioinformatics. 2001; 17(9): 847-8.
75. Ashburner M, Ball CA, Blake JA, Botstein D, Butler H, Cherry JM, Davis AP, Dolinski K, Dwight SS, Eppig JT *et al*. Gene ontology: tool for the unification of biology. The Gene Ontology consortium. Nature genetics. 2000; 25(1):25-9.
76. Kanehisa M, Goto S. KEGG: kyoto encyclopedia of genes and genomes. Nucleic acids research. 2000; 28(1):27-30.
77. Bairoch A, Apweiler R. The SWISS-PROT protein sequence database and its supplement TrEMBL in 2000. Nucleic acids research. 2000; 28(1):45-8.
78. Li L, Stoeckert CJ, Jr., Roos DS. OrthoMCL: identification of ortholog groups for eukaryotic genomes. Genome Res. 2003; 13(9):2178-89.
79. Edgar RC. MUSCLE: multiple sequence alignment with high accuracy and high throughput. Nucleic acids research. 2004; 32(5):1792-7.
80. Huelsenbeck JP, Ronquist F. MRBAYES: Bayesian inference of phylogenetic trees. Bioinformatics. 2001; 17(8):754-5.
81. Soderlund C, Bomhoff M, Nelson WM. SyMAP v3.4: a turnkey synteny system with application to plant genomes. Nucleic acids research. 2011; 39(10):e68.
82. Rouard M, Guignon V, Aluome C, Laporte MA, Droc G, Walde C, Zmasek CM, Perin C, Conte MG. GreenPhylDB v2.0. comparative and functional genomics in plants. Nucleic acids research. 2011; 39 (Database issue): D1095-102.
83. Li S, Li R, Li H, Lu J, Li Y, Bolund L, Schierup MH, Wang J. SOAPindel: efficient identification of indels from short paired reads. Genome Res. 2013; 23(1):195-200.
84. Li Y, Zheng H, Luo R, Wu H, Zhu H, Li R, Cao H, Wu B, Huang S, Shao H *et al*. Structural variation in two human genomes mapped at single-nucleotide resolution by whole genome de novo assembly. Nature biotechnology. 2011; 29(8):723-30.
85. de Hoon MJ, Imoto S, Nolan J, Miyano S. Open source clustering software. Bioinformatics. 2004; 20(9):1453-4.
86. Leng N, Dawson JA, Thomson JA, Ruotti V, Rissman AI, Smits BM, Haag JD, Gould MN, Stewart RM, Kendziorski C. EBSeq: an empirical Bayes hierarchical model for inference in RNA-seq experiments. Bioinformatics. 2013; 29(8):1035-43.
87 Lin Y, Min J, Lai R; Wu Z, Chen Y, Yu L, Cheng C, Jin Y, Tian Q, Liu Q, Liu W, Zhang C, Lin L, Hu Y, Zhang D, Thu M, Zhang Z, Liu S, Zhong C, Fang X, Wang J, Yang H, Varshney RK, Yin Y, Lai Z (2017): Supporting data for "Genome-wide sequencing of longan (Dimocarpus longan Lour.) provides insights into molecular basis of its polyphenol-rich characteristics". *GigaScience Database*. http://dx.doi.org/10.5524/100276

Table Click here to download Table Tables-12.9.doc

Tables

Table 1 *D. longan* genome assembly

	Contig		Scaffold	
	Size(bp)	Number	Size(bp)	Number
N90	6,457	18,861	122,626	983
N80	11,286	13,434	197,247	668
N70	15,938	9,933	283,489	459
N60	20,685	7,339	396,999	309
N50	26,035	5,306	566,629	204
Longest	173,288		6,942,318	
Total size	471,874,380		495,332,425	
Total number(>=200bp)		51,392		17,367
Total number(>=2Kb)		27,296		2,282

Table 2 Statistics and comparison of the *D. longan* assembly to other twelve genomes. Dl, *Dimocarpus longan;* Cs, *Citrus sinensis;* Cc, *Citrus Clementina;* Cp, *Carica papaya;* Ac, *Actinidia chinensis;* Md, *Malus domestica;* Pp, *Prunus persica*; Pb, *Pyrus bretschneideri*; Vv, *Vitis vinifera;* Ac, *Ananas comosus (L.) Merr.*; Zj, *Ziziphus jujuba Mill.*; Mn, *Morus notabilis;* Tc, *Theobroma cacao*.

	Dl	Cs	Cc	Cp	Ac	Md	Pp	Pb	Vv	Ac	Zj	Mn	Tc
Chromosome number (2*n*)	30	18	18	18	58	34	16	34	38	50	24	14	20
Estimate of genome size (Mb)	445	367	370	372	758	742.3	265	527	475	526	444	357	430
Sequence Coverage	273.43	214	7	NA	140	16.9	8.47	194	8.4	400	390	236	16.7
Assembled (Mb)	471.88	320	301	271	616.1	603.9	226.6	512	487	382	437.65	330	326.9
Assembling represent percentage of genome (%)	106.4	87.30	81.4	75	81	81.3	85.50	97.10	102.5	73	98.60	92.4	76
N50 length of contig (Kb)	26.03	49.89	NA	NA	58.9	16.17	294	35.7	65.9	126.5	33.9	34.4	19.8
N50 length of scaffolds (Mb)	0.56662	1.69	NA	NA	0.646	NA	4	0.54	2	11.8	0.3	0.39	0.4738
GC content (%)	33.7	34.06	NA	35.3	35.20	NA	NA	NA	35	33	33.41	35	NA
Repeat content (%)	52.87	20	NA	51.90	36	67.4	29.60	53.10	41.40	38.30	49.49	38.8	25.70
Number of gene models	31,007	29,445	24,533	24,746	39,040	57,386	27,852	42,812	30,434	27,024	32,808	27,085	28,798

NA, no available.

Figure 1 Phylogenetic and evolutionary analysis of the longan genome
Click here to download Figure Figure 1 Phylogenetic and evolutionary analysis .doc

Figure 1 Phylogenetic and evolutionary analysis of the longan genome. (**a**) Molecular phylogenetic analysis based on single-copy genes shared among orange, papaya, Arabidopsis, cacao, poplar, banana, grape, soybean, apple, peach, kiwifruit, and banana from genome data. (**b**) Comparison of the number of gene families in eleven plant species, such as *T. cacao*, *A. thaliana*, *C. sinensis*, *C. papaya*, *P. trichocarpa* , *G. max*, *V. vinifera*, *M. acuminate*, *D. longan*, *P. persica*, *A. chinensis*, and *M. domestica.* (**c**) Distribution of 4DTv distance between syntenic gene pairs among banana, peach, orange, Arabidopsis and grape. (**d**) Distribution of gene families among *D. longan*, *C. sinensis*, *C. papaya*, *V. vinifera*, and *P. persica*. Homologous genes in longan, orange, papaya, grape, and peach were clustered to gene families. The numbers of gene families are indicated for each species and species intersection.

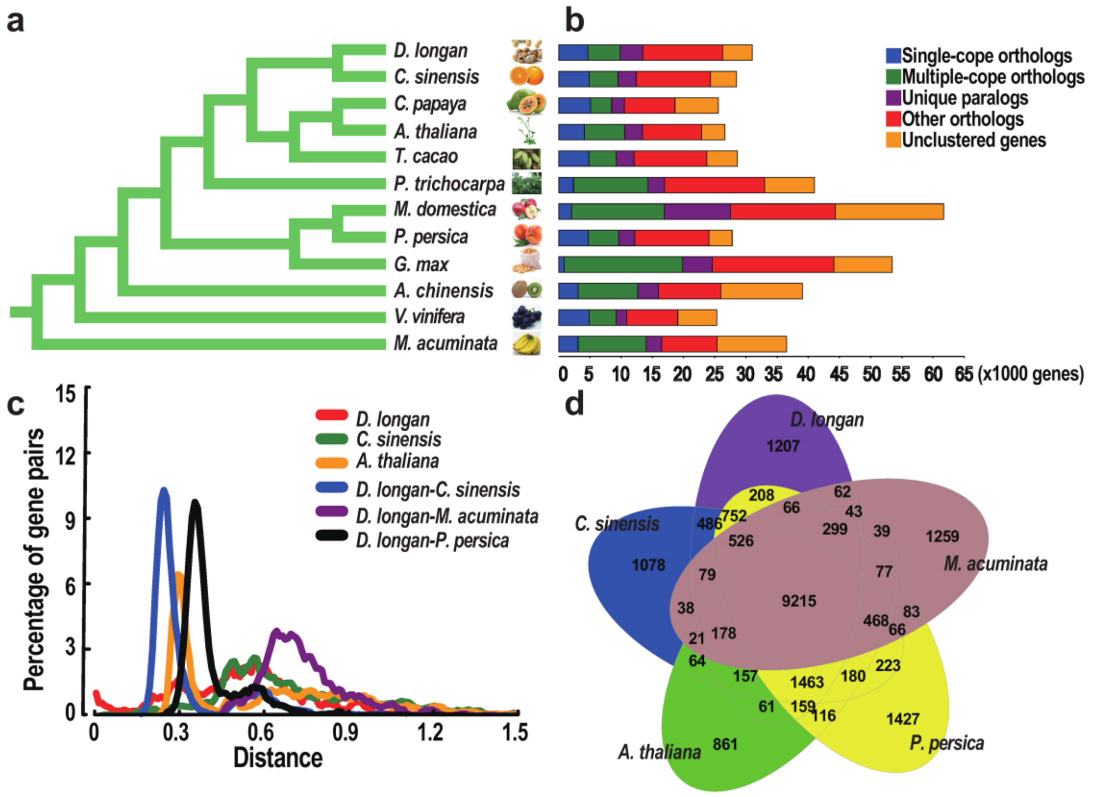

Figure 2 Genetic diversity and population structure of longan accessions.
Click here to download Figure Figure 2 Genetic diversity and population structure.doc

Figure 2 Genetic diversity and population structure of longan accessions. (**a**) Neighbor- joining tree of the 13 longan accessions on the basis of all SNPs. (**b**) PCA of the 13 longan accessions using SNPs as markers. Different colors represent for different longan accession. HHZ, DB, JYW, LDB, WLL, SN1H, YTB, SEY, JHLY, and SX, are clustered together, FY (Quanzhou, China), SJM (South-East Asia), and MQ (Thailand) showed a clear separation. (**c**) Population structure of longan accessions. The distribution of the accessions to different populations is indicated by different color. Each accession is represented by a vertical bar. Numbers on the x-axis show represents the K number, and the y-axis shows the different accession.

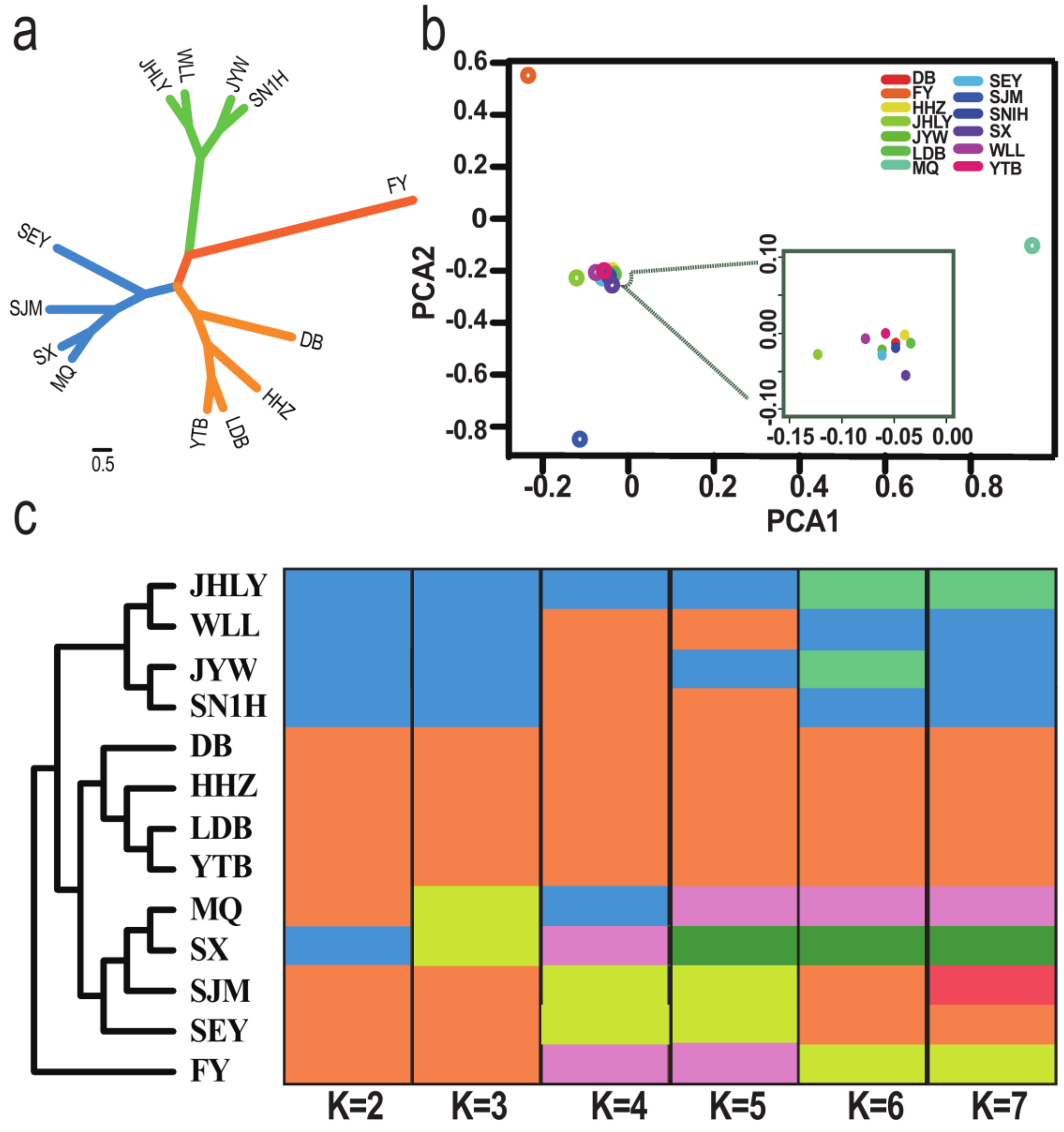

Figure 3 Simplified diagram of polyphenols biosynthetic pathway. (**a**) Simplified diagram of polyphenols biosynthetic pathway. Numbers in brackets represent genes' copy number. (**b**) PCA scatter plot of 9 samples using genes related to polyphenols biosynthetic pathway. (**c**) Neighbor- joining tree of the F3'H, ANR, and LAR from longan, peach, orange, Arabidopsis and grape. (**d**) Cluster analysis of expression profiles of *F3'H*, *ANR*, and *LAR*. The bar represents the scale of relative expression levels of genes, and colors indicate relative signal intensities of genes. Each column represents a sample, and each row represents a single gene.

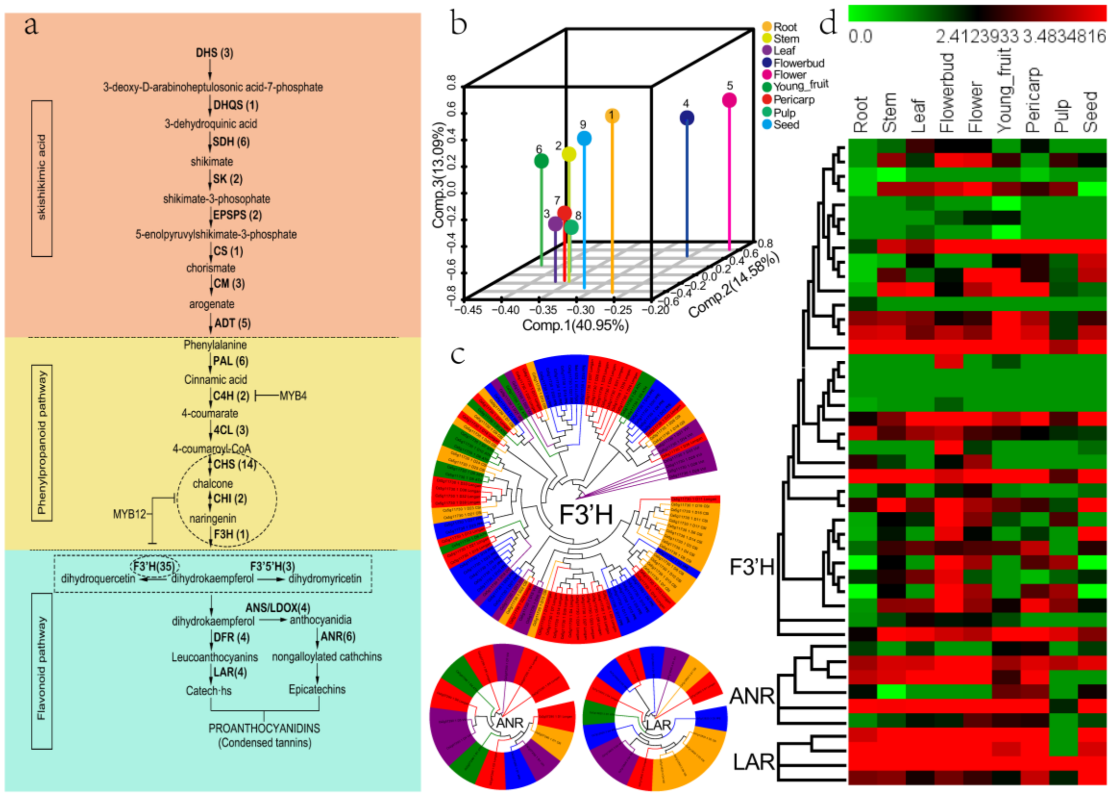

Click here to download Figure Figure 4 The MYB transcription factor in longan genome.doc

Figure 4 The MYB transcription factor in longan genome. (**a**) Numbers of the members in the three different MYB classes in Arabidopsis, orange, longan, peach, and grape. (**b**) Neighbor- joining tree of the *MYB* gene family. (**c**) PCA scatter plot of 9 samples using 94 R2R3-MYB genes. (**d**) Cluster analysis of expression profiles of MYB transcription factor. The bar represents the scale of relative expression levels of genes, and colors indicate relative signal intensities of genes. Each column represents a sample, and each row represents a single gene.

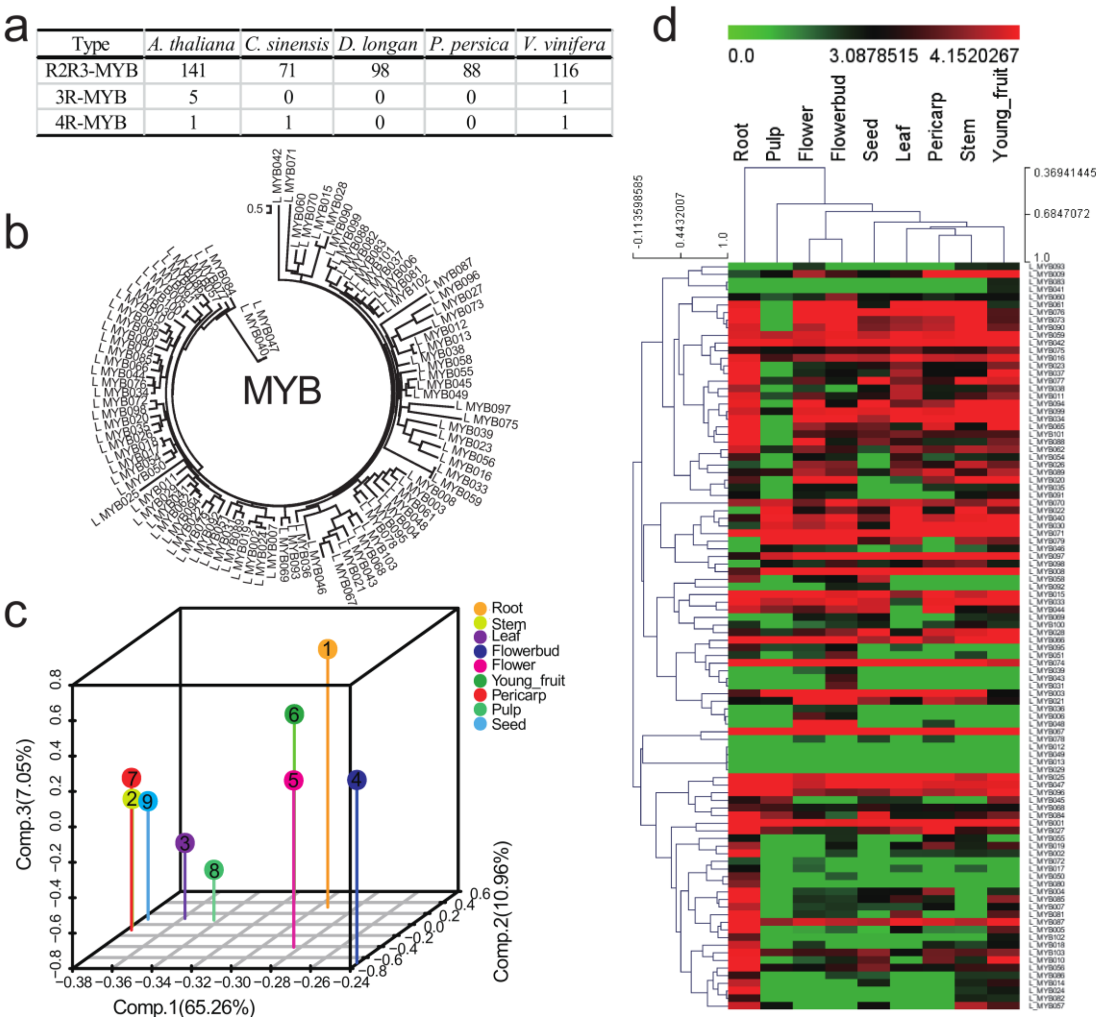

Type	*A. thaliana*	*C. sinensis*	*D. longan*	*P. persica*	*V. vinifera*
R2R3-MYB	141	71	98	88	116
3R-MYB	5	0	0	0	1
4R-MYB	1	1	0	0	1

Click here to access/download
Supplementary Material
Additional file 1-1.17.doc

Click here to access/download
Supplementary Material
Additional file 2-12.9.doc

Supplementary EXCEL file 1- Identification of tanscription factors
in the Dimocarpus longan genome

Click here to access/download
Supplementary Material
Supplementary EXCEL file 1.xls

Supplementary EXCEL file 2 Significantly expanded gene families detected in the Dimocarpus longan genome (Viterbi p ≤0.05)

Click here to access/download
Supplementary Material
Supplementary EXCEL file 2 .xls

Supplementary EXCEL file 3 Significantly contracted gene families detected in the Dimocarpus longan genome (Viterbi p ≤0.05)

Click here to access/download
Supplementary Material
Supplementary EXCEL file 3.xls

Supplementary EXCEL file 4 SNP analysis of FY, SN1H, MQ, LDB, and JYW cultivars

Click here to access/download
Supplementary Material
Supplementary EXCEL file 4.xls

Supplementary EXCEL file 5 SNP analysis of SX and YTB cultivars

Click here to access/download
Supplementary Material
Supplementary EXCEL file 5.xls

Supplementary EXCEL file 6 Statistics of copy numbers of genes involved in the biosynthesis of polyphenols in different plants

Click here to access/download
Supplementary Material
Supplementary EXCEL file 6.xls

Supplementary EXCEL file 7 Expression levels of genes involved in the biosynthesis of polyphenols in Dimocarpus longan

Click here to access/download

Supplementary Material

Supplementary EXCEL file 7.xls

Supplementary EXCEL file 8 MYB genes expressed in nine different tissues of Dimocarpus longan

Click here to access/download
Supplementary Material
Supplementary EXCEL file 8.xls
