# Supplementary material for: Genome-wide sequencing of longan (Dimocarpus longan Lour.) provides insights into molecular basis of its polyphenol-rich characteristics
Source: Gigascience. 2017 Mar 28;6(5):1–14. doi: 10.1093/gigascience/gix023 (PMC5467034; doi:10.1093/gigascience/gix023)
Supplement: Additional file 1: — Tables S1 to S19 [file gix023_Additional_file_1-1.17.doc]

**Table S 1. Illumina sequence raw data of 12 genome shotgun libraries with different fragment lengths in the *Dimocarpus longan* genome assembly and their respective sequence coverage levels in the final release.**

| Pair-end Libraries | Insert Size | Total Data(Gb) | Read length(bp) | Sequence Depth(X) | Physical  Depth(X) |
| --- | --- | --- | --- | --- | --- |
|  |  |  |  |  |  |
| Solexa Reads | 170bp | 18.83 | 100 | 42.30 | 35.96 |
|  | 250bp | 20.95 | 150 | 47.07 | 39.22 |
|  | 500bp | 18.35 | 100 | 41.24 | 103.10 |
|  | 800bp | 31.10 | 100 | 69.88 | 279.50 |
|  | 2kb | 36.25 | 49 | 81.46 | 1,662.41 |
|  | 5kb | 32.32 | 49 | 72.64 | 3,705.97 |
|  | 10kb | 52.13 | 49 | 117.14 | 11,952.96 |
|  | 20kb | 52.64 | 49 | 118.29 | 24,141.02 |
|  | 40kb | 54.28 | 49 | 121.99 | 49,791.02 |
|  | - | 316.84 | - | 712.00 | 91,711.16 |

Note：*D.longan* genome was estimated to be 445 Mb.

**Table S 2. Summary statistics of the output of the whole genome shotgun assembly after stringent filtering and correction steps**

| Pair-end Libraries | Insert Size | Total Data(Gb) | Read length(bp) | Sequence Depth(X) | Physical Depth(X) |
| --- | --- | --- | --- | --- | --- |
|  |  |  |  |  |  |
| Solexa  Reads | 170bp | 17.26 | 100 | 38.79 | 32.97 |
|  | 250bp | 18.65 | 150 | 41.90 | 34.92 |
|  | 500bp | 16.70 | 100 | 37.54 | 93.84 |
|  | 800bp | 24.23 | 100 | 54.44 | 217.76 |
|  | 2kb | 11.56 | 49 | 25.98 | 530.27 |
|  | 5kb | 10.63 | 49 | 23.90 | 1,219.13 |
|  | 10kb | 10.93 | 49 | 24.57 | 2,507.14 |
|  | 20kb | 5.08 | 49 | 11.41 | 2,328.16 |
|  | 40kb | 6.64 | 49 | 14.92 | 6,088.98 |
|  | --- | 121.68 | --- | 273.44 | 13,053.17 |

Note：*D.longan* genome was estimated to be 445 Mb.

**Table S 3. 17-mer statistics for longan genomes.**

| K | K-mer_num | Peak_depth | Genome Size (bp) | Used Bases (bp) | Used Reads | X |
| --- | --- | --- | --- | --- | --- | --- |
| 17 | 28,496,811,588 | 64 | 445,262,681 | 33,924,775,700 | 339,247,757 | 76.19 |

**Table S 4. Transcriptome mapping results for longan genome.**

| Dataset | Number | Total | Bases covered by assembly  (%) | Sequences Covered by Assembly  (%) | With >90% Sequence in one Scaffold | | With >50% Sequence in one Scaffold | |
| --- | --- | --- | --- | --- | --- | --- | --- | --- |
|  |  | Length (bp) |  |  | Number | Percent (%) | Number | Percent (%) |
| >0bp | 68,298 | 62,150,618 | 95.56 | 97.55 | 59,209 | 86.69 | 65,617 | 96.07 |
| >200bp | 68,140 | 62,122,548 | 95.56 | 97.55 | 59,078 | 86.70 | 65,460 | 96.07 |
| >500bp | 34,086 | 51,887,064 | 95.64 | 98.81 | 30,313 | 88.93 | 32,970 | 96.73 |
| >1000bp | 21,257 | 42,693,219 | 95.56 | 99.15 | 18,749 | 88.20 | 20,584 | 96.83 |

Note: The longan transcriptome contain 96,251 scaffolds (≥100) (SRA050205). Of the 96,251 scaffolds (≥100), 97.55 % were identified in the assembly, suggesting that most coding regions were well represented in the assembly.

**Table S 5 The result of assessing genome assembly completeness using BUSCO**

| #BUSCO was run in mode: genome | | |
| --- | --- | --- |
| Summarized benchmarks in BUSCO notation: | | |
| C:94%[D:30%],F:1.6%,M:4.1%,n:956 | | |
| Representing: |  |  |
| 900 Complete Single-Copy BUSCOs | | |
| 288 Complete Duplicated BUSCOs | | |
| 16 Fragmented BUSCOs | |  |
| 40 Missing BUSCOs | |  |
| 956 Total BUSCO groups searched | | |

**Table S 6. Statistics of repetitive elements.**

| Type | Repeat Size(bp) | % of genome |
| --- | --- | --- |
| TRF | 29,699,180 | 6.00 |
| RepeatMasker | 51,934,073 | 10.48 |
| RepeatProteinMask | 54,351,106 | 10.97 |
| De novo | 245,138,462 | 49.49 |
| Total | 261,875,303 | 52.87 |

**Table S 7. Statistics of repetitive elements.**

|  | RepBase TEs | | TE Proteins | | De novo | | Combined TEs | |
| --- | --- | --- | --- | --- | --- | --- | --- | --- |
|  | Length | %in Genome | Length (bp) | % in Genome | Length | % in Genome | Length | % in Genome |
|  | (bp) |  |  |  | (bp) |  | (bp) |  |
| DNA | 5,885,653 | 1.19 | 5,739,472 | 1.16 | 33,782,884 | 6.82 | 37,578,428 | 7.59 |
| LINE | 2,021,098 | 0.41 | 3,700,588 | 0.75 | 9,891,161 | 2.00 | 12,022,595 | 2.43 |
| SINE | 20,657 | 0.00 | 0 | 0.00 | 169,304 | 0.03 | 188,578 | 0.04 |
| LTR | 44,395,147 | 8.96 | 44,913,501 | 9.07 | 176,654,861 | 35.66 | 180,995,964 | 36.54 |
| Other | 6,441 | 0.00 | 0 | 0.00 | 0 | 0.00 | 6,441 | 0.00 |
| Unknown | 0 | 0.00 | 5,386 | 0.00 | 38,177,738 | 7.71 | 38,183,124 | 7.71 |
| Total | 51,934,073 | 10.48 | 54,351,106 | 10.97 | 242,504,965 | 48.96 | 247,262,585 | 49.92 |

**Table S 8. A comparison of the gene sets of D. longan with those of the other ten plants. Three methods—RNA-seq-based, homology-based (*A. thaliana*, *C. papaya*, *G. max*, *P. trichocarpa*, and *V. vinifera*) and de novo (GlimmerHMM and Augustus)—were used for pepper gene prediction, and the GLEAN program was used to derive consensus gene predictions.**

| Gene set | | Number | Average gene length (bp) | Average CDS length (bp) | Average exon  per gene | Average exon length (bp) | Average intron length (bp) |
| --- | --- | --- | --- | --- | --- | --- | --- |
| De novo | AUGUSTUS | 46,802 | 2,188.41 | 1,011.94 | 4.46 | 226.87 | 339.98 |
|  | GENSCAN | 48,667 | 5,944.81 | 1,031.26 | 5.19 | 198.69 | 1,172.58 |
| Homolog | *A. thaliana* | 33,110 | 2,795.57 | 970.40 | 3.85 | 252.24 | 641.05 |
|  | *C. papaya* | 45,354 | 1,943.50 | 759.90 | 3.01 | 252.82 | 590.13 |
|  | *G. max* | 41,605 | 2,645.34 | 858.67 | 3.44 | 249.87 | 733.28 |
|  | *P. trichocarpa* | 43,292 | 2,267.64 | 822.87 | 3.34 | 246.27 | 617.06 |
|  | *V. vinifera* | 39,755 | 3,080.73 | 906.17 | 3.67 | 246.92 | 814.50 |
| GLEAN | | 33,044 | 2,991.17 | 1,189.07 | 4.63 | 256.74 | 496.25 |
| RNA-Seq | | 34,174 | 3,112.37 | 1,170.85 | 4.52 | 258.78 | 495.70 |
| Final set | | 31,007 | 3,266.02 | 1,232.18 | 4.68 | 261.87 | 496.65 |

**Table S 9. The statistical analysis of functional annotation by InterPro, GO, KEGG, Swissprot, TrEMBL.**

|  | | Number | Percent (%) |
| --- | --- | --- | --- |
| Total | | 31,007 | 100 |
|  | InterPro | 23,398 | 75.64 |
|  | GO | 17,128 | 55.24 |
|  | KEGG | 17,539 | 56.56 |
|  | Swissprot | 22,986 | 74.13 |
|  | TrEMBL | 27,862 | 89.86 |
| Unannotated | | 2,984 | 9.62 |

**Table S 10. Statistics of non-coding RNA**

| Type | | Copy | Average length (bp) | Total length (bp) | % of genome |
| --- | --- | --- | --- | --- | --- |
| miRNA | | 359 | 122.51 | 43,980 | 0.0089 |
| tRNA | | 506 | 75.01 | 37,954 | 0.0077 |
|  | rRNA | 212 | 179.60 | 38,075 | 0.0077 |
| rRNA | 18S | 70 | 322.37 | 22,566 | 0.0046 |
|  | 28S | 48 | 124.10 | 5,957 | 0.0012 |
|  | 5.8S | 17 | 151.41 | 2,574 | 0.0005 |
|  | 5S | 77 | 90.62 | 6,978 | 0.0014 |
|  | snRNA | 399 | 114.76 | 45,788 | 0.0092 |
| snRNA | CD-box | 260 | 100.36 | 26,094 | 0.0053 |
|  | HACA-box | 25 | 121.72 | 3,043 | 0.0006 |
|  | splicing | 114 | 146.06 | 16,651 | 0.0034 |

**Table S 11. Statistical analysis of clustered gene families by OrthoMCL among eleven species.**

| Species | Genes number | Genes in families | Unclustered genes | Family number | Unique families | Average genes per family |
| --- | --- | --- | --- | --- | --- | --- |
| *D. longan* | 31007 | 26261 | 4746 | 14961 | 763 | 1.76 |
| *T. cacao* | 28,624 | 23,747 | 4,877 | 15,364 | 603 | 1.55 |
| *A. thaliana* | 26,637 | 22,905 | 3,732 | 13,406 | 697 | 1.71 |
| *C. sinensis* | 28,494 | 24,328 | 4,166 | 15,000 | 729 | 1.62 |
| *C. papaya* | 25,599 | 18,615 | 6,984 | 13,763 | 498 | 1.35 |
| *P. trichocarpa* | 40,996 | 33,008 | 7,988 | 15,630 | 856 | 2.11 |
| *G. max* | 53,451 | 44,111 | 9,340 | 15,908 | 1,760 | 2.77 |
| *V. vinifera* | 25,329 | 19,076 | 6,253 | 13,570 | 592 | 1.41 |
| *M. acuminata* | 36,538 | 25,380 | 11,158 | 12,519 | 882 | 2.03 |
| *P. persica* | 27,792 | 24,072 | 3,720 | 15,326 | 408 | 1.57 |
| *A. chinensis* | 39,040 | 25,937 | 13,103 | 13,702 | 1,296 | 1.89 |
| *M. domestica* | 61,721 | 44,312 | 17,409 | 17,740 | 3,641 | 2.50 |

**Table S 12. Syntenic analysis between longan and popular**

| Species | # of Synteny Blocks | Average Collinear Genes Per Block | # of Collinear Genes in All Blocks | Mean Block Length in Current Species |
| --- | --- | --- | --- | --- |
| *D . longan* | 2,106 | 8.50 | 17,901 | 766,029 |
| *P . trichocarpa* | 883 | 19.76 | 17,447 | 709,555 |

**Table S 13. Origin of longan** cultivars

| Species | Accession | [Abbreviation](javascript:void(0);) | Characteristics | Original locality |
| --- | --- | --- | --- | --- |
| *D. longan* | Honghezi | HHZ | middle-maturing | Seedling plant, Fuzhou, Fujian, China |
| *D. longan* | Dongbi | DB | early-maturing | Kaiyuan Temple, Quanzhou, Fujian, China |
| *D. longan* | Jiuyuewu | JYW | late-maturing | Putian, Fujian, China |
| *D. longan* | Lidongben | LDB | special late-maturing | Putian, Fujian, China |
| *D. longan* | Wulongling | WLL | yield | Putian, Fujian, China |
| *D. longan* | Shuinanyihao | SN1H | large fruit type, disease- resistant variety | Putian, Fujian, China |
| *D. longan* | Youtanben | YTB | late-maturing | Putian, Fujian, China |
| *D. longan* | Shieryue | SEY | special late-maturing | Zhangpu, Fujian, China |
| *D. longan* | Jiaohe/Baihe | JHLY | aborted-seeded | Quanzhou, Fujian, China |
| *D. longan* | Fuyan | FY | disease-resistant variety | Quanzhou, Fujian, China |
| *D. longan* | Shixia | SX | early-maturing, good quality | Guangdong, China |
| *D. longan* | Miaoqiao | MQ | late-maturing, yield, | Thailand |
| *D. longan* | Sijimi | SJM | multiple flowering | South-East Asia |

**Table S 14. The statistics of resequencing data**

| sample | raw | clean | Q20 | GC | map | map  rate | coverage | depth |
| --- | --- | --- | --- | --- | --- | --- | --- | --- |
| DB | 4,356,236,700 | 3,943,533,600 | 95.81 | 35.85 | 3,058,619,130 | 0.78 | 86.00 | 7.16 |
| FY | 5,438,650,860 | 4,331,673,360 | 94.08 | 35.53 | 3,245,914,440 | 0.75 | 84.50 | 7.73 |
| HHZ | 3,633,336,900 | 3,515,168,340 | 97.12 | 36.17 | 2,956,689,900 | 0.84 | 83.74 | 7.10 |
| JHLY | 4,005,440,280 | 3,636,724,860 | 95.83 | 35.71 | 2,707,820,010 | 0.74 | 81.31 | 6.70 |
| JYW | 3,619,499,040 | 3,495,851,460 | 97.04 | 36.50 | 2,692,905,570 | 0.77 | 81.97 | 6.61 |
| LDB | 3,440,295,720 | 3,319,813,440 | 96.98 | 36.26 | 2,568,100,050 | 0.77 | 83.12 | 6.22 |
| MQ | 3,266,595,900 | 3,155,470,200 | 96.95 | 35.99 | 2,202,841,080 | 0.70 | 78.02 | 5.68 |
| SEY | 3,460,857,300 | 3,356,221,860 | 97.21 | 36.17 | 2,659,520,970 | 0.79 | 81.06 | 6.60 |
| SJM | 3,719,967,840 | 3,579,070,320 | 96.62 | 35.84 | 2,646,843,210 | 0.74 | 80.47 | 6.62 |
| SL1H | 3,384,083,160 | 3,265,388,820 | 96.97 | 36.07 | 2,538,109,170 | 0.78 | 82.63 | 6.18 |
| SX | 3,215,592,900 | 3,110,125,860 | 97.14 | 36.09 | 2,342,484,810 | 0.75 | 79.44 | 5.93 |
| WIL | 3,662,677,620 | 3,539,435,220 | 97.16 | 35.62 | 2,830,379,490 | 0.80 | 82.07 | 6.94 |
| YTB | 3,619,054,800 | 3,504,438,180 | 97.10 | 36.18 | 2,829,583,080 | 0.81 | 83.83 | 6.79 |

**Table S 15.** **The statistics of SNP distribution in longan cultivars**

| Sample Name | SNP | Homo | Hete | Syn_CDS | Nonsyn_CDS | mRNA | SNP density /1kb |
| --- | --- | --- | --- | --- | --- | --- | --- |
| DB | 344,715 | 56,541 | 288,174 | 10,307 | 25,133 | 92,379 | 0.008064193 |
| FY | 510,078 | 163,941 | 346,137 | 13,960 | 33,202 | 123,515 | 0.01214383 |
| HHZ | 243,239 | 93,821 | 149,418 | 5,104 | 12,030 | 44,400 | 0.005843665 |
| JHLY | 433,827 | 165,193 | 268,634 | 11,899 | 28,569 | 105,239 | 0.01073418 |
| JYW | 307,506 | 121,516 | 185,990 | 7,537 | 17,479 | 65,066 | 0.007547135 |
| LDB | 220,862 | 65,910 | 154,952 | 5,781 | 14,183 | 52,334 | 0.005345856 |
| MQ | 402,890 | 146,988 | 255,902 | 10,813 | 25,511 | 188,389 | 0.010388965 |
| SEY | 324,263 | 129,866 | 194,397 | 9,606 | 22,994 | 105,684 | 0.008048242 |
| SJM | 424,688 | 162,884 | 261,804 | 11,982 | 28,280 | 105,139 | 0.010617312 |
| SN1H | 285,913 | 102,151 | 183,762 | 8,249 | 19,200 | 72,069 | 0.006960833 |
| SX | 323,939 | 121,016 | 202,923 | 8,748 | 21,205 | 154,633 | 0.008203563 |
| WLL | 334,500 | 137,997 | 196,503 | 8,376 | 20,140 | 151,166 | 0.008199957 |
| YTB | 320,139 | 92,823 | 227,316 | 9,189 | 21,350 | 79,370 | 0.007682561 |
| [Average](javascript:void(0);) | 357,737 | 131,910 | 225,828 | 9,882 | 23,315 | 106,931 | ---- |

Notes: Homo: homozygous; Hete: heterozygous; CDS: Coding Sequence; Non_syn: non-synonymous mutations; Syn: synonymous mutations

**Table S 16. The statistics of Indel distribution** in longan cultivars

| Sample Name | Indel | Insertion | Deletion | Indel CDS | indel UTR_3 | indel UTR_5 | indel mRNA | InDel Density /1kb |
| --- | --- | --- | --- | --- | --- | --- | --- | --- |
| DB | 19,823 | 9,467 | 10,356 | 1,380 | 79 | 98 | 2,261 | 0.000463735 |
| FY | 28,482 | 13,369 | 15,113 | 2,240 | 144 | 166 | 3,604 | 0.000678093 |
| HHZ | 16,496 | 7,881 | 8,615 | 1,125 | 80 | 78 | 1,888 | 0.000396306 |
| JHLY | 28,797 | 13,523 | 15,274 | 2,204 | 152 | 169 | 3,612 | 0.000712524 |
| JYW | 20,985 | 9,854 | 11,131 | 1,285 | 92 | 100 | 2,141 | 0.000515036 |
| LDB | 16,601 | 7,731 | 8,870 | 1,171 | 90 | 100 | 1,998 | 0.000401819 |
| MQ | 29,791 | 14,283 | 15,508 | 2,185 | 156 | 159 | 3,389 | 0.000768194 |
| SEY | 22,656 | 10,780 | 11,876 | 1,880 | 130 | 127 | 3,100 | 0.000562324 |
| SJM | 29,937 | 14,125 | 15,812 | 2,145 | 141 | 144 | 3,497 | 0.000748433 |
| SN1H | 20,800 | 9,742 | 11,058 | 1,336 | 108 | 96 | 2,204 | 0.000506396 |
| SX | 23,520 | 11,113 | 12,407 | 1,815 | 144 | 139 | 2,954 | 0.00059563 |
| WLL | 24,843 | 11,723 | 13,120 | 1,670 | 101 | 136 | 2,650 | 0.000609003 |
| YTB | 19,196 | 9,032 | 10,164 | 1,512 | 116 | 108 | 2,464 | 0.000460658 |
| [Average](javascript:void(0);) | 23,225 | 10,971 | 12,254 | 1,688 | 118 | 125 | 2,751 | ---- |

**Table S 17. The statistics of transcriptome data from 9 longan samples.**

| Sample  Name | Clean reads | Genome map  Rate (%) | Gene map  Rate (%) | Expressed  Gene | Expressed  Transcripts | Expressed  Exon | Novel  Transcripts | Extend  Gene | Alternative  Splicing | SNP | Indel |
| --- | --- | --- | --- | --- | --- | --- | --- | --- | --- | --- | --- |
| Root | 45,777,094 | 76.36 | 52.19 | 22,205 | 22,205 | 108,585 | 1,757 | 9,632 | 33,329 | 131,312 | 3,800 |
| Stem | 48,279,464 | 72.29 | 51.99 | 22,103 | 22,103 | 107,388 | 1,621 | 9,618 | 31,141 | 137,321 | 3,715 |
| Leaf | 60,115,302 | 79.40 | 57.62 | 21,267 | 21,267 | 104,781 | 1,778 | 9,025 | 37,216 | 156,253 | 4,673 |
| Flower bud | 50,038,814 | 76.40 | 54.24 | 23,118 | 23,118 | 111,476 | 1,876 | 9,995 | 34,198 | 142,812 | 3,867 |
| Flower | 59,981,886 | 66.49 | 48.37 | 22,407 | 22,407 | 108,693 | 1,696 | 9,487 | 31,174 | 132,539 | 3,577 |
| Young_fruit | 47,000,538 | 77.56 | 55.46 | 22,737 | 22,737 | 109,848 | 1,999 | 9,528 | 35,998 | 161,897 | 4,322 |
| Pericarp | 59,767,958 | 62.91 | 43.09 | 21,777 | 21,777 | 106,329 | 1,774 | 9,354 | 35,384 | 151,272 | 4,028 |
| Pulp | 59,607,282 | 53.55 | 36.75 | 19,322 | 19,322 | 96,105 | 1,673 | 8,126 | 28,058 | 105,007 | 2,587 |
| Seed | 59,934,484 | 69.65 | 52.72 | 22,320 | 22,320 | 108,230 | 1,953 | 9,324 | 32,416 | 137,403 | 3,821 |

**Table S18. The result of assessing Unigene completeness using BUSCO**

| #BUSCO was run in mode: trans | |  |
| --- | --- | --- |
| Summarized benchmarks in BUSCO notation: | | |
| C:87%[D:36%],F:5.5%,M:7.1%,n:956 | | |
| Representing: |  |  |
| 483 Complete Single-copy BUSCOs | | |
| 352 Complete Duplicated BUSCOs | | |
| 53 Fragmented BUSCOs | |  |
| 68 Missing BUSCOs | |  |
| 956 Total BUSCO groups searched | | |

**Table S 19. Numbers of longan genes that encode domains similar to plant R proteins and comparison with other sequenced** genomes

| Predicted Protein Domains | Letter Code | *D. longan* | *C. sinensis* | *T. cacao* | *V. vinifera* | *A. chinensis* | *P.* *persica* | *C. papaya* | *M. domestica* |
| --- | --- | --- | --- | --- | --- | --- | --- | --- | --- |
| NBS_LRR | NL | 258 | 166 | 114 | 177 | 55 | 177 | 20 | 415 |
| NBS | N | 122 | 95 | 47 | 92 | 35 | 57 | 26 | 225 |
| TIR_NBS_LRR | TNL | 23 | 77 | 9 | 18 | 1 | 128 | 6 | 195 |
| TIR_NBS | TN | 4 | 17 | 4 | 3 | 0 | 15 | 1 | 87 |
| CC_NBS_LRR | CNL | 150 | 111 | 91 | 39 | 13 | 42 | 5 | 79 |
| CC_NBS | CN | 37 | 43 | 27 | 12 | 6 | 6 | 2 | 34 |
| LRR_RLK | LR | 338 | 325 | 254 | 234 | 259 | 268 | 134 | 477 |
| total | --- | 932 | 834 | 546 | 575 | 369 | 693 | 194 | 1512 |
