# Supplementary material for: Genome-wide sequencing of longan (Dimocarpus longan Lour.) provides insights into molecular basis of its polyphenol-rich characteristics
Source: Gigascience. 2017 Mar 28;6(5):1–14. doi: 10.1093/gigascience/gix023 (PMC5467034; doi:10.1093/gigascience/gix023)
Supplement: Additional file 2: — Figures S1 to S7 [file gix023_Additional_file_2-12.9.doc]

**Figure S1.** **17-kmer estimation of genome sizes.** The x-axis is k-mer depth, the left y-axis is the k-mer frequency of longan. The figure illustrates that two peaks depths of longan are 32.4 and 66, respectively, suggesting a high proportion of repetitive elements in these species.

**Figure S2. A comparative analysis of GC content among longan, papaya and grape.**

**Figure S3 GC depth analysis of longan genome.** The x-axis is GC content, the left y-axis is the sequencing depth of longan.

**Figure S4 Gene Ontology classification of Gene family expansions and contractions.** A. Gene Ontology classification of Gene family expansions. B. Gene Ontology classification of Gene family contractions. Gene ontology (GO) term assignments based on significant plant species hits against the NR database were summarized into three main GO categories (biological process, cellular component,molecular function).


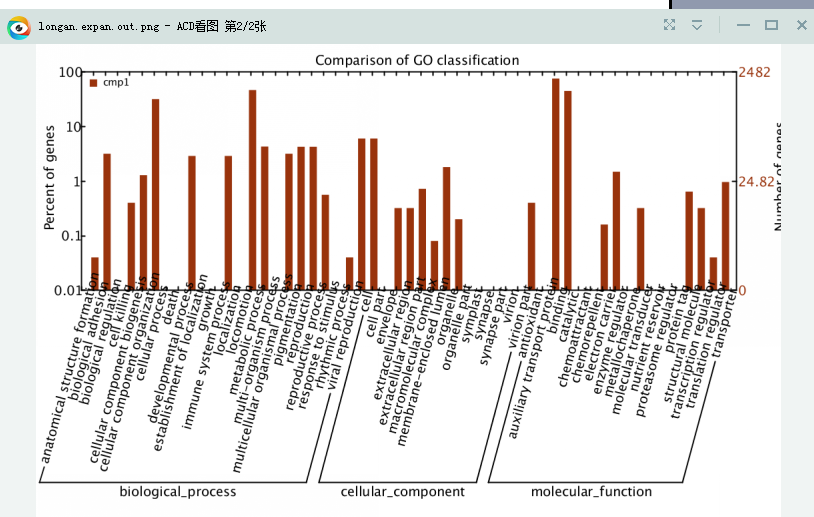

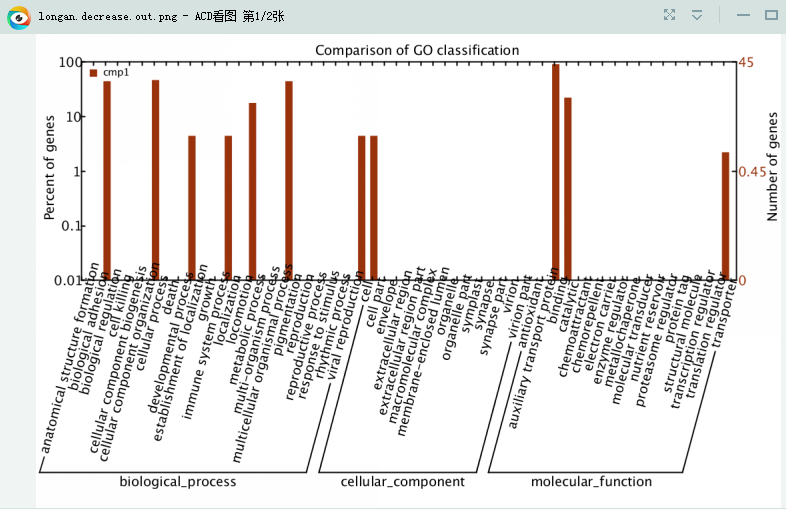

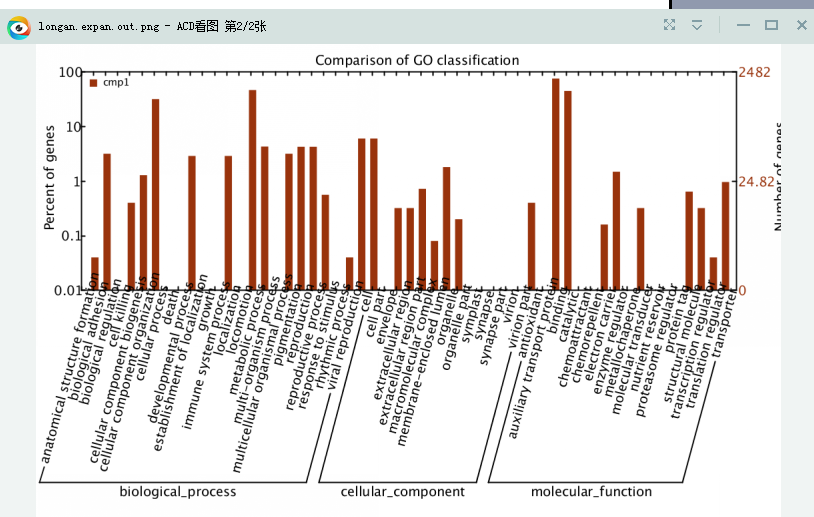

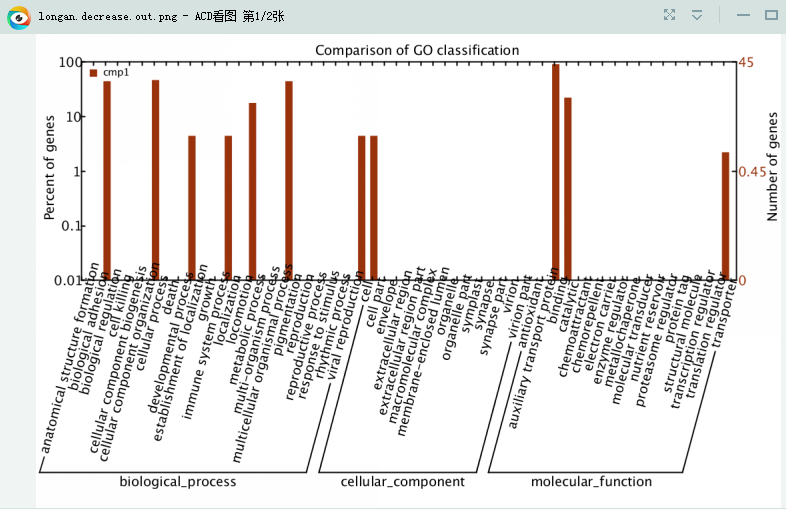

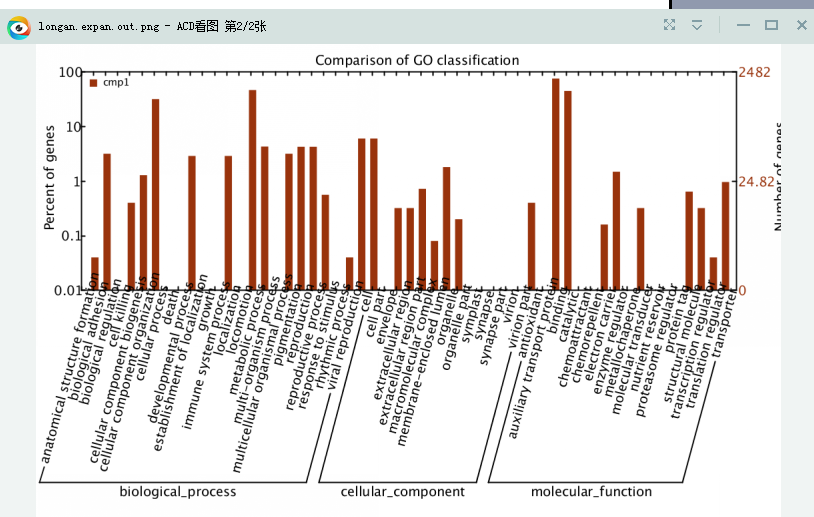

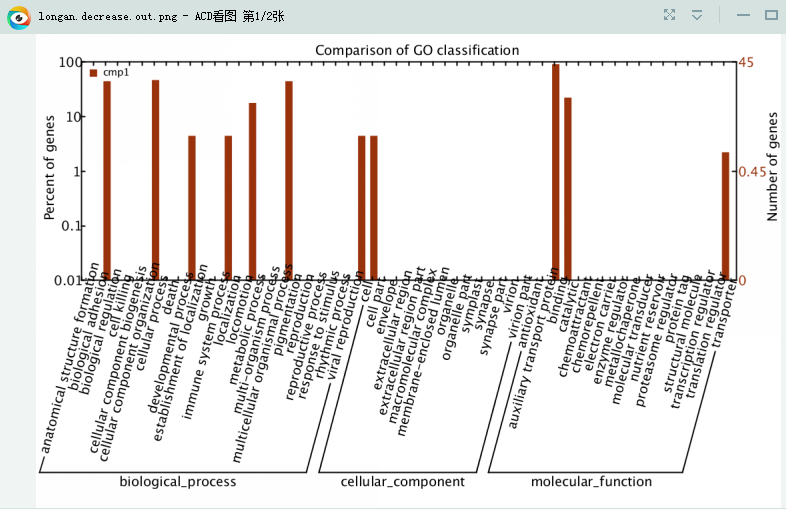


A.

B.

A.


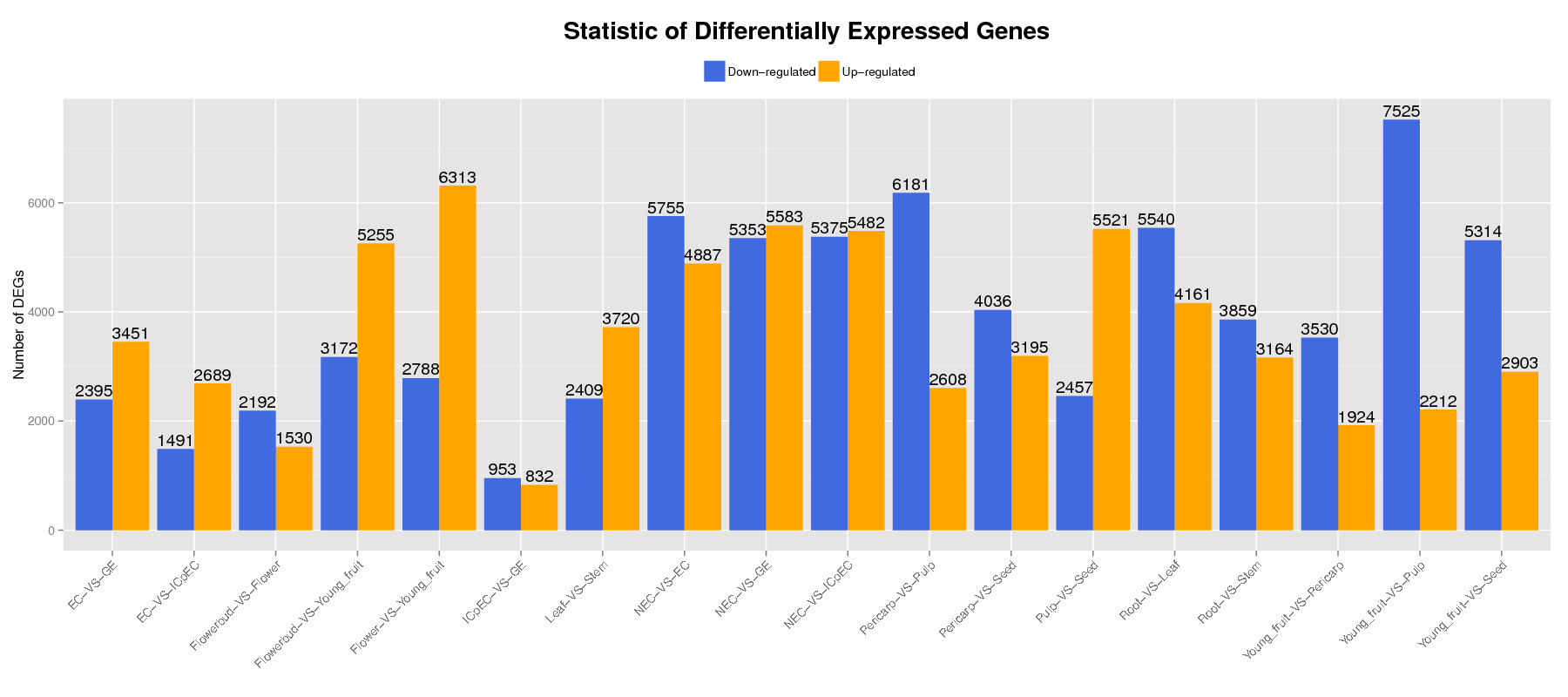
**Figure S5 Statistic of differentially expressed genes of** 12 pair-wise comparisons, including Root *VS* Stem, Root *VS* Leaf, Leaf *VS* Stem, Flower_bud *VS* Flower, Flower_bud *VS* Young_fruit, Flower *VS* Young_fruit, Young_fruit *VS* Pulp, Young_fruit *VS* Seed, Pericarp *VS* Pulp, Pericarp *VS* Seed, and Pulp *VS* Seed**.**

**Figure S6 GO functional classification for** 12 pair-wise comparisons, including Root *VS* Stem, Root *VS* Leaf, Leaf *VS* Stem, Flower_bud *VS* Flower, Flower_bud *VS* Young_fruit, Flower *VS* Young_fruit, Young_fruit *VS* Pulp, Young_fruit *VS* Seed, Pericarp *VS* Pulp, Pericarp *VS* Seed, and Pulp *VS* Seed**.**

**
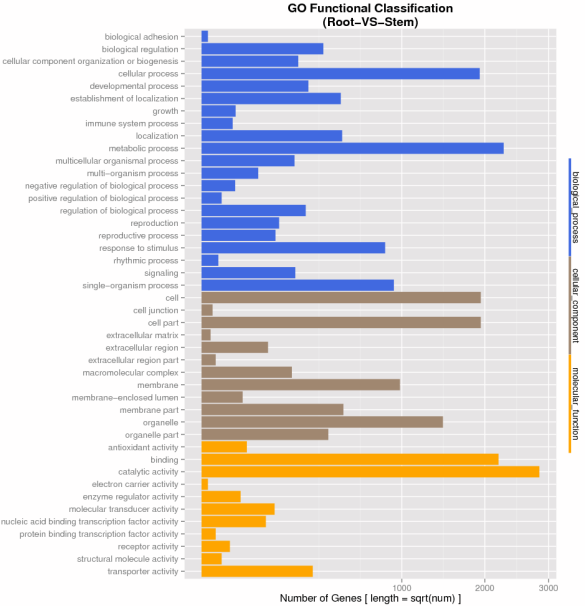

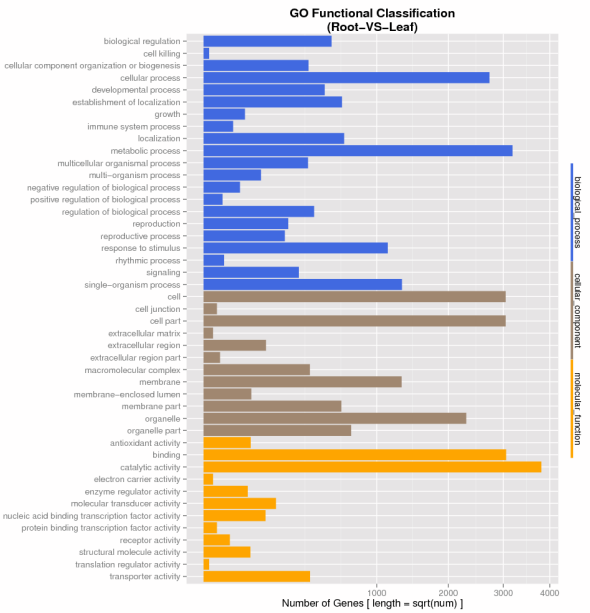

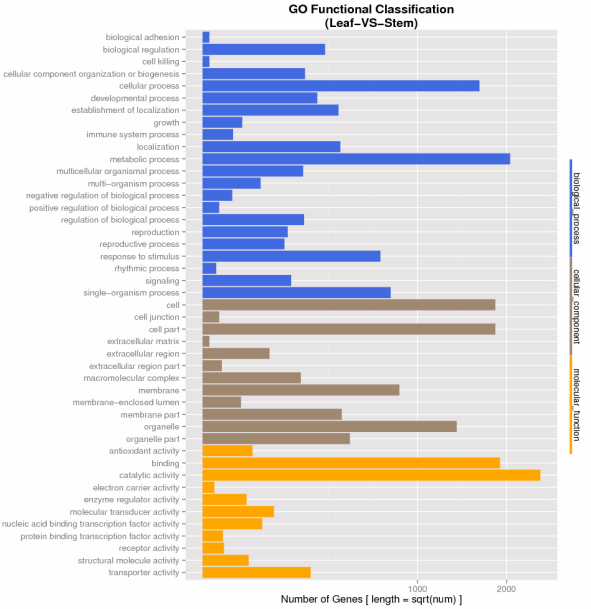

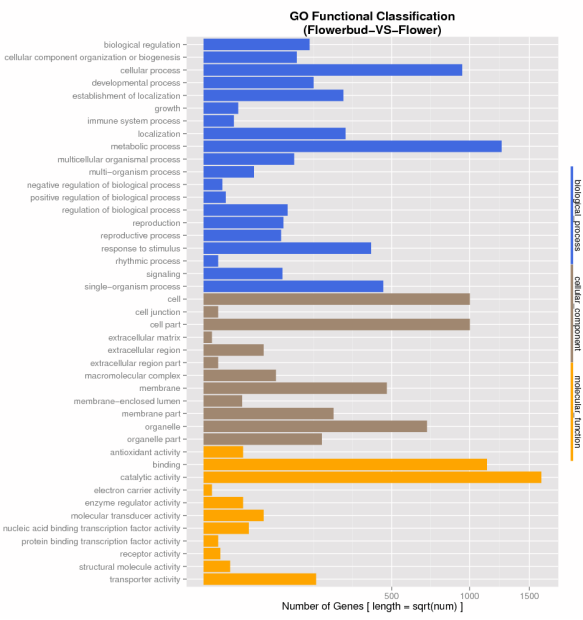

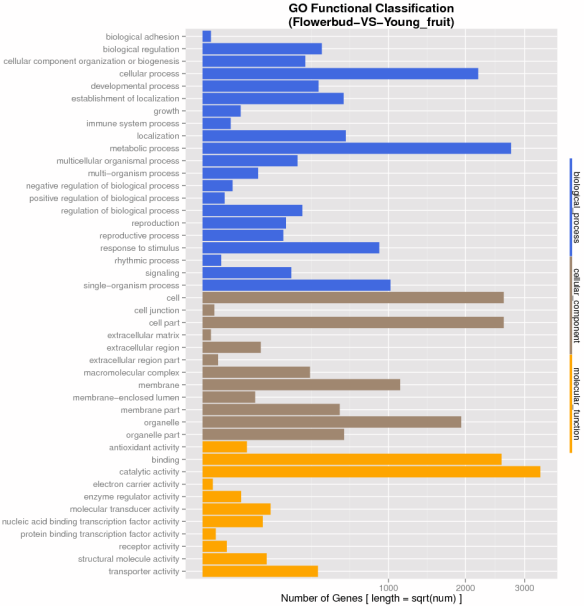

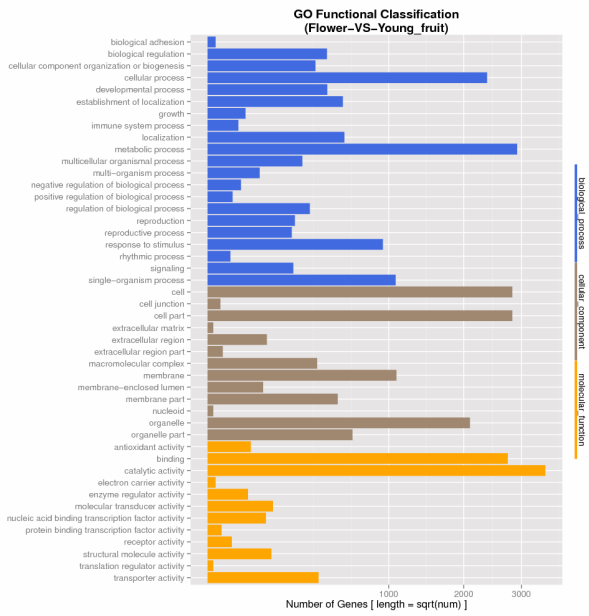

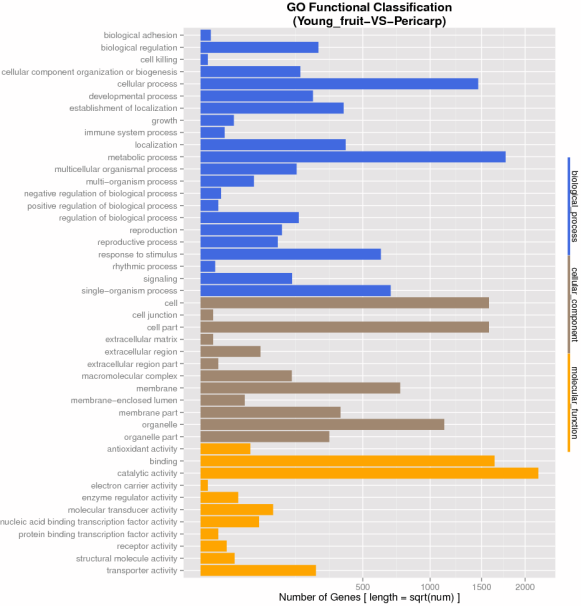

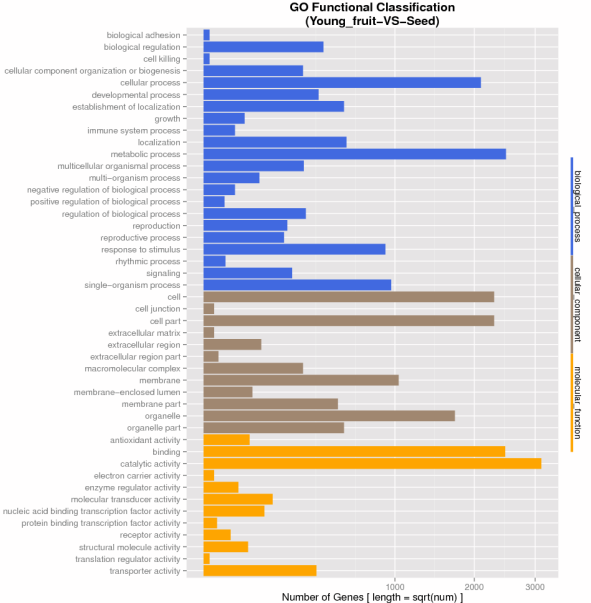

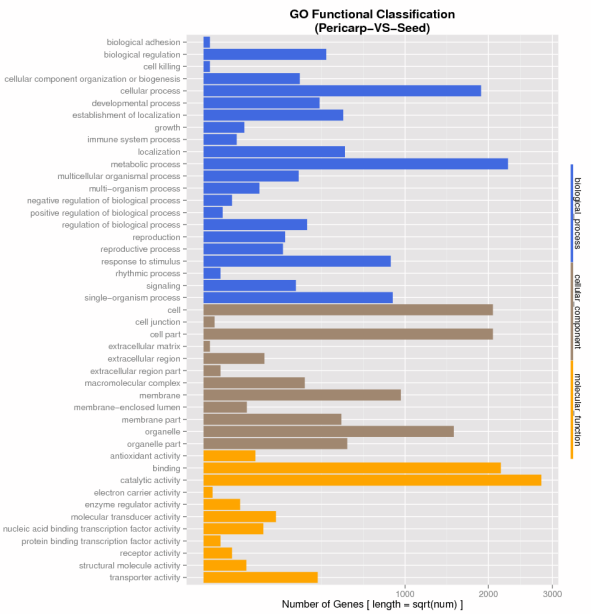

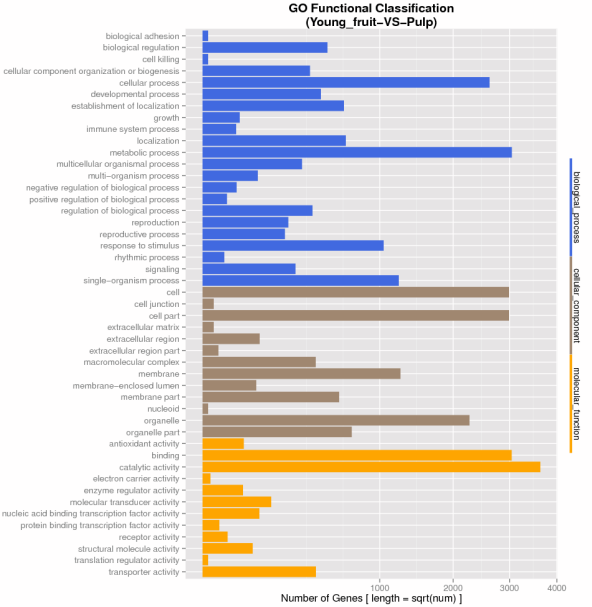

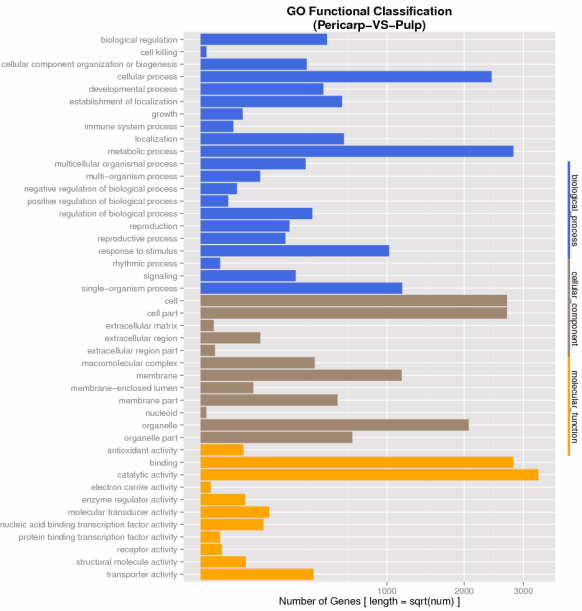

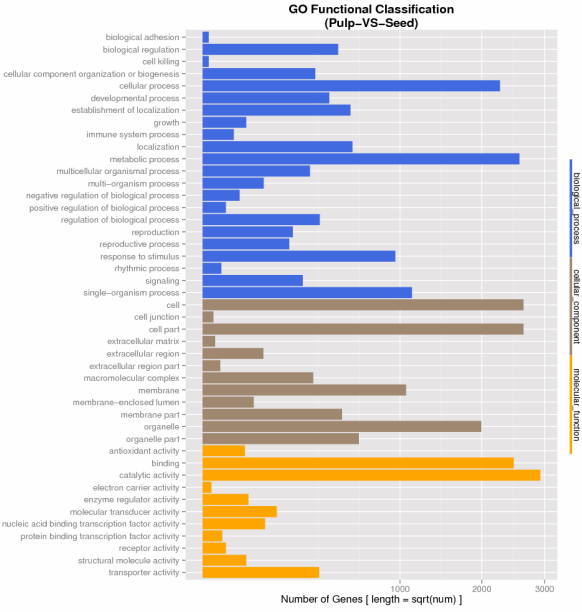
**

**Figure S7 Statistic of pathway enrichment for 9 samples**

**
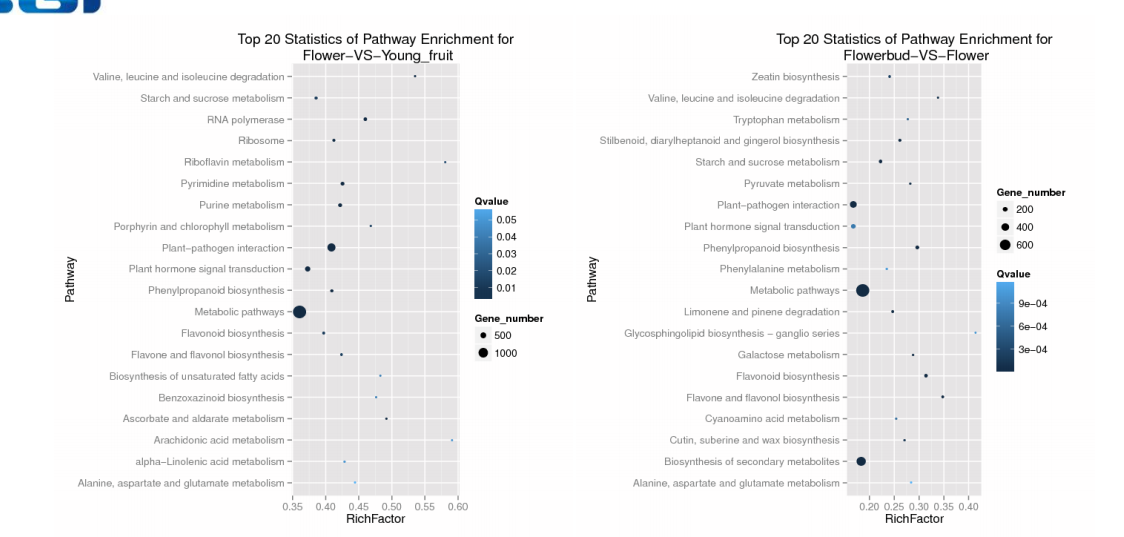

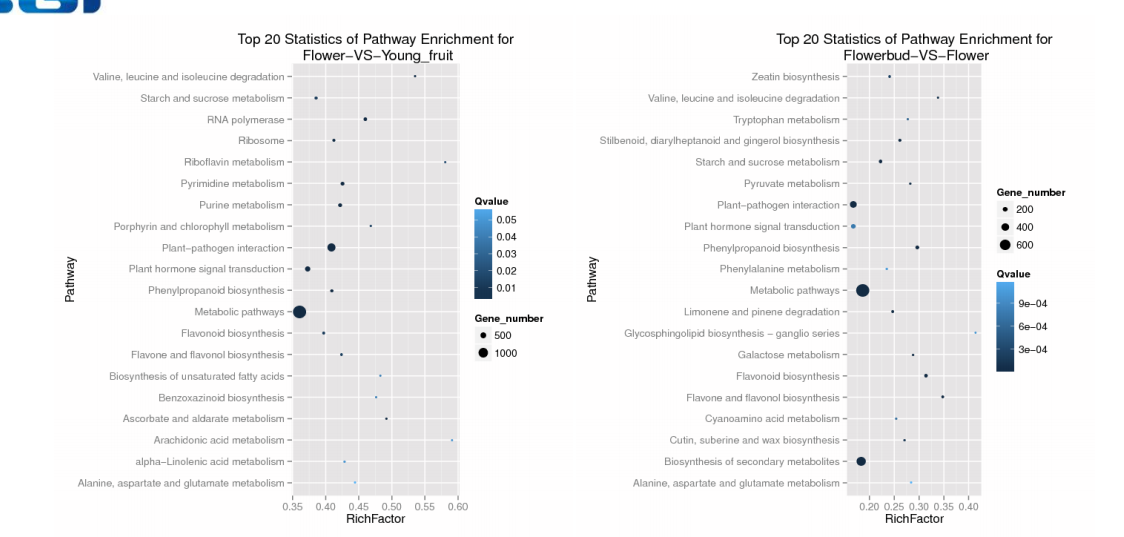
**

**
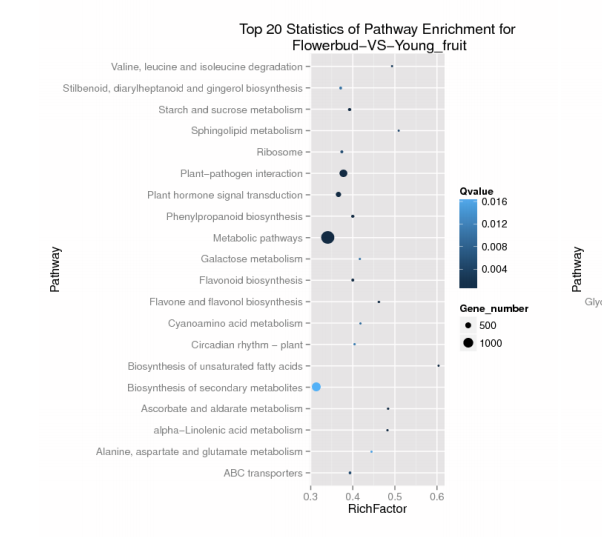

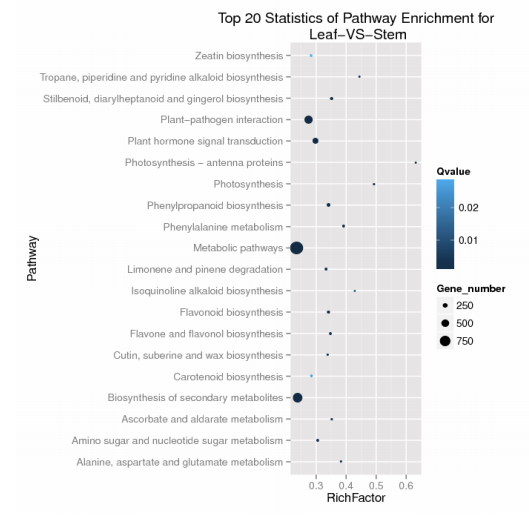

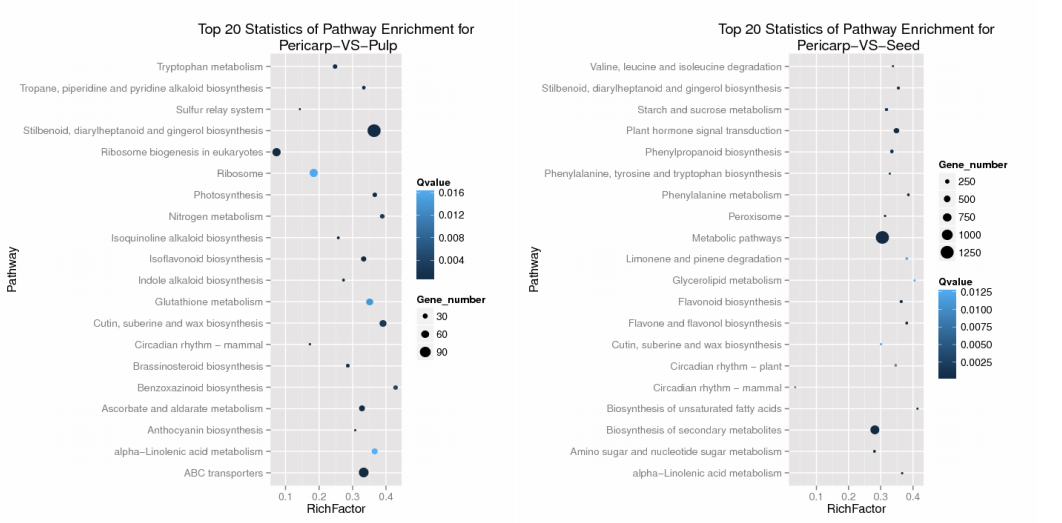
**

**
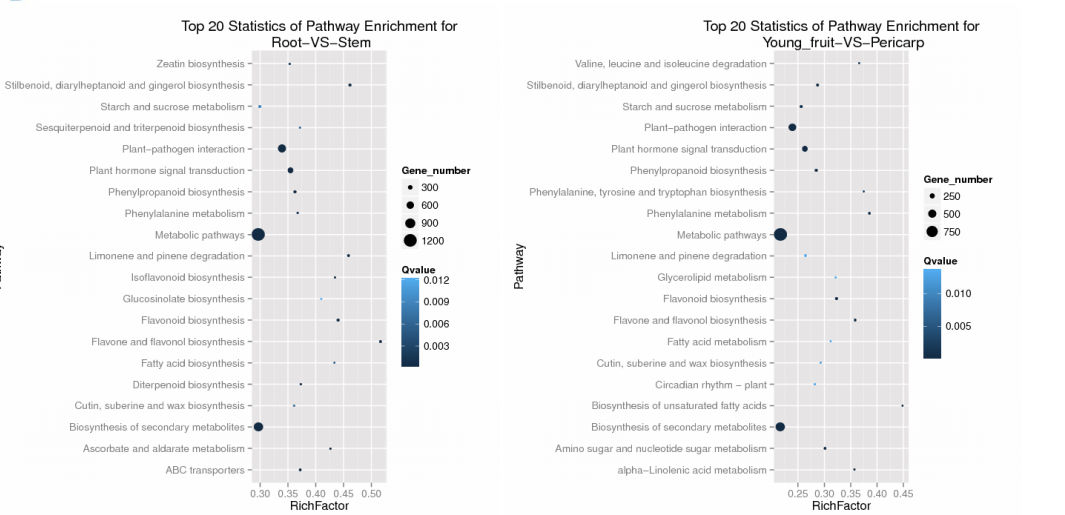
**

**
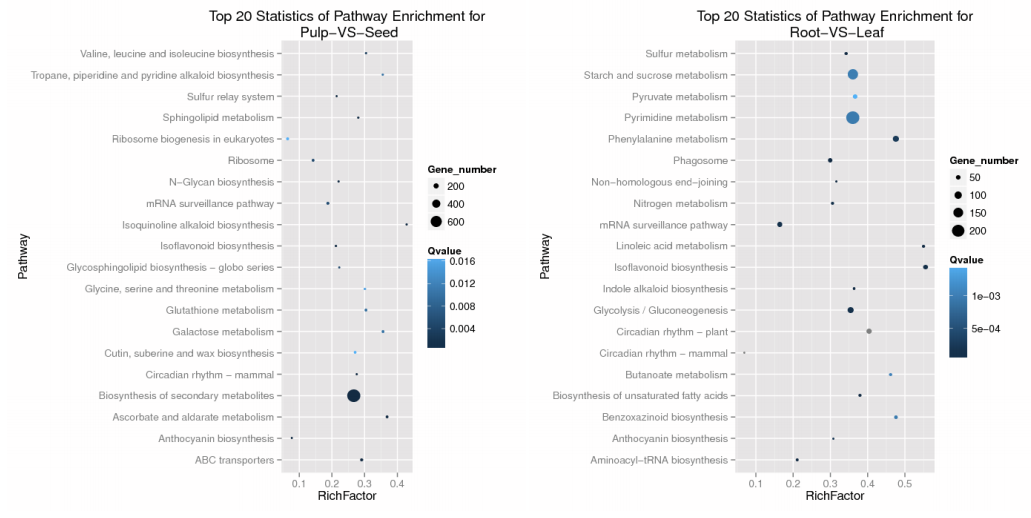
**

**
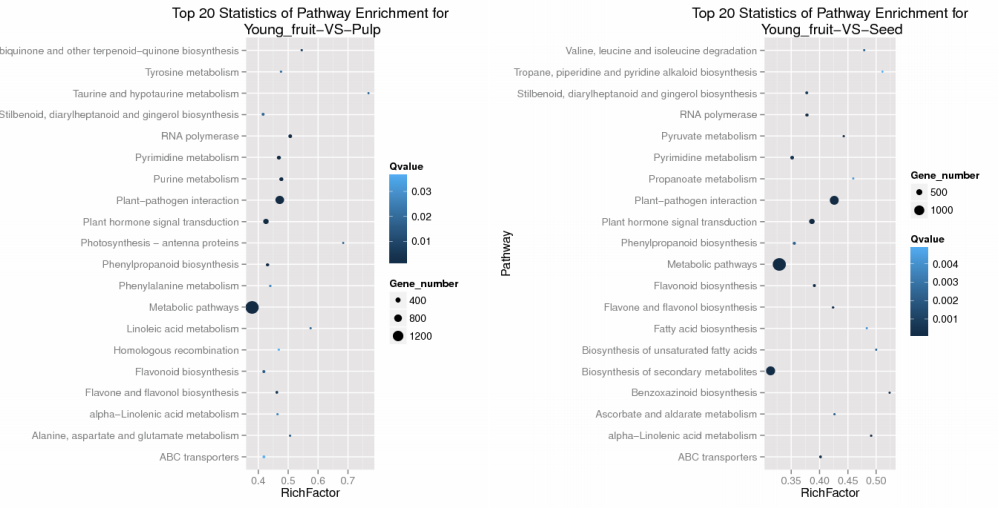
**
