# Supplementary material for: Genome-wide sequencing of longan (Dimocarpus longan Lour.) provides insights into molecular basis of its polyphenol-rich characteristics
Source: Gigascience. 2017 Mar 28;6(5):1–14. doi: 10.1093/gigascience/gix023 (PMC5467034; doi:10.1093/gigascience/gix023)
Supplement: Response_to_reviewers_comments_Original_Submission.pdf [file gix023_Response_to_reviewers_comments_Original_Submission.pdf]

Author's point-by-point responses to editors and the reviewers's comments

Editors:

Response: The revised manuscript has been re-worded and edited by a native English-speaking editor. And we added more details about SNPs, indels, differentially expressed genes, and alternative- splicing events of 9 RNA-seq libraries of various tissues in the revised version, please see the detials in page 17 to 21.

Reviewer #1:
1. Written English should be substantially improved. Seems to be written by several authors. Background and biology sections are weak. Bioinformatics sections seem to be much easier to follow. Not in itself enough to preclude publication but will need careful editing for grammar, spelling mistakes, readability etc.

Response: The revised manuscript has been edited by a native English-speaking editor.

2. Page 3 line 52: what does "lack of knowledge of genetic background, resulting in a major bottleneck in longan breeding" mean? Do you mean the pedigree of the material is unknown therefore you can't manage inbreeding?

Response: The sentence of "lack of knowledge of genetic background, resulting in a major bottleneck in longan breeding" was revised as follow:

Page 4 Line12 to 20: "Cultivar identification and characterization are the first steps for fruit introduction and breeding improvement [7]. In China, there are more than 300 longan varieties, most are landraces and farm varieties, although a few wild populations exist in Hainan, Guangdong, Guangxi, and Yunnan provinces [7, 8]. However, only 30–40 varieties are grown commercially worldwide. Longan breeding improvement via conventional breeding strategies has been hindered by its long juvenility, genetic heterozygosity, and plant size [1]. To identify cultivars and improve longan breeding, knowledge of the longan genetic background is required. "

3. Page 5 line 9 to 17: here and elsewhere, these comparisons of genome size between longan and other species are not very meaningful without context. What are you trying to show by these comparisons?

Response: We re-worded the related sentences throughout in the revised manuscript. Please see the details in the manuscript.

4. There is a description of the character of the repetitive element, some comparisons with other plants but no interpretation of the biological meaning.

Response: we re-worded the related sentences throughout in the revised manuscript. Please see the details in the page 8,line 18-21 to page 9, in the revised one.

5. Page 7 line 15: here and elsewhere, Table S11 should be Table S12.

Response: we re-worded the table numbers throughout in the revised manuscript.

6. Page 7 line 39: CAFÉ analysis of gene family expansion and contraction. This is an interesting analysis. The authors reported 386 expanded families while only 12 have contracted. However, what was the baseline against which the gene families were expanded or contracted? An average plant?

Response: actually, we found a total of 2,849 expanded gene families and 2,842 contracted families; however, only 386 expanded families (7,839 genes) and 12 contracted families (53 genes), accounting for 19.96% and 0.13% of the total coding-genes (39,282), respectively, were found to be statistical significant

at P <0.05 (Supplemental EXCEL Files 2 and 3). Please see the detials in Line 7 to 21 in Page12 in the revised version.

7. Page 11 line 23 to 26: The authors reported " RNA-Seq data confirmed a majority of annotated introns, identified thousands of novel alternatively spliced mRNA genes ...", however, I could not find a description of how or what method they used to identify alternatively spliced genes/isoforms? Also it would be interesting to know the proportion of various forms of alternative splicing events (i.e. exon skipping, intron retention etc.)

Response: as your and editor's suggestion, we added more details about SNPs, indels, differentially expressed genes, and alternative- splicing events of 9 RNA-seq libraries of various tissues in the revised version, please see the detials in page 17 to 21.

8. Page 12 line 12: There does seem to be elevated SDH gene family numbers relative to some other woody plants. But it would be interesting to see comparisons with poplar and eucalyptus genomes as well, not just the horticultural species.

Response: as your suggestion, the genes related biosynthesis of polyphenols were compared with poplar, eucalyptus genomes and horticultural species. Please see the details in the page 22 to 26 in the revised version.

9.Page 15 line 23 to 56 - What is the biological significance of different numbers of disease resistance genes in different subclasses? Why might longan show this distribution?

Response: we re-worded, please see the details in page page 27 to page 29.

10. Page 17 line 48 to 55: I assume the scaffolds were repeatmasked prior to annotating genes. However, the authors need to mention it in the text.

Response: yes, the scaffolds were repeatmasked prior to annotating genes. According to your suggestion, we mentioned it in the page 31, line 19.

11. I strongly recommend assessing genome assembly and annotation completeness with BUSCO (http://busco.ezlab.org/) and compare the result with other horticultural species mentioned in the manuscript.

Response: as your suggestion, we assessed genome assembly and annotation completeness with BUSCO (Page 8, line5 to 15). BUSCOs results showed that the quality of our assembly is high.

Reviewer #2:
1 .If resubmitted as a research article or a research article submitted along with a data note submission, it should be noted that the manuscript has numerous typos and a few sections that need to be rewritten for clarity. The authors should take the opportunity to fix these before submitting the manuscript.

Response: actually, we plan to re-submit as a resarch article, and we re-worded throughout the revised manuscript, and added more information about SNPs, indels, differentially expressed genes, and alternative-splicing events of 9 RNA-seq libraries of various tissues. Please see the details in the new version.
