# Supplementary material for: Genome-wide sequencing of longan (Dimocarpus longan Lour.) provides insights into molecular basis of its polyphenol-rich characteristics
Source: Gigascience. 2017 Mar 28;6(5):1–14. doi: 10.1093/gigascience/gix023 (PMC5467034; doi:10.1093/gigascience/gix023)
Supplement: response_to_reviewer_comments_Revision_2.pdf [file gix023_response_to_reviewer_comments_Revision_2.pdf]

Author's point-by-point responses to the editor’s comments

1. "....seeds all have been widely used as traditional Chinese medicines for several diseases, including leucorrhea, kidney disorders, allergies, cancer, diabetes, and cardiovascular disease, because they contain bioactive compounds such as phenolic acids, flavonoids, and polysaccharides [4], which exhibit antimicrobial, antioxidant, anticancer, antityrosinase, and inflammatory properties (5,6)"

the scientific evidence (references 5,6) for these medical properties appears to be rather weak, overall. I think it is ok if you state that it is used in TCM, but please remove the statement claiming that there was evidence for antimicrobial, antioxidant, anticancer, antityrosinase, and inflammatory properties .

Response: as your suggestion, the scientific evidence (references 5,6) appears to be weak, so we removed the sententce of "which exhibit antimicrobial, antioxidant, anticancer, antityrosinase, and inflammatory properties (5,6)" in the revised manuscript.

2. When making these changes, please also change the article type to "Research" in our submission system. (Currently it is "Data Note"). Please also doublecheck that the format of your paper conforms to our Instructions for a "Research Article".

Response: we have changed the article type to 'Research' in GIGA submission system.
