# Supplementary material for: Genome-wide sequencing of longan (Dimocarpus longan Lour.) provides insights into molecular basis of its polyphenol-rich characteristics
Source: Gigascience. 2017 Mar 28;6(5):1–14. doi: 10.1093/gigascience/gix023 (PMC5467034; doi:10.1093/gigascience/gix023)
Supplement: Response_to_reviewer_comment_Revision_1.pdf [file gix023_Response_to_reviewer_comment_Revision_1.pdf]

Author's point-by-point responses to the reviewers's comments

Reviewer #1:
1. The authors need to clarify the change of gene and coding sequence size (page 9, lines 26-30), which is different from Table S8.
Response: The data of table S8 was changed in the revised manuscript.
2. The manuscript still has some typos, e.g. page 21 line 31
Response: We carefully check the typos throughout the revised version.

Reviewer #2:
Minor comments:
1. Page 6 - line 39: "without N sequences" change to "excluding gaps"
Response: DONE

2. Page 6 - line 59: "Generally, the N50 number is used to measure the contig or scaffold lengths for estimating the quality of a genome assembly." - remove this and just report the N50 sizes in the results. While the reporting of N50 contig and scaffold sizes is expected, the value of these metrics for estimating the quality of an assembly are somewhat dubious.
Response: DONE

3. Page 7 - line 59: "The BUSCO analysis indicated that 94% of the longan draft assembly was complete." - Consider removing this line from the reporting of the BUSCO analysis results. I think this is stretch too far to go from 94% complete BUSCOs present to having a 94% complete assembly.
Response: DONE, we have removed the line "The BUSCO analysis indicated that 94% of the longan draft assembly was complete" in the revised manuscript.

4. Page 23 - line 36 "EXCFL" change to Excel
Response: DONE

5. Use a consistent scientific notation format through the manuscript
Response: DONE

6. Page 32 - line 47 "Legacy BLAST" change to NCBI BLAST
Response: DONE

7. Page 32 - line 50 missing closing ")"
Response: DONE

8. Page 33 - line 42 - Sequencing, data, annotations (typo?) - provide a URL/DOI for the annotation if it is not going to be submitted to NCBI.
Response: It is typo. We have removed it. Supporting genome assemblies, annotations, supplemental data and custom scripts are hosted in the GigaScience GigaDB repository.
