# Supplementary material for: Genome-wide sequencing of longan (Dimocarpus longan Lour.) provides insights into molecular basis of its polyphenol-rich characteristics
Source: Gigascience. 2017 Mar 28;6(5):1–14. doi: 10.1093/gigascience/gix023 (PMC5467034; doi:10.1093/gigascience/gix023)
Supplement: Reviewer_1_Report_(Original_Submission).pdf [file gix023_Reviewer_1_Report_(Original_Submission).pdf]

Reviewer Report

Title: "Genome-wide sequencing of longan (Dimocarpus longan Lour.) provides insights into molecular basis of its polyphenol-rich characteristics"

Version: Original Submission **Date:** 8/11/2016

Reviewer name: Click here to enter text.

Reviewer Comments to Author:

The manuscript describes the sequencing, assembly and annotation of the subtropical fruit tree Longan (Dimocarpus longan Lour). This is an important project as limited or no genome sequencing data available for related tropical or subtropical fruit tree species of Sapindaceae family. The genome assembly and annotation was performed using standard pipelines and statistics for the results are commendable.

Specific comments:

- Written English should be substantially improved. Seems to be written by several authors. Background and biology sections are weak. Bioinformatics sections seem to be much easier to follow. Not in itself enough to preclude publication but will need careful editing for grammar, spelling mistakes, readability etc.

- Page 3 line 52: what does "lack of knowledge of genetic background, resulting in a major bottleneck in longan breeding" mean? Do you mean the pedigree of the material is unknown therefore you can't manage inbreeding?

- Page 5 line 9 to 17: here and elsewhere, these comparisons of genome size between longan and other species are not very meaningful without context. What are you trying to show by these comparisons?

- There is a description of the character of the repetitive element, some comparisons with other plants but no interpretation of the biological meaning.

- Page 7 line 15: here and elsewhere, Table S11 should be Table S12.

- Page 7 line 39: CAFÉ analysis of gene family expansion and contraction. This is an interesting analysis. The authors reported 386 expanded families while only 12 have contracted. However, what was the baseline against which the gene families were expanded or contracted? An average plant?

- Page 11 line 23 to 26: The authors reported " RNA-Seq data confirmed a majority of annotated introns, identified thousands of novel alternatively spliced mRNA genes …", however, I could not find a description of how or what method they used to identify alternatively spliced genes/isoforms? Also it

would be interesting to know the proportion of various forms of alternative splicing events (i.e. exon skipping, intron retention etc.)

- Page 12 line 12: There does seem to be elevated SDH gene family numbers relative to some other woody plants. But it would be interesting to see comparisons with poplar and eucalyptus genomes as well, not just the horticultural species.

- Page 15 line 23 to 56 - What is the biological significance of different numbers of disease resistance genes in different subclasses? Why might longan show this distribution?

- Page 17 line 48 to 55: I assume the scaffolds were repeatmasked prior to annotating genes. However, the authors need to mention it in the text.

- I strongly recommend assessing genome assembly and annotation completeness with BUSCO (http://busco.ezlab.org/) and compare the result with other horticultural species mentioned in the manuscript.

Level of Interest

Please indicate how interesting you found the manuscript: An article whose findings are important to those with closely related research interests

Quality of Written English

Please indicate the quality of language in the manuscript: Needs some language corrections before being published

Declaration of Competing Interests

Please complete a declaration of competing interests, considering the following questions:

- Have you in the past five years received reimbursements, fees, funding, or salary from an organisation that may in any way gain or lose financially from the publication of this manuscript, either now or in the future?
- Do you hold any stocks or shares in an organisation that may in any way gain or lose financially from the publication of this manuscript, either now or in the future?
- Do you hold or are you currently applying for any patents relating to the content of the manuscript?
- Have you received reimbursements, fees, funding, or salary from an organization that holds or has applied for patents relating to the content of the manuscript?
- Do you have any other financial competing interests?
- Do you have any non-financial competing interests in relation to this paper?

If you can answer no to all of the above, write 'I declare that I have no competing interests' below. If your reply is yes to any, please give details below.

I declare that I have no competing interests.

I agree to the open peer review policy of the journal. I understand that my name will be included on my report to the authors and, if the manuscript is accepted for publication, my named report including any attachments I upload will be posted on the website along with the authors' responses. I agree for my report to be made available under an Open Access Creative Commons CC-BY license (http://creativecommons.org/licenses/by/4.0/). I understand that any comments which I do not wish to be included in my named report can be included as confidential comments to the editors, which will not be published.

I agree to the open peer review policy of the journal
