# Supplementary material for: Genome-wide sequencing of longan (Dimocarpus longan Lour.) provides insights into molecular basis of its polyphenol-rich characteristics
Source: Gigascience. 2017 Mar 28;6(5):1–14. doi: 10.1093/gigascience/gix023 (PMC5467034; doi:10.1093/gigascience/gix023)
Supplement: Reviewer_2_report_(Original_Submission).pdf [file gix023_Reviewer_2_report_(Original_Submission).pdf]

Reviewer Report

Title: "Genome-wide sequencing of longan (Dimocarpus longan Lour.) provides insights into molecular basis of its polyphenol-rich characteristics"

Version: Original Submission **Date:** 11/1/2016

Reviewer name: John Hamilton

Reviewer Comments to Author:

The manuscript describes the sequencing, assembly, and annotation of the genome of the longan fruit, the genome resequencing of 13 cultivated longan accessions, and the sequencing of 9 RNA-seq libraries of various tissues.

Along with the generation of these data sets, the results of a number of analyses are presented including identification of gene family expansion/contraction, looking for evidence of recent WGD, an analysis of genetic diversity by resequencing longan cultivars, and analyses of the NBS-LRR genes and polyphenol biosynthesis pathways using RNA-Seq.

Overall, the generation of the above data sets and analyses will prove useful to the community.

However, the manuscript was submitted as a GigaScience 'Data Note' rather than a 'Research Article'. If the authors intended to submit the paper just as a Data Note, the paper needs to revised to remove the extensive analyses and provide more information about the methods and validation used for the sequencing, assembly and annotation of the longan genome. Also missing is the data information file that provides the metadata for the GigaDB submission that would accompany the published Data Note (https://gigascience.biomedcentral.com/submission-guidelines/preparing-your-manuscript/data-note).

If resubmitted as a research article or a research article submitted along with a data note submission, it should be noted that the manuscript has numerous typos and a few sections that need to be rewritten for clarity. The authors should take the opportunity to fix these before submitting the manuscript.

Level of Interest

Please indicate how interesting you found the manuscript: An article whose findings are important to those with closely related research interests

Quality of Written English

Please indicate the quality of language in the manuscript: Needs some language corrections before being published

Declaration of Competing Interests

Please complete a declaration of competing interests, considering the following questions:

- Have you in the past five years received reimbursements, fees, funding, or salary from an organisation that may in any way gain or lose financially from the publication of this manuscript, either now or in the future?
- Do you hold any stocks or shares in an organisation that may in any way gain or lose financially from the publication of this manuscript, either now or in the future?
- Do you hold or are you currently applying for any patents relating to the content of the manuscript?
- Have you received reimbursements, fees, funding, or salary from an organization that holds or has applied for patents relating to the content of the manuscript?
- Do you have any other financial competing interests?
- Do you have any non-financial competing interests in relation to this paper?

If you can answer no to all of the above, write 'I declare that I have no competing interests' below. If your reply is yes to any, please give details below.

I declare that I have no competing interests

I agree to the open peer review policy of the journal. I understand that my name will be included on my report to the authors and, if the manuscript is accepted for publication, my named report including any attachments I upload will be posted on the website along with the authors' responses. I agree for my report to be made available under an Open Access Creative Commons CC-BY license (http://creativecommons.org/licenses/by/4.0/). I understand that any comments which I do not wish to be included in my named report can be included as confidential comments to the editors, which will not be published.

I agree to the open peer review policy of the journal
